# Self-Organization and Information Processing: From Basic Enzymatic Activities to Complex Adaptive Cellular Behavior

**DOI:** 10.3389/fgene.2021.644615

**Published:** 2021-05-21

**Authors:** Ildefonso M. De la Fuente, Luis Martínez, Jose Carrasco-Pujante, Maria Fedetz, José I. López, Iker Malaina

**Affiliations:** ^1^Department of Nutrition, CEBAS-CSIC Institute, Murcia, Spain; ^2^Department of Mathematics, Faculty of Science and Technology, University of the Basque Country, UPV/EHU, Leioa, Spain; ^3^Basque Center of Applied Mathematics (BCAM), Bilbao, Spain; ^4^Department of Cell Biology and Histology, Faculty of Medicine and Nursing, University of the Basque Country, UPV/EHU, Leioa, Spain; ^5^Department of Cell Biology and Immunology, Institute of Parasitology and Biomedicine “López-Neyra”, CSIC, Granada, Spain; ^6^Department of Pathology, Cruces University Hospital, Biocruces-Bizkaia Health Research Institute, Barakaldo, Spain

**Keywords:** entropy, dissipative structures, self-organization, Hopfield dynamics, information processing

## Abstract

One of the main aims of current biology is to understand the origin of the molecular organization that underlies the complex dynamic architecture of cellular life. Here, we present an overview of the main sources of biomolecular order and complexity spanning from the most elementary levels of molecular activity to the emergence of cellular systemic behaviors. First, we have addressed the dissipative self-organization, the principal source of molecular order in the cell. Intensive studies over the last four decades have demonstrated that self-organization is central to understand enzyme activity under cellular conditions, functional coordination between enzymatic reactions, the emergence of dissipative metabolic networks (DMN), and molecular rhythms. The second fundamental source of order is molecular information processing. Studies on effective connectivity based on transfer entropy (TE) have made possible the quantification in bits of biomolecular information flows in DMN. This information processing enables efficient self-regulatory control of metabolism. As a consequence of both main sources of order, systemic functional structures emerge in the cell; in fact, quantitative analyses with DMN have revealed that the basic units of life display a global enzymatic structure that seems to be an essential characteristic of the systemic functional metabolism. This global metabolic structure has been verified experimentally in both prokaryotic and eukaryotic cells. Here, we also discuss how the study of systemic DMN, using Artificial Intelligence and advanced tools of Statistic Mechanics, has shown the emergence of Hopfield-like dynamics characterized by exhibiting associative memory. We have recently confirmed this thesis by testing associative conditioning behavior in individual amoeba cells. In these Pavlovian-like experiments, several hundreds of cells could learn new systemic migratory behaviors and remember them over long periods relative to their cell cycle, forgetting them later. Such associative process seems to correspond to an epigenetic memory. The cellular capacity of learning new adaptive systemic behaviors represents a fundamental evolutionary mechanism for cell adaptation.

## Introduction

The cell is a highly self-organized dynamic molecular system that constitutes the basic fundamental unit of all known life forms.

A breakthrough has been made in the knowledge of the cellular molecular components and understanding the basic mechanisms of many of their interactions. However, we still do not know many of the essential aspects of the highly self-ordered dynamics that characterize the biochemical life of cells.

All molecular components of cells exhibit complex chemical transformations shaping an extreme super complex system, where simultaneously the concentrations of millions of ions and molecules change permanently following high-ordered patterns, structured in space and in time.

The consequences of these incessant self-organized and self-regulated dynamics of reactive molecular transformations are adequate cellular growth, the development of all physiological processes to maintain its functional structures, continuous adaptation to the environment, and, lastly, mitosis. Alterations in these self-organized dynamics lead to different pathologies and, on certain occasions, the dynamic order collapses and cell dies.

The limitations in the study of cellular molecular dynamics by traditional methods have been compensated by the efforts of quantitative sciences applied to Biology, especially Systems Biology. Physical-mathematical approaches are making possible to understand the principles underlying the complex cellular molecular dynamics adequately, so concepts such as self-organized dissipative structures, self-regulation, the emergence of functional properties, systemic attractors, and information processing, are part of the new landscape of contemporary biology.

In light of these quantitative advances, each cell is not a mere molecular aggregate but a super complex dynamic system where highly self-ordered dissipative patterns, the main source of molecular order in the cell, do emerge together with self-regulatory behaviors originated by biomolecular information processing, another fundamental source of organization in the cell.

Here, we present an overview of the main sources of biomolecular order and complexity that underline the molecular dynamics of cells. These sources span from the most elementary levels of molecular activity to the emergence of systemic behaviors.

- In the “The Dynamics Originated by the Molecular Turnover and the Role of the Enzymes in These Super Complex Dynamics” section, we have approached two fundamental issues: the dynamics originated by the molecular turnover of cells and the role of the enzymes in these super complex dynamics.

All molecular components of the cell are synthesized and degraded continually following sophisticated interdependent processes that defy the human intellect. This continuous molecular turnover is produced by a super complex dynamic system formed by millions of biochemical reactions, which occur continuously at every moment of cellular life. The continuous recycling and incessant chemical transformations, that encompass practically all molecules, shape a critical scenario, only in which cellular life is possible (De la Fuente, 2015).

The super complex dynamics originated by this huge molecular turnover constitute the fundamental systemic characteristic of all basic life units.

The specialists of the life sciences and those of the quantitative sciences, attracted by the biological fact, should adequately understand the fundamental molecular dynamics of the cell, and the surprising magnitude that they reach at a global level.

Numerous works have been carried out on molecular recycling, mainly of proteins (Mathieson et al., 2018; Ross et al., 2020). However, the study of recycling other molecular components has been much less addressed. Besides, most of these works have not been thoroughly considered comprehensively. Here, for the first time, we present an integral review of the molecular turnover that includes the proteome, lipidome, glycome, metabolome, transcriptome, and main cellular structures.

All reactive molecular transformations occurring in the cell are essentially chemical reactions, that is, modifications on how atoms are bonded. Practically, all these dynamics of chemical changes in the cell are mediated by enzymes, the most extraordinary macromolecular nanomachines, responsible for breaking and joining covalent bonds, the fundamental chemical bonds in biological molecules. Through their ability to decrease the bond activation energy, enzymes permanently modify the way atoms are bonded, making possible the accelerated creation and destruction of molecules. Accordingly, enzymes are fundamental and essential molecules for metabolic life. They are the main focus of attention of this review.

Without understanding the meaning and magnitude of the dynamic molecular turnover that occurs in all living cells, as well as the fundamental role of enzymes in these permanent *metabolic* dynamics (which means activities developed by enzymes), it is not possible to properly understand what a cell is, nor the essential elements that underlie in its self-ordered and self-regulated functionality.

- Section “Dissipative Self-Organization of Enzymatic Activities, the Main Source of Molecular Organization in the Cell” addresses the dissipative self-organization of enzymatic activities, the main source of molecular organization in the cell.

Enzymes are responsible for molecular recycling processes shaping multienzyme complexes (reversible structures formed by several enzymes of a metabolic pathway), which carry out their activity with autonomy between them playing distinctive and essential roles in cellular physiology. When a multienzyme complex operates far enough from equilibrium dissipative self-organization can emerge (Goldbeter, 2007; De la Fuente, 2010, 2014).

Here, we show the main enzymatic dissipative structures: biomolecular oscillations (metabolic rhythms). Even though self-organized structures are very diverse, and we also cover other different dissipative patterns including circadian rhythms, birhythmicity, multi-stability, and spatial traveling waves.

Numerous physical-mathematical analyses of enzymatic pathways have contributed to a better understanding of the emergence of self-organized processes. They are essential to research the main source of molecular organization in the cell. Most of these functional biochemical studies have been carried out using systems of differential equations e.g., the Krebs cycle (Mogilevskaya et al., 2006), the amino acid biosynthetic pathways (Yang et al., 2005), the oxidative phosphorylation subsystem (Korzeniewski and Zoladz, 2001), the glycolytic subsystem (Bier et al., 1996), the transduction in G-protein enzyme cascade (Kass and Bray, 1996), the gene expression (Gonze et al., 2004), the ascorbate-glutathione cycle in chloroplasts (Valero et al., 2016), and the cell cycle (Tyson, 1991). On the other hand, more complex analyses have been performed using non-ordinary differential equations, for example in the yeast glycolytic pathway (De la Fuente et al., 1995). In these studies, different attractor dynamics were analyzed linked to Hopf bifurcations (De la Fuente et al., 1996a), tangent bifurcations (De la Fuente et al., 1996b), the classical period-doubling cascade preceding chaos (De la Fuente et al., 1999a), persistent behaviors (De la Fuente et al., 1998a, b, 1999b) and the multiplicity of coexisting attractors in the phase space (De la Fuente et al., 1998c; De la Fuente, 1999).

The dissipative structures that emerge in self-organized multi-enzymatic complexes constitute one of the most genuine properties of cells, and the rigorous knowledge of their nature and significance is an essential element in the comprehension of the biological fact at its most basic and elementary levels.

- Dissipative metabolic networks (DMN) and the emergence of the systemic metabolic structure (SMS) are covered in the “Dissipative Metabolic Networks and the Emergence of the Systemic Metabolic Structure” section.

Enzymes form basic functional metabolic subsystems (multienzyme complexes) for turnover activities where dissipative patterns emerge. At a higher level of complexity, they shape different DMN, which originate the emergence of the SMS at a global level.

To research the functionality of the cellular metabolism (the set of activities of all enzymes) at a systemic level, taking into account the self-organized behavior, DMN was created in 1999 (De la Fuente et al., 1999b). Essentially, a DMN is an open system formed by a given set of self-organized multi enzymatic complexes interconnected by biochemical substrate fluxes and three classes of biomolecular regulatory signals: activatory (positive allosteric modulation), inhibitory (negative allosteric modulation), and all-or-nothing type (which correspond to the regulatory enzymes of covalent modulation). The SMS was observed for the first time after performing an exhaustive numerical analysis with several millions of different DMN (De la Fuente et al., 1999b, 2008). Such systemic organization is mainly defined by a small set of different self-organized multienzyme complexes which are always in active states (metabolic core), while the rest of dissipative multienzyme subsystems exhibit on-off dynamic states. Later, this global enzymatic system was verified using flux balance analysis in several prokaryotes and eukaryote cells such as *Saccharomyces cerevisiae*, *Helicobacter pylori*, and *Escherichia coli* (Almaas et al., 2004, 2005; Almaas, 2007).

Adenylate energy system constitutes another global process responsible for the functional enzymatic integration in the SMS at a global level. A primary systemic ratio between ATP, ADP, and AMP concentrations was proposed by Atkinson (Atkinson and Walton, 1967) to calculate the energetic cellular level, which was denominated the Adenylate Energy Charge (AEC).

Cellular energy quantification shows that almost all organisms keep their AEC within narrow values under growth conditions, more specifically between 0.7 and 0.95, despite the extreme complex fluctuations in the adenine nucleotide concentrations.

Further quantitative studies have shown that each cell is a complex non-linearly open system in which there is not a specific energy value that is conserved, but rather dynamic forms of energy change (De la Fuente et al., 2014). Specifically, intensive experimental measurements, which under growth cellular conditions, have shown that AEC changes between 0.7 and 0.95 are invariantly maintained in practically all classes of cells which seems to represent a functional integration feature common to all cellular organisms.

- The “Enzymatic Information Processing, the Second Leading Source of Molecular Order in the Cell” section of this review deals with enzymatic information processing: the second fundamental source of molecular order in the cell.

Enzyme activity during molecular turnover not only originates the emergence of high self-organized dissipative patterns, structured in space and time, but is also capable of producing complex self-regulatory behavior by information processing which highly increases the functional complexity of cellular system. The sophisticated cycles of construction and destruction of the molecular components could not be accomplished adequately without this source of new information, order, and organization.

The first quantitative work on the processing of molecular information in bits, in a multi-enzymatic complex, was performed in 2012 in the yeast glycolysis (De la Fuente and Cortés, 2012), one of the most analyzed dissipative multienzyme system, which was studied using TE.

On the other hand, a dynamic structure of information processing appears in the DMN when TE is considered under systemic activity (De la Fuente et al., 2011). According to these results, the DMN behaves as a highly complex decentralized information processing super-system that generates molecular information flows between the self-organized enzymatic sets, defining flows of biochemical instructions, at each moment, that make every enzymatic activity to evolve with a precise and particular turnover pattern.

Along with these works, different biological examples are displayed in the text showing that unicellular organisms possess a complex self-regulatory behavior originated by biomolecular information processing, the second fundamental source of organization in the cell.

- Finally, in the “Hopfield-Type Systemic Attractors in Dissipative Metabolic Networks. Cellular Systemic Behaviors” section, the emergence of Hopfield-type systemic attractors in DMN is analyzed. It is necessary to underline that these attractors allow the cellular metabolic structure (CMS) to behave as an individual and complete integral system. As a consequence, cellular systemic behaviors occur.

The emergence of Hopfield-type dynamics was quantitatively observed using advanced tools of Statistic Mechanics and techniques of Artificial Intelligence in complex enzymatic dynamics (De la Fuente et al., 2013). Hopfield-like dynamics are characterized by manifesting associative memory (Hopfield, 1982), and the systemic enzymatic study was the first quantitative evidence that an associative memory can emerge in an individual cell. Such memory would be a manifestation of emergent properties underlying the complex dynamics of the systemic cellular metabolic networks when dissipative enzymatic self-organization and molecular information processing act together.

In continuation of this systemic enzymatic study (De la Fuente et al., 2013), and following Pavlov’s methodological approach with dogs (Pavlov, 1927), it was observed that two different unicellular organisms (*Amoeba proteus* sp. and *Metamoeba leningradensis* sp.) showed associative learning behaviors (De la Fuente et al., 2019a). To analyze such conditioned behavior in amoebae an electric field was used as a conditioned stimulus and a specific chemotactic peptide as an unconditioned stimulus. The migratory trajectories of more than 700 amoebae under different experimental conditions were studied, and the results showed that the unicellular organisms were able to learn new systemic behaviors, which can be considered as a rudimentary form of associative memory, crucial to govern properly cellular migration. This quantitative study demonstrated, for the first time, that associative memory was also possible in unicellular organisms. Such a type of memory could be the manifestation of the emergent properties underlying the complex dynamics of the systemic metabolic networks and their Hopfield-like attractors.

One of the most important goals of contemporary biology is to understand the fundamental principles and quantitative laws governing the functional metabolic architecture of the cell. In this review, we have mainly focused on the role of dynamic enzymatic processes, the mechanisms implicated in self-organization (dissipative structures), the self-regulation of metabolic dynamics (molecular information processing), and the emergence of the cellular systemic properties (Hopfield-like dynamics). All these issues are essential to correctly understand what a cell is.

To help life scientists who are unfamiliar with quantitative sciences understand these fundamental multidisciplinary issues, we have not used mathematical formulations here. Besides, we have reinforced the fundamental ideas with a considerable number of biological examples.

Systems Biology is being increasingly used as an appropriate methodology to address dynamic metabolic processes and the higher-level properties emerging in the cell from interactions between its elementary molecular parts, forming complex self-organized and self-regulated structures, from basic enzymatic activities to complex cellular adaptive behavior.

Systems biology is fundamental to understand the functional architecture of the most complex, sophisticated, and overwhelming molecular system known in nature, the living cell.

## The Dynamics Originated by the Molecular Turnover and the Role of the Enzymes in These Super Complex Dynamics

Cells are dynamic metabolic reactors in which millions of biochemical reactions, tightly interrelated and integrated into sophisticated networks, shape the most complex molecular system in nature.

All molecular components of the cell are in a dynamic state of reactive transformations. The proteome, lipidome, glycome, metabolome, and transcriptome are synthesized and degraded continually in all cell types (see Supplementary Material 01 for further details). Even outside the growth period, the intracellular macromolecular pool is dynamic, and considerable energy is expended in the continuous processes of synthesis and degradation. For instance, only the protein turnover accounts for 38–47% of the total energy produced in every cell (Lahtvee et al., 2014).

Not only molecules but also cellular structures are subjected to cycles of construction and destruction. For instance, the endoplasmic reticulum, the largest endomembrane system, exhibits a permanent turnover; the cytoskeleton is another molecular dynamic system that undergoes continuous reorganization through the synthesis and degradation of its structural elements during the cell cycle; the mitochondria are dynamic organelles that are incessantly undergoing fusion, fission, and molecular destruction; the peroxisome turnover also takes place by autophagy-related mechanism; also, cellular membranes are highly reactive dynamic structures in non-stop recycling, so, in 30 min, an active cell such as a macrophage recycles by endocytosis and exocytosis an amount of plasma membrane that equals its complete plasma membrane (see Supplementary Material 01).

From a molecular perspective, each cell is in a permanent state of self-construction and self-destruction. This is the fundamental systemic characteristic of all basic units of life. The continuous molecular recycling that defines the systemic cellular functionality relies on a super complex dynamic system formed by millions and millions of biochemical reactions which occur continuously, at every moment of the cellular life.

Enzymes are the essential functional molecules of this super complex dynamic reactor. They are responsible for most of the molecular transformations, and such enzymatic activity, when considered globally, is called cellular *metabolism* (Stryer et al., 2002). Almost all enzymes are proteins; however, some RNAs, called ribozymes, also exhibit catalytic activity (Stryer et al., 2002). As a piece of metabolic activity, some non-enzymatic reactions also occur, which are important for the evolution of metabolic pathways (Keller et al., 2015).

Almost all molecular synthesis and destruction processes need to be catalyzed so that they occur at adequate fast rates to sustain cellular life. These catalytic activities involve the formation and breakdown of covalent bonds, i.e., the creation or destruction of molecules. Enzymes are accelerators of biochemical reactions. They speed up the catalytic processes in different forms, all of which decrease the activation energy of bonds (Stryer et al., 2002). As a result, the rates of enzymatic processes are accelerated from thousands to million-fold, so biochemical reactions that would take years in the absence of catalysis can occur in fractions of seconds inside the cell. For instance, a very fast catalytic process is propitiated by orotidine 5′-phosphate decarboxylase which allows the decarboxylation of orotic acid, an intermediate in the biosynthesis of pyrimidine nucleotides, in milliseconds under cellular conditions, while this reaction takes place with a half-time of 78 million years under raw natural conditions (Radzicka and Wolfenden, 1995).

Metabolism can be classified into two fundamental classes: catabolic processes (the breakdown of molecules that usually results in the release of energy and smaller essential molecular parts) and anabolic processes (the synthesis of molecules such as proteins, lipids, glycans, nucleic acids, etc., from more minor molecular elements). These anabolic and catabolic reactions ensure molecular recycling in the cell.

Enzymes are the biomolecules that perform the most important biological cellular functions. Unlike the rest of the biomolecules, enzymes are not passive. They are the only ones that can carry out a catalytic activity, transforming some molecules into others and developing complex interrelated reactive dynamics (the enzymatic networks, essential to the functionality of cell physiology). These biocatalysts are responsible for the formation of all the ATP in the cell, DNA synthesis, apoptosis, mitosis, cytoskeleton organization, DNA repair, alternative splicing, chromosome regulation, gene expression, post-translational modifications, Golgi apparatus activity, etc. In short, all the main activities in the cell are mediated by enzymes. They constitute the fundamental elements of all essential physiological processes, which allow a cell to grow, multiply, and adapt to the external medium. However, being biologically essential, many issues of enzyme activities remain poorly understood which warrant further investigation.

There are a lot of important biomolecules in the cell, such as ATP, nucleotides, lipids, structural proteins, etc., but enzymes constitute the unique active biomolecules with the capacity to reordering the atoms, i.e., the ability to transform some molecules into others creating or destroying bonds between atoms.

Genes encode information about the primary structure of each enzyme, and this amino acid sequence determines its *catalytic specificity*. However, the *enzymatic functionality*, the set of different patterns of activity carried out by every enzyme, cannot be predicted from the primary structure alone. Enzymes are not rigid molecular structures; they have complex dynamic conformations and are continuously undergoing a wide range of conformational fluctuations which modify their catalytic activities. Even in the native state, an enzyme exhibits a range of complex interconverting conformations driven by thermodynamic fluctuations (Ramanathan et al., 2014; Agarwal, 2019). Besides, the dynamic changes in the hydration shell rapidly control these conformational fluctuations of enzymes (they can be described by an energy landscape) that hinder the connection between enzyme sequence and its catalytic function (Fenimore et al., 2004; Dunaway-Mariano, 2008).

**FIGURE 1:**
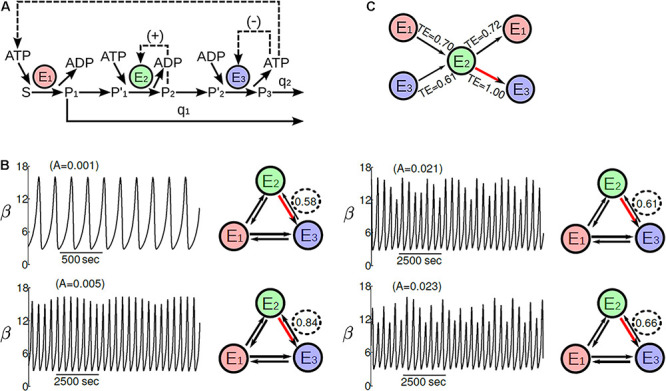
Molecular information processing in yeast glycolysis. **(A**) The main irreversible enzymatic reactions of the dissipative multi enzymatic system are E_1_, E_2_, and E_3_ which correspond, respectively, to hexokinase, phosphofructokinase, and pyruvate kinase. S, P_1_, P′_1_, P_2_, P′_2_, and P_3_ represent, respectively, glucose input source, glucose-6-phosphate, fructose 6-phosphate, fructose 1,6-biphosphate, phosphoenolpyruvate, and pyruvate. The first-order rate constant for the removal of glucose-6-phosphate and pyruvate are respectively q_1_ and q_2_. The main instability mechanism for dissipation is the complex regulation of the phosphofructokinase enzyme. This self-organized multienzyme system exhibits a classic *quasi-periodic route to chaos* under specific conditions of glucose input-flux (De la Fuente et al., 1996a). Thus, **(B)** the phosphofructokinase enzyme activity (E_2_), measured by the fructose 1,6-biphosphate concentration along the time, shows a transition sequence with a stable periodic pattern, quasi-periodic rhythms, complex quasi-periodic oscillations, and deterministic chaos (Ruelle-Takens-Newhouse route). The panels on the right illustrate the information processing that regulates these complex behaviors. Edge thicknesses are proportional to the information bits divided by the maximum value of transfer entropy, which corresponds to the red edge. **(C)** Normalized total information flows in the self-organized glycolytic system during the quasi-periodic route to chaos. The glycolytic system is self-regulating against external perturbations (glucose inputs) through molecular information processing, adapting the enzymatic patterns to alterations coming from outside the system (here, the external source of glucose). Part of this figure has been reported previously by De la Fuente and Cortés (2012).

**FIGURE 2:**
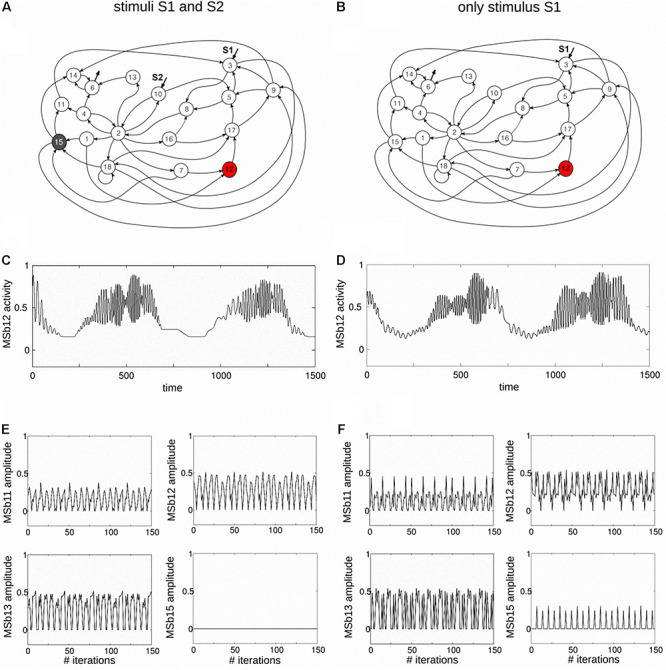
Dynamic catalytic patterns emerge in a dissipative metabolic network. A self-organized metabolic network shaped by 18 dissipative multienzyme complexes, interconnected by different substrate fluxes, was subjected to two external conditions: **(A**) two inputs of substrate sources S1–S2, and **(B**) one input of external substrate source S1, in the right panel. As a result of the network activity, the multienzyme complex MSb12 was permanently active under both external conditions (metabolic core), MSb15 was inactive under the two inputs of substrate sources S1–S2, whereas it was intermittently active under one external stimulus. Other dissipative multienzyme sets exhibited on-off changing activity states. The dissipative network is an open system and MSb6 activity ends outside the metabolic network. In the network, these self-organized multienzyme complexes autonomously exhibit many enzymatic transitions (more than 350) between different spontaneous periodic oscillations and steady-states when they are active. Thus, two examples of MSb12 catalytic activities are presented in panel **(C)**, under two sources of external substrates S1-S2, and in **(D)** under one stimulus S1. Different changes in the amplitude of the dissipative catalytic activities of the multi enzymatic sets can be observed in panel **(E,F**), which show the complex enzymatic patterns that emerge in the dissipative metabolic network under these cellular conditions. Part of this figure has been reported previously by De la Fuente et al. (2011).

Besides, the activities of most enzymes are not constant in biological environments; instead, catalytic activity can be complexly regulated thus emerging sophisticated activity patterns (Figure 1). These enzymatic behaviors depend on different factors, mainly substrate concentration, and any inhibitors or activators. The substrate fluxes and regulatory molecular flows form complex dynamical networks and, as a result, all metabolite concentrations permanently change with time due to collective interactions between them. Therefore, metabolite concentrations are a function of self-organized dynamics that emerge in the enzymatic networks (see below). Such collective connectivity integrated into biochemical networks makes highly variable the rate at which catalytic reactions proceed (see Figure 2), being able to exhibit infinite patterns of activity (De la Fuente, 2014, 2015). It should be noted that a great number of fundamental elements of the enzymatic activity under cellular conditions, continue to be incomplete after decades of research (Ringe and Petsko, 2009).

Practically all physiological processes rely on the enzymatic activity which shapes the cellular metabolism. This is an essential issue to understand the basic principles of biochemical functionality at all levels of its cellular architecture (Noble et al., 2014). Knowing the cellular metabolism is necessary to understand the laws and principles that determine cellular functions as a whole.

### Enzymatic Self-Organization at the Molecular Level

How are enzymatic reactions organized in cells?

Over decades it was widely assumed that enzymes were randomly distributed in the intracellular medium. However, numerous researches on protein-to-protein interactions have demonstrated that homologous or heterologous protein-protein interactions shape complex macromolecular associations (Pang et al., 2008). More precisely, yeast proteome analysis has established that up to 83% of all proteins constitute supra-molecular associations (Gavin et al., 2002), and most of the cellular processes are not carried out by isolated proteins, but rather by multiprotein complexes (Gavin and Superti-Furga, 2003; Kastritis and Gavin, 2018). Such associative protein organization occurs in both eukaryote and prokaryote cells (Ho et al., 2002; Kühner et al., 2009).

### Multi Enzymatic Associations

Different approaches have been applied to detect and study protein sets, such as advanced experimental molecular biology procedures, bioinformatics techniques, statistical physics tools, and computational methods (SabziNezhad and Jalili, 2020; Sartori and Leibler, 2020).

Of all protein complexes, enzyme-enzyme complexes are the most essential for biological functionality. The basic concept of multienzyme associations was initially suggested by A. M. Kuzin in the seventies of the last century (Kuzin, 1970). Besides, Paul A. Srere was the first to observe a non-trivial supramolecular enzymatic organization in the citric acid cycle (Srere, 1972), who later called “metabolon” to all these types of multienzyme sets that can catalyze two or more sequences of a metabolic route (Srere, 1985, 1987).

The multienzyme complex is a functional reversible superstructure formed by several enzymes of a metabolic pathway that uses non-covalent interactions such as hydrogen bonding, cation-pi interaction, and hydrophobic force to maintain ensemble several catalytic elements (Figure 1). Such enzymatic complexes allow highly selective reactions, increasing their catalytic efficiency (Conrado et al., 2008; Araiza-Olivera et al., 2013).

Many enzymes belonging to metabolic pathways that shape these complex supramolecular structures have been studied, for instance, tryptophan synthase complex (Hilario et al., 2016), fatty acid synthase (Maier et al., 2006), glycolysis (Kohnhorst et al., 2017), Calvin cycle (Gontero et al., 2018), cellulosome (Dou et al., 2015), pyrimidine biosynthesis (Serre et al., 1998), purine biosynthesis (Hoskins et al., 2004), synthesis of 5′-phosphosulfate (Sun and Leyh, 2006), aldolase dehydrogenase complex (Carere et al., 2011), polyketide synthases (Tsai and Ames, 2009), proteasome (Jung and Grune, 2012), pyruvate dehydrogenase (Izard et al., 1999), carbamoyl phosphate synthetase (Thoden et al., 1997), alpha-ketoglutarate dehydrogenase (Tretter and Adam-Vizi, 2005), urea cycle (Watford, 1989), and aldehyde–alcohol dehydrogenase (Kim et al., 2020).

A multienzyme complex provides efficient advantages over individual enzymes. In many observations, multienzyme sets can transfer the metabolites from one enzyme to the active site of the next consecutive enzyme without diffusion into the bulk medium, avoiding their eventual loss (*substrate channeling*). The time of diffusion between successive catalytic steps is minimized. This non-covalent direct transfer process of metabolic intermediates originates faster catalysis, making the catalysis more efficient in the multienzyme sets (Ovádi and Srere, 2000; Ovádi and Saks, 2004; Castellana et al., 2014). *Substrate channeling* may occur in several ways i.e., across enzyme channels or over the electrostatic surface of the protein superstructure (Sweetlove and Fernie, 2018; Svedružić et al., 2020; Zhang and Fernie, 2020).

Also, intracellular membranes and structural proteins may reversibly interact with enzymatic sets originating microcompartments, which represent another advantage of multienzyme complexes (Saks et al., 2007, 2009; Monge et al., 2009). Thus, it has been observed in many cases that enzymes activity is produced in these small specialized microenvironments, limited either by surface or restricted by volume, which is more stable against the biochemical changes in the intracellular medium, allowing more efficient catalytic reactions (Araiza-Olivera et al., 2013; Küken et al., 2018).

Other pieces of evidence have shown that some metabolic compartments are liquid-like, which are formed by phase separation from the cytoplasm. Such liquid-like compartments describe non-membrane-bound restricted spaces as phase-separated, also creating small specialized microenvironments (Hyman et al., 2014).

In the global molecular crowding that constitutes the cell, enzymes rarely function in isolation, but rather as components of catalytic sets and interdependent coupled bioprocesses (Greco and Cristea, 2016).

## Dissipative Self-Organization of Enzymatic Activities, the Main Source of Molecular Organization in the Cell

Cells are open dynamic metabolic reactors that exchange matter and energy with the external environment, obtaining fundamental elements for survival from the nutrients through multi-enzymatic reactions. All living organisms exhibit highly ordered enzymatic dynamic processes and use the energy of nutrients to maintain non-equilibrium states, where sophisticated structures, complex biochemical patterns, and collective biomolecular self-organization emerge. When any cell reaches equilibrium with the external medium, its characteristic high functional and molecular order disappears and it dies.

Thermodynamics is a necessary field of scientific knowledge to properly understand some essential principles of biochemical processes (von Stockar, 2010; Kondepudi and Prigogine, 2014). So, entropy is an important concept that allows quantification of uncertainty and disorganization in a system. In isolated systems, entropy never decreases. Conversely, it increases toward a maximum at equilibrium. In open systems, which exchange energy and matter with their environment, entropy can be sustained or decrease, avoiding a state of thermodynamic equilibrium. The negative variation (producing a consequent increase in the entropy of the surroundings) corresponds to a relative order inside the system. This is possible by the dissipation of energy i.e., the energy becomes not only unavailable but irrecoverably lost in the surroundings through irreversible processes (Niebel et al., 2019). The tendency of an open system that consumes energy with low entropy to dissipate energy with higher entropy to its surroundings (thus, the system succeeds in liberating itself of part of its own produced entropy) can allow self-organization of the system (Ebeling and Ulbricht, 1986). This principle is fundamental to understand the main source of molecular order in the cell.

The theoretical basis of self-organization was formulated in 1977 by the Nobel Prize Laureate in Chemistry Ilya Prigogine in his work on dissipative structures (Nicolis and Prigogine, 1977). Specifically, *dissipative self-organization* at the molecular level constitutes spontaneous highly ordered microscopic structures far from thermodynamic equilibrium which are characterized by coherent spatial and/or temporal patterns. Due to complex non-linear interactions among molecular components and driven by energy dissipation, these self-organized structures emerge increasing the structural and functional complexity of the system. Such emergent self-organized dynamic structures in space and time are called self-organized dissipative structures (Nicolis and Prigogine, 1977; Kondepudi and Prigogine, 2014).

Dissipative self-organized processes use the energy inflow to generate a negative entropy variation in the open system which corresponds to an emergent positive increment in the information self-contained in the system. Such information increases the complexity, being able to produce highly-ordered macrostructures and complex functional dynamic behaviors. Non-linear interactions and irreversible processes may amplify fluctuations leading to a dynamic state, far from the equilibrium, in which the system becomes spatially and temporally self-organized (Klimontovich, 1999; Halley and Winkler, 2008; Misteli, 2009)

One of the most significant scientific discoveries of the 20th century concerns this new source of molecular order in nature, the dissipative self-organization. Such a source is critical to understand the dynamic activities of multienzyme complexes and the systemic functional organization in the cell. The work of Ilya Prigogine represents a profound and original treatment of molecular organization in nature, with deep implications for understanding biological life at its most fundamental levels.

### Dissipative Self-Organization of Multienzyme Complexes

How does the functionality of multienzyme complexes in the prevailing conditions inside the cell work?

It is well established that enzymes, in addition to form multicatalytic sets, can shape dissipative structures in which two fundamental types of dynamic self-organization can emerge: *temporal rhythms* and *spatial waves*.

When a multienzyme set operates far enough from equilibrium and dissipative self-organization emerges, all the enzymes of the complex do function as a whole, showing reactive coordination between them (long-range coherence) in such a way that all the substrate and product concentrations spontaneously start to oscillate along time (temporal rhythms), see Figure 1. As a consequence, thousands and thousands of molecules and ions that shape the multicatalytic system (substrates, products, protons, regulatory molecules, and other metabolites) experiment massive oscillatory reorganizations in their molecular concentrations. Such dynamics are mainly characterized by collective synchronized behaviors, functional correlations between molecular components separated by macroscopic distances, coherent patterns, and highly coordinated integrative processes (De la Fuente, 2010). Dissipative self-organized catalytic behaviors find their roots in the non-linear enzymatic processes which mainly involve allosteric regulation, autocatalysis, cooperativity, and feed-back interactions (Goldbeter, 2002, 2007, 2018; De la Fuente, 2014).

All metabolite concentrations of multienzyme sets that exhibit dissipative self-organization processes present transitions between complex oscillations and quasi-steady states under cellular conditions (De la Fuente, 2015). The quantification of certain intracellular molecules through nano biosensors in living cells has shown complex quasi-steady states and oscillatory patterns whose dynamics are never constant (Ozalp et al., 2010). Different experimental pieces of evidence suggest that oscillatory behaviors are much more frequent than quasi-steady states in cellular conditions (Lloyd and Murray, 2005, 2006).

Biomolecular oscillations are a genuine behavior of the complex self-organized enzymatic processes in basic units of life. Such oscillatory behavior spontaneously occurs far from thermodynamic equilibrium, when the system is formed by irreversible enzymatic processes with non-linear kinetics, Figure 1. Intracellular molecular oscillations were reported in: ATP, ADP, and AMP nucleotides (De la Fuente et al., 2014), microtubule polymerization (Lange et al., 1988) and other cytoskeletal structures (Kruse and Jülicher, 2005), cyclins (Hungerbuehler et al., 2007), cyclic AMP concentration (Holz et al., 2008), cytokinins (Hartig and Beck, 2005), free fatty acids synthesis (Getty-Kaushik et al., 2005), actin polymerization (Rengan and Omann, 1999; Negrete et al., 2016), biosynthesis of phospholipids (Márquez et al., 2004), urea cycle (Fuentes et al., 1994), proteolysis (Kindzelskii et al., 1998), glycolysis (Dano et al., 1999), metabolism of carbohydrates (Jules et al., 2005), intracellular glutathione concentration (Lloyd and Murray, 2005), Krebs cycle (Wittmann et al., 2005), mitochondrial metabolic processes (Aon et al., 2008), photosynthetic reactions (Smrcinová et al., 1998), CO_2_ (Tyson, 2002), protein kinase activities (Chiam and Rajagopal, 2007), transcription factors (Garmendia-Torres et al., 2007), respiratory metabolism (Lloyd et al., 2002), intracellular calcium concentration (Ishii et al., 2006), peroxidase-oxidase reactions (Møller et al., 1998), membrane receptor activities (Placantonakis and Welsh, 2001), membrane lipid oscillation (Nakamura, 2018), ERK/MAPK metabolism (Shankaran et al., 2009), intracellular pH (Sánchez-Armáss et al., 2006), intracellular free amino acid pools (Hans et al., 2003), membrane potential (De Forest and Wheeler, 1999), metabolism of mRNA (Klevecz and Murray, 2001), min-proteins (Wettmann and Karsten, 2018), beta-oxidation of fatty acids (Getty et al., 2000), amino acid transports (Barril and Potter, 1968), insulin secretion (Tyson, 2002), etc.

Moreover, a metabolic orchestration has been observed in cells at a systemic level, by which the entire metabolome and most of the transcriptome oscillate (Klevecz et al., 2004; Lloyd and Murray, 2006; Murray et al., 2007). More specifically, several studies have analyzed oscillatory processes in the transcriptome (Tonozuka et al., 2001; Tian et al., 2005; Chabot et al., 2007). These studies have shown that at least 60% of all transcriptions oscillate with an approximate period of 300 min (Tu et al., 2008). Microarray analysis from *S. cerevisiae* in continuous synchronous culture shows a genome-wide oscillation in transcription processes coupled to respiration with maxima of transcript levels at intervals of about 40 min (Klevecz et al., 2004).

The oscillatory periods of metabolic rhythms range from milliseconds to minutes and hours (Chance et al., 1973; Berridge and Galione, 1988; Aon et al., 2006; Brodsky, 2006; Roussel et al., 2006). Complex periodic oscillations, including bursting rhythms and deterministic chaotic phenomena, have often been detected (Olsen and Degn, 1985; Dekhuijzen and Bagust, 1996; Almeira and Gusman, 2017; Heltberg et al., 2019).

Dissipative structures are very diverse (Goldbeter, 2018). For instance, besides rhythmic metabolic behaviors, the coexistence between two stable oscillations in a biochemical set (birhythmicity) and the coexistence of several stable steady states (multistability) may also emerge in the same molecular system. Multistability has been observed in different biosystems such as the calcium/calmodulin-dependent protein kinase II (Zhabotinsky, 2000), NF-κB signaling (Pêkalski et al., 2013), apoptosis (Bentele et al., 2004), cytoskeleton control (Byrne et al., 2016), lac operon (Santillán and Mackey, 2008), and signal transduction (Xiong and Ferrell, 2003). The coexistence between two oscillatory regimes and a steady-state with periodic behavior (hard excitation) has also been observed in numerical studies of multienzyme complexes (De la Fuente et al., 1998c; De la Fuente, 1999) and chemical systems (Guttman et al., 1980).

Another class of self-organized biological dissipative processes is circadian rhythms, which exhibit an oscillatory period close to 24 h (dark–light cycle during the Earth’s rotation period). These endogenous autonomous oscillators can adapt their internal metabolism to changes in the external environment (light, temperature, food availability, etc.) during 24-h day/night cycles (Yanling et al., 2019). Circadian rhythms exist in all types of cells from prokaryotes to eukaryotes, and these dissipative patterns regulate a great variety of important physiological processes (Wijnen and Young, 2006). For instance, oscillating genes are also usually circadian (Tuttle et al., 2005). Even, it has been observed in some cells that 80–90% of the transcriptome show a rhythmic gene expression with cycles of 24–26 h (Connor and Gracey, 2011).

Circadian transcription-translation cycles are intertwined with different metabolic activities and respiratory oscillations, which ensures the systematic coordination and integration of cellular physiological processes (Causton, 2020).

Lastly, a fundamental type of dissipative structure in cells is the spatial traveling waves which consist of three-dimensional self-organized coherent oscillation of metabolite concentrations that propagates progressively across the intracellular medium, reminiscent of a wave moving across water. These wave pulses of biochemical activity, moving through subcellular domains over large intracellular distances, are too fast (5–30 μm s^–1^ for calcium waves; Jaffe, 2002) and represent an essential mechanism for long-range functional interconnection in the cell. Spatial traveling waves have a crucial role in coordination and synchronization among different metabolic processes, and these dissipative patterns exhibit other characteristics in their shape of oscillation, cellular location, and molecular composition (Guthrie et al., 1999; Scemes and Giaume, 2006; Carsten and Karsten, 2017). Some examples of spatial biochemical oscillations have been observed in calcium ions (Jaffe, 2008), actin dynamics during cell locomotion (Vicker, 2002; Allard and Mogilner, 2013), apoptotic signals (Cheng and Ferrell, 2018), mitochondrial redox (Romashko et al., 1998), NAD(P)H (Kindzelskii and Petty, 2002; Slaby and Lebiedz, 2009), sodium ions waves in astrocytes (Bernardinelli et al., 2004), phosphoprotein waves (Markevich et al., 2006), Cdk1 waves implicated in the cell cycle (Deneke et al., 2016), mitotic waves (Nolet et al., 2020), adenosine triphosphate (Ueda et al., 1990; Newman, 2001), NAD(P)H and protons (Petty et al., 2000), phosphatidylinositol (3,4,5)-trisphosphate (Asano et al., 2008), ROS molecules (Zhou et al., 2010), and mitochondria activity (Kurz et al., 2010).

Each cell is a super dynamic metabolic system in which self-construction and self-destruction of molecules occur following complex dissipative catalytic rhythms, coordinated by complex spatial traveling waves.

Notice that after more than five decades of research many aspects of self-organized processes remain poorly understood.

### Molecular Self-Assembly

Apart from dissipative structures, another mechanism of a molecular organization is self-assembly, which can be defined as the spontaneous formation of supramolecular structures usually driven by specific non-covalent interactions, yielding functional and stable three-dimensional macromolecular complexes. The forces that directly generate molecular self-assembly tend to be weak; specifically, they are non-covalent interactions such as van der Waals electric forces, hydrophobic effects, electrostatic attraction, and hydrogen bonding. Under cellular conditions, there are two classes of molecular self-assembly: static and dynamic. Static self-assembly, an important spontaneous mechanism generating molecular order, involves processes that are near thermodynamic equilibrium and do not dissipate energy. Some examples are the formation of the lipid bilayer, viral capsid, protein aggregates to hold the quaternary structure, and some supramolecular polymerization (Sartori and Leibler, 2020). Dynamic self-assembly occurs in non-equilibrium states when the system is dissipating energy. Therefore it is governed by irreversible enzyme kinetics, for example, the self-assembly of actin filaments due to hydrolysis of ATP (Whitesides and Grzybowski, 2002; Rieß et al., 2020). Both classes of self-assembly processes may also occur simultaneously with dissipative self-organization (Halley and Winkler, 2008).

Dissipative self-assembly and dissipative self-organization are the fundamental pillars of the molecular order of all living cells (De la Fuente, 2015).

### Dissipative Metabolic Networks and the Emergence of the SMS

Two main principles govern the enzymatic organization in the cell: *metabolic segregation* and *systemic metabolic integration*.

### Metabolic Segregation

All cellular metabolisms are segregated in multiple autonomous biochemical specializations. This separation of enzymatic roles demands that correlated catalytic processes are grouped. Consequently, the systemic metabolism is functionally segregated at a great variety of specialization levels, originating different types of specific catalytic activities (e.g., the lysosomal metabolism, the oxidative phosphorylation, the signal transduction, etc.).

All modular metabolic networks that shape the cell can be considered as discrete dynamic sets, which perform relatively autonomous activities with specific and coherent enzymatic functionalities. Several scientific works have demonstrated that these elemental networks shape specialized modular units showing the genuine segregation of the metabolic activity (Ravasz et al., 2002; Nurse, 2008; Hao et al., 2012; Kaltenbach and Stelling, 2012; Geryk and Slanina, 2013). Segregation networks have been detected in all principal cellular metabolic activities, e.g., “enzymatic organization of metabolic pathways (Muto et al., 2013), chaperone activities (Korcsmáros et al., 2007), chemotaxis (Postma et al., 2004; Shimizu et al., 2010), apoptosis processes (Harrington et al., 2008), cell cycle (Boruc et al., 2010; Hsu et al., 2011), Golgi apparatus (Nakamura et al., 2012), and kinetochore organization (Petrovic et al., 2014). Specific modular metabolic networks also take part in the transcriptional system such as miRNA regulatory networks (Gennarino et al., 2012), transcription factors networks (Neph et al., 2012), RNA polymerase complexes (Schubert, 2014), and mRNA dynamics (Vlasova-St Louis and Bohjanen, 2011)” (De la Fuente, 2015).

Dissipative multienzyme complexes can also be considered fundamental modular networks (De la Fuente et al., 2008; De la Fuente, 2010, 2014). In this respect, the Metabolic Subsystem concept was suggested in 1999 to define dissipatively structured enzyme complexes in which autonomous catalytic processes with complex quasi-steady-state patterns and molecular rhythms spontaneously emerge (De la Fuente et al., 1999b). Multienzyme complexes carry out specific catalytic activities that also interact reversibly with structural proteins and membranes, originating microcompartments thermodynamically open, in which biomolecular oscillations, substrate channeling, and integrative mechanisms may emerge. These functional integrative processes increase the efficiency of the self-organized multienzyme complexes. Dissipative multienzyme complexes represent highly efficient modular networks capable of performing discrete catalytic activities that allow cellular functional organization. Such self-organized multienzyme sets constitute the essential and basic catalytic elements of the cell (De la Fuente, 2015).

### Metabolic Integration

Aside from the *metabolic segregation*, catalytic activities present another essential property of organization: *systemic metabolic integration*.

Biocatalytic sets organized into distinct basic modular networks are functionally integrated with the cell through global coordination resulting from the concerted metabolic actions of the different specialized biochemical activities as a whole (Almaas et al., 2004, 2005; Yoon et al., 2007; Buescher et al., 2012; San Román et al., 2014).

**FIGURE 3:**
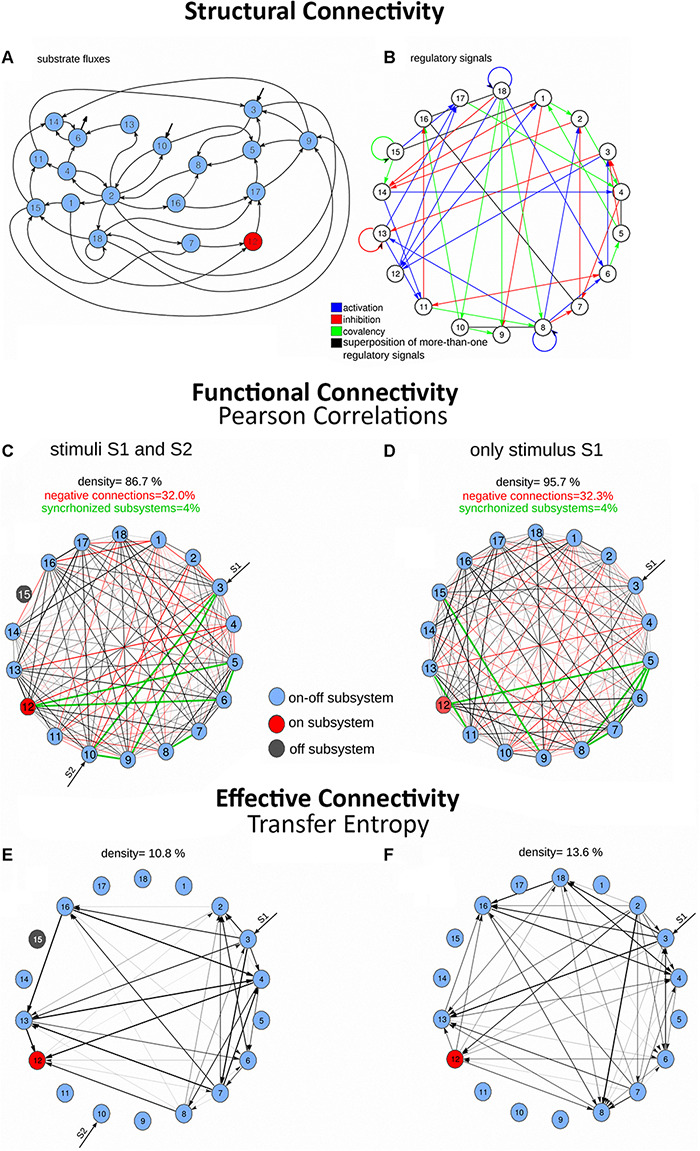
*Structural connectivity*, *functional connectivity*, and *effective connectivity* (transfer entropy) in a dissipative metabolic network (DMN). The *Structural connectivity* of the dissipative metabolic network (showed in Figure 2) are shaped by **(A)** 18 self-organized multienzyme complexes interconnected by substrate fluxes and **(B)** regulatory signals, which correspond to two types of allosteric processes (activation signals in blue and inhibitory signals in red) and covalent modifications (in green). *Functional connectivity* based on Pearson correlations of the DMN is illustrated in panels **(C,D)**. The edge thickness is proportional to the obtained correlation values. Black edges are positive correlations, negative connections are in red, and the synchronized dissipative multienzyme sets are in green. The great density of functional connectivity demonstrates the complex systemic organization of the DMN. *Effective connectivity* measured in information bits (TE) is shown in panels **(E,F)**. Edges are directed (causal relations between the multienzyme sets), and thickness is proportional to the normalized information bits. The metabolic network is capable of self-organizing through a dissipative process (exhibiting complex quasi-stationary and oscillatory catalytic patterns, see Figures 2C–F) and self-regulating versus perturbations employing the molecular information processing, adapting the dissipative metabolite patterns to alterations coming from outside the system (here, two external input of substrate sources S1–S2 in the left panels, and one external substrate source S1, in the right panels). Part of this figure has been reported previously by De la Fuente et al. (2011).

While *functional segregation* expresses the partial interdependence of specialized metabolic nets (Figure 1), *metabolic integration* is an additional principle shared by the autonomous catalytic process that reflects high deviation activities from the functional autonomy of these modular networks, in such a way that the collective metabolic elements of the cell produce coherent systemic dynamics (Figures 2, 3) that are functionally integrated, shaping a super-complex systemic dynamic structure: the *SMS* (De la Fuente, 2015).

The SMS was observed for the first time in 1999 in an exhaustive numerical analysis with several millions of different DMN (De la Fuente et al., 1999b). Such systemic organization is defined by a small set of different self-organized multienzyme complexes which always present active states, while the rest of dissipative multienzyme subsystems exhibit on-off active states (Figures 2, 3). Under this integrative metabolic activity, each active multicatalytic subsystem generates output responses with complex dynamic transitions between steady states and oscillatory patterns. The set of multienzyme complexes always within an active state are called the metabolic core (De la Fuente et al., 2008).

Later, this global enzymatic system was verified employing flux balance analysis in several prokaryotes and eukaryote cells such as *S. cerevisiae*, *H. pylori*, and *E. coli*. Besides, essential enzymatic reactions for biomass formation were observed in the metabolic core (Almaas et al., 2004, 2005).

Adenylate energy system constitutes another global process responsible for the functional integration of cellular metabolism. Energy is an essential element to keep the metabolic architecture of cells, and adenosine nucleotides couple all bio-energetic enzymatic processes one each other. The dissipative multienzyme complexes regulate their functional activity through changes in ATP, ADP, and AMP levels, along with other biochemical factors.

The adenosine nucleotide levels are determined by the adenylate energy system at a systemic level. Such dynamic energetic structure is shaped by enzymatic reactions which interconvert AMP, ADP, and ATP, as well as energy-consumption processes coupled to the synthesis of ATP (De la Fuente et al., 2014).

Five decades ago, a primary systemic ratio between ATP, ADP, and AMP concentrations was proposed by Atkinson (Atkinson and Walton, 1967) to calculate the energetic cellular level, which was denominated the AEC. Cellular energy quantification shows that almost all organisms appear to keep their AEC within narrow values under growth conditions, more specifically between 0.7 and 0.95, despite the extreme complex fluctuations in the adenine nucleotide concentrations in the cell. This quantitative physiological invariant represents a key property of the integrative mechanisms of energetic and functional dynamics operating at the systemic cellular level that is supported by numerous experimental researches (De la Fuente et al., 2014).

In short, two essential principles govern the different ways of enzymatic organization in the cell: *metabolic segregation* and *systemic metabolic integration*. Both are not excluding, but complementary, and the interplay between them represents a fundamental property for the systemic cellular metabolism and its functional global architecture.

On the other hand, in modular and systemic networks, three main issues of metabolic connectivity can also be considered: structural connectivity (structural molecular links), functional connectivity (non-causal statistical dependencies), and effective connectivity (causal dependencies), see an example in Figure 3 (De la Fuente et al., 2011, 2013; De la Fuente and Cortés, 2012).

## Enzymatic Information Processing: The Second Primary Source of Molecular Order in the Cell

A significant number of experimental and numerical works have shown that basic units of life exhibit complex self-regulatory behaviors originated by biomolecular information processing, the second essential source of order and organization in cells.

### Self-Regulation in Dissipative Multienzyme Complexes

According to the Information Theory, TE permits quantifying, in bits, the information of biomolecular patterns (see Supplementary Material 02 for more details) (Schreiber, 2000). The information processing in yeast glycolysis, a dissipative multienzyme complex, was studied in 2012 using TE (De la Fuente and Cortés, 2012). This biochemical pathway is one of the most analyzed and it has been considered a classical prototype of a metabolic oscillator (see Supplementary Material 02). In Figure 1A, the main irreversible enzymatic processes and the basic structural connectivity of the yeast glycolytic pathway are shown. Such metabolic subsystem under a specific glucose source allows observing a quasi-periodicity route to chaos (Ruelle-Takens-Newhouse route). Figure 1B displays a transition sequence of this route with a stable periodic pattern, quasi-periodic rhythms, complex quasi-periodic oscillations, and deterministic chaos (De la Fuente et al., 1996a).

The molecular information from these glycolytic oscillatory processes was captured by TE. Specifically, the information values in bits were normalized with the maximum TE value obtained, so the value of 1.00 represents the TE value between the functional connection of phosphofructokinase and pyruvate kinase enzymes which is the leading source of information in the glycolytic pathway (De la Fuente and Cortés, 2012). The functional influence obtained ranged from 0.58 ≤ *TE* ≤ 1.00 (with mean ± SD = 0.79 ± 0.12), which suggests highly effective connectivity in the dissipative multienzyme subsystem. The analysis also indicated that the flows of functional connectivity (contained in the different metabolite patterns) change in all catalytic transitions studied (see Figure 1B), under other inputs of glucose concentrations. The leading source of molecular information coincided with the phosphofructokinase enzyme at the edge of deterministic chaos, when complex quasiperiodic patterns in the glycolytic pathway appeared. This is consistent with other investigations: when a dynamic system works on the border between deterministic chaos and periodic order, its complexity reaches its maximum (Kauffman and Johnsen, 1991; Bertschinger and Natschlager, 2004).

The difference between the total TE input minus the TE output in each irreversible enzyme was calculated to study the total information flow of the metabolic subsystem. From these differences, negative values indicated targets of causality flow, while positive numbers were interpreted as sources. The highest value of total transfer information corresponded to the phosphofructokinase enzyme (0.41), and it occurred with the emergence of complex quasiperiodic patterns. The phosphofructokinase (E2) was the principal source of molecular information in the system, and the pyruvate kinase worked as a sink, which corresponds to a functional target; the irreversible enzyme hexokinase appeared with a flow close to zero and less constrained (Figure 1).

In summary, as a whole, the glycolytic pathway operates as a molecular information processing system, which at every moment redefines metabolic information flows that make each irreversible enzyme evolve with well-defined catalytic activities, under different inputs of glucose concentrations. The glycolytic metabolic subsystem behaves as a catalytic unit highly self-organized (dissipative processes) and self-regulated against the external changes of glucose input concentrations (molecular information processing). Each irreversible enzyme carries out three informative operations simultaneously: molecular information reception, molecular information integration, and source of new informative molecular flows. Thus, the dissipative multienzyme subsystems exhibit, at any time, precise sets of molecular instruction fluxes, and, as a result, each multienzyme complex shows specific and well-defined metabolic activities that permanently self-regulate the system. These dissipative multienzyme complexes can be considered the most fundamental units to accomplish the processing of molecular information in the cell (De la Fuente and Cortés, 2012).

### Self-Regulation by Molecular Information Processing in DMN

To understand how different multienzyme complexes functionally operate together, TE was applied for the study of the catalytic activities of DMNs (De la Fuente et al., 2011). In this work, a complex DMN of 18 metabolic subsystems was analyzed which is depicted in Figure 2. Each catalytic unit represents a self-organized multienzyme set (MSb). Figures 3A,B shows the structural organization of substrate fluxes, two classes of allosteric processes (activatory and inhibitory signals), and covalently modifying activities. DMNs are open systems, and S1 and S2 represent substrate external sources to MSb3 and MSb10, and MSb6 activity ends outside the metabolic network.

First, the dynamics of that network were studied with two inputs of substrate sources, S1 and S2. The spontaneous emergence of a *SMS* was observed under these external conditions, i.e., MSb12 was permanently active working as the metabolic core, the other subsystems presented on-off shifting states, and MSb15 was inactive (Figures 2A,B). All active self-organized enzymatic subsystems presented complex catalytic behaviors with different steady and oscillatory patterns. For instance, in Figures 2C,D, some dissipative catalytic activities of the MSb12 are showed. Specifically, this dissipative multienzyme set exhibited activity cycles with more than 350 transitions between different spontaneous periodic oscillations and steady-states.

**FIGURE 4:**
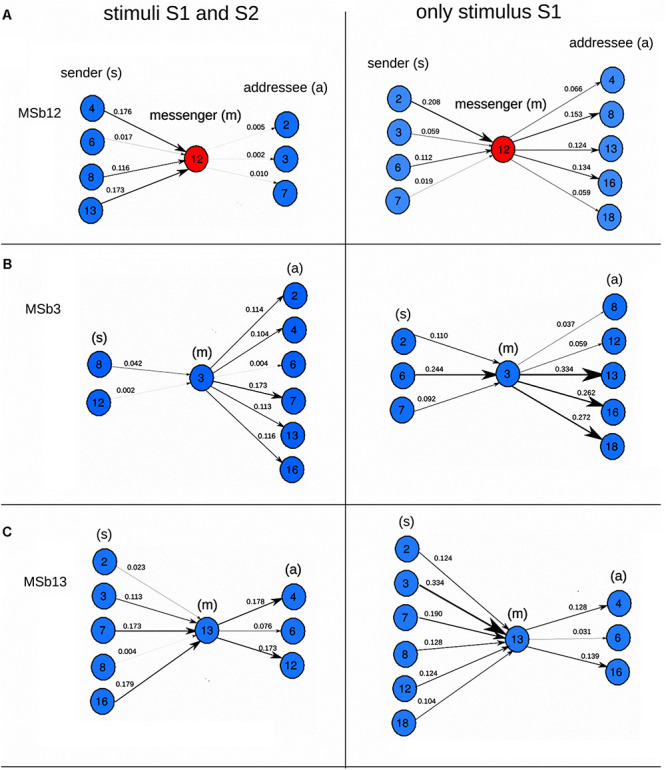
Molecular information processing in a dissipative metabolic network. In-ward and out-ward of molecular information flow between self-organized multienzyme sets. The in-ward and out-ward transfer entropy of three dissipative multi enzymatic complexes MSb12 **(A)**, MSb3 **(B)**, and MSb13 **(C)** are plotted for two conditions (two external inputs of substrate sources S1–S2 in the left panels, and one external substrate source S1 in the right panels). Self-organized multienzyme sets are represented in blue and black circles, MSb12 metabolic core is in red. This figure is an explanatory extension of the results shown in Figures 3E,F. It can be observed that the network (Figure 2) is permanently self-regulated through molecular information processing, displaying significant variations of information fluxes. The Dissipative Network redefines sets of biochemical instructions at each moment that makes all multi enzymatic complexes evolve with particular and precise activity patterns. The network system behaves as a catalytic unit highly *self-organized* (dissipative patterns) and *self-regulated* (molecular information processing). The units are in information bits (Log function in the Shannon information computed on base 2), and all bit values were normalized to the maximum. Letters (s), (m), and (a) refer to sender, messenger, and addressee. Part of this figure has been reported previously by De la Fuente et al. (2011).

**FIGURE 5:**
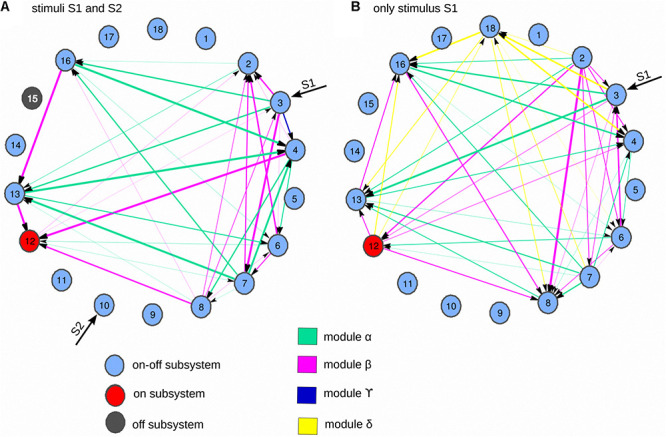
Modularity in the dissipative metabolic network. As a result of self-regulated dynamics, through molecular information processing, systemic metabolic modules between different self-organized multienzyme sets emerge in the network. This figure is an explanatory extension of the results shown in Figures 3E,F. **(A)** Two external inputs of substrate sources S1 and S2 are presented to the network, **(B)** only an external stimulus S1. The TE connectivity preserved under the two external conditions is depicted in green (module alpha). The informative flows but with inverted directionality that occurs under the two external conditions are plotted in pink (module beta). The informative connectivity that only appears in condition **(A)**, but not in panel **(B)** is showed in dark-blue (module gamma), and the connections in **(B)** but not in panel **(A)** are represented in yellow (module delta). The modular transitions at the systemic level originate metabolic switches that enable critical changes in the catalytic reactions. Part of this figure has been reported previously by De la Fuente et al. (2011).

Removing S2 and keeping only S1, as the external source of the substrate, a different functional reorganization emerged in the network (Figures 3D–F, 4, 5). However, the *SMS* was maintained (the same metabolic core MSb12, while the rest of the subsystem, including the MSb15, was eventually active with on-off dynamics). Even more, the network adapted its metabolic activity to the new environmental situation (a single stimulus) through its structural and flux plasticity (Almaas et al., 2005: De la Fuente, 2015).

The TE of the biochemical network was evaluated by analyzing the amplitude of metabolic patterns (Figures 2E,F). There were just nine dissipative multienzyme sets with significant TE values when two concurrent stimuli were studied (Figure 3E). In this picture, the thickness of the lines was proportionate to the TE values, whilst the directions of arrows indicated the directionality of the effective connectivity. The highest values of molecular information corresponding to the connection between MSb13 and MSb16 were also observed under two stimuli (Figure 3E). The analysis of TE, when only a source of external substrate S1 was maintained is depicted in Figure 3F. These results indicated that the molecular information structure was far more complex: 1° effective connections were found in 10 multienzyme subsystems; 2° the density of informative connections was incremented in the metabolic network (from 10.802 to 13.580); 3° the strength of links was also incremented (from 0.079 to 0.133 in average). 4° the maximum TE value was 0.377 (one stimulus) against 0.179 bits (two stimuli). Quantitative and qualitative alterations of TE values were evident in the metabolic core (see some examples in Figure 4). In particular, it can be observed that the core gets a molecular information signal from MSb8 under two stimuli (with a TE of 0.116, Figure 4A, on the left). However, the TE flow direction is reversed, and the flux of molecular information is sent to MSb8 by the core, under one stimulus (with a TE of 0.153, see Figure 4A, on the right).

According to the TE analysis, it can be observed that, in addition to the molecular network’s structure (Figures 3A,B), a systemic functional structure formed by molecular information fluxes emerges in the network (Figures 3E,F, 5A,B). This *SMS* defines a complex effective information system that integrates every catalytic activity in a global metabolic entity. Such systemic informative structure is formed by modules, and the dynamic transitions between them shape metabolic switches that enable essential functional changes in the network (Figure 5). Therefore, the functional switches and the modules of effective connectivity are also critical elements of the *SMS*.

In summary, a complex structure of information processing emerges in the dissipative metabolic system at the global level, similar to parallel computing. “The dissipative network behaves as a highly complex decentralized information processing super-system that generates molecular information flows between the self-organized enzymatic sets, forcing them to be functionally interlocked, i.e., each catalytic set is conditioned to cooperate with others at a specific and precise activity regime in concordance with the activity. According to the overall functional process, the network defines sets of biochemical instructions at each moment that make every metabolic subsystem evolve with precise and particular dynamic catalytic patterns” (De la Fuente, 2015). In short, the network is capable of self-organizing far from equilibrium through dissipative processes (exhibiting complex quasi-stationary and oscillatory catalytic patterns) and self-regulating versus external perturbations through molecular information processing, adapting dissipative metabolite patterns to alterations coming from outside the system. Besides, the information generated in these processes highly increases the complexity of cellular system (De la Fuente et al., 2010, 2011).

One of the best-known examples of information processing at the cellular level is the *E. coli* chemotaxis network. This is one of the most studied metabolic complexes able to store biochemical patterns by processing molecular information (see Supplementary Material 02 for more details). On the other hand, information processing at a molecular level has been observed in numerous biochemical systems (see Supplementary Material 02).

Despite all investigations, many aspects of molecular information processing remain to be elucidated.

## Hopfield-Type Systemic Attractors in DMN. Cellular Systemic Behaviors

**FIGURE 6:**
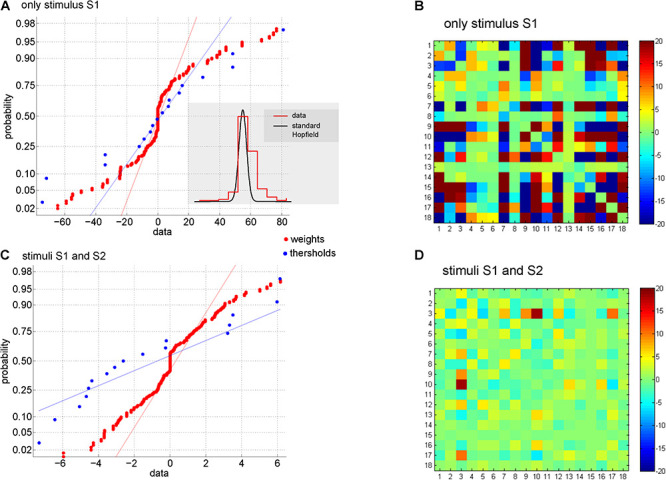
Equivalent Hopfield network through a Boltzmann machine in dissipative metabolic networks. In both conditions analyzed: **(A)** only an external source of biochemical substrate S1, **(B)** two external inputs of substrate sources S1 and S2, the weights, depicted in red, and the thresholds, plotted in blue, show a strongly non-Gaussian distribution. **(C,D)** Two matrices of weights connectivity obtained under two external conditions are presented; they were learned from the dissipated metabolic network (Figure 2) by the Boltzmann machine. Part of this figure has been reported previously by De la Fuente et al. (2013).

**FIGURE 7:**
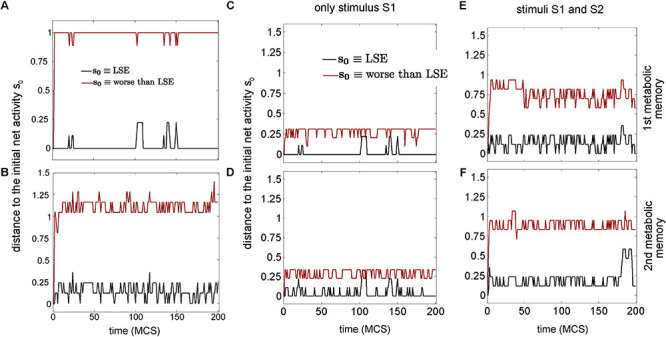
Metabolic associative memories in dissipative metabolic networks. **(A,B)** One metabolic memory encoded in the weights. In black are depicted some dissipative metabolic dynamics when the initial activity is the same as the dynamic memory given by the LSE solution (Least Square Error); this biochemical dynamic memory is a local minimum of the equivalent Hopfield network obtained from the dissipative network (Figure 2), a proof of associative memory. An arbitrary solution is also shown in red which is of poorer quality than the obtained by LSE. **(C–F)** In black, two metabolic memories encoded in the weights under only external input source of substrate S1 and both external inputs of substrate sources S1 and S2 are plotted. The first metabolic memory is shown in panels **(C,E)**, and the second metabolic memory is displayed in panels **(D–F)**. Both two metabolic memories are local minima, which does not occur with an arbitrary solution plotted in red. Thus, the equivalent Hopfield network of the dissipative metabolic system can exhibit associative memory. Times are in Monte Carlo Steps’ units (MCS). Fluctuations were established by a temperature parameter of *T* = 0.7. The systemic enzymatic activities of the Dissipative Metabolic Network (Figure 2) are driven by Hopfield-like attractors with a capacity to store catalytic behaviors which can be retrieved appropriately from determining external inputs of substrate sources. These systemic attractors govern the metabolic activities, change the functional connectivity between the dissipative self-organized multienzyme sets, and stably keep these changes. Part of this figure has been reported previously by De la Fuente et al. (2013).

“Memory is a fundamental requirement for efficient information processing” (De la Fuente, 2015). In this regard, Hopfield-like dynamics with memory were verified in DMN in 2013 (De la Fuente et al., 2013). In such a study, global information and energy of the emerging biochemical dynamics in DMN (Figure 2) were studied by using methods of Artificial Intelligence and Statistic Mechanics. In addition to the macroscopic properties analyzed, Hopfield-like behaviors were obtained using a Boltzmann machine (Figures 6, 7). As it is well known, these Hopfield-like dynamics are characterized by manifesting associative memory (Hopfield, 1982).

Specifically, the preliminary analyses of the DMN showed spontaneous self-organization of all enzymatic patterns which lead to the emergence of a *SMS* characterized by a metabolic core formed by always active enzymatic processes, while the others are eventually active. Also, the dissipative metabolic network was able to self-adjust the internal enzymatic activities according to the external changes by self-regulatory processes (molecular information processing), exhibiting both metabolic structural plasticity (persistent alterations in the state of multi enzymatic subsystems, switches between active, inactive, and varying on-off states) and metabolic flux plasticity (changes in metabolic synapse values leading to a differential catalytic activity); this type of global self-regulation has been observed in several unicellular organisms (Almaas et al., 2004; Almaas, 2007). The quantitative study of the DMN showed the following main results:

First, the Lyapunov function of the biochemical system was calculated. Starting in any initial state under a specific external stimulus, the enzymatic dynamics of the system evolve intending to decrease the energy function. In noise absence, this function will monotonously decrease until it reaches a terminal metabolic state (local minimum corresponding to a stable state of the DMN). There are multiple functional stable states for a DMN.

Second, due to self-organized and self-regulated processes in the DMN, systemic attractors (locally stable states) emerge and globally govern the network. All enzymatic dynamics are stabilized in these systemic attractors. Multiple attractors (and therefore multiple local minima) define the complex landscape on which the systemic enzymatic dynamics rely. As a consequence of the attractor dynamics, the metabolic system works as a fully integrated and individual entity.

Third, “the systemic attractors that emerge in the system correspond to Hopfield-like dynamics which can store metabolic information patterns (Hopfield, 1982). The biochemical information contained in the attractors behaves as a functional metabolic memory, i.e., enzymatic activity patterns stored as stable states, regulates both the permanent catalytic changes in the systemic network and the metabolic responses originated to integrate the perturbations coming from the external environment properly. When an external stimulus pattern is presented to the system, the network states are driven by the intrinsic enzymatic dynamics toward a determined systemic attractor which corresponds to a set of memorized biochemical patterns” (De la Fuente, 2015). As it is generally known, such “pattern-completion” dynamics have long been studied in Hopfield networks, and they are usually referred to as “associative memory” (Hopfield, 1982; Hertz et al., 1991; Amit, 1992; Peretto, 1992).

In sort, Hopfield-like metabolic attractors store systemic enzymatic dynamics which may be properly recovered from external stimuli. Metabolic dynamics are systemically governed by these metabolic attractors which modulate the enzymatic activities, change the connections among dissipative multienzyme associations, and stably store these dynamic connections.

The study with Hopfield networks (De la Fuente et al., 2013) quantitatively evidenced, for the first time, the possibility that a cell can have associative memory. Such Hopfield-like memory would be a cellular type of epigenetic memory (De la Fuente, 2015).

### Verification of Associative Memory in Unicellular Organisms

The principles of associative memory were discovered at the beginning of the 20th century by Nobel Laureate Ivan Pavlov in his classic studies with dogs, which opened up a new paradigm in behavioral sciences.

In all organisms with a nervous system, associative learning and memory are essential cognitive characteristics that allow obtaining critical information for adaptation and survival, mastering new behaviors through the association of diverse stimuli. These characteristics are ubiquitous in numerous species from mollusks to humans, but until now they have never been observed in individual cells.

To experimentally verify the emergence of associative memory in cells, the systemic motility patterns of two eukaryotic microorganisms such as *A. proteus* and *M. leningradensis* were analyzed under an associative conditioning setting (De la Fuente et al., 2019a). For this purpose, two stimuli were tested following Pavlov’s concept of experiments, a suitable direct-current (DC) electric field, and an N-formylated tripeptide linked to amoeba nutrition.

**FIGURE 8:**
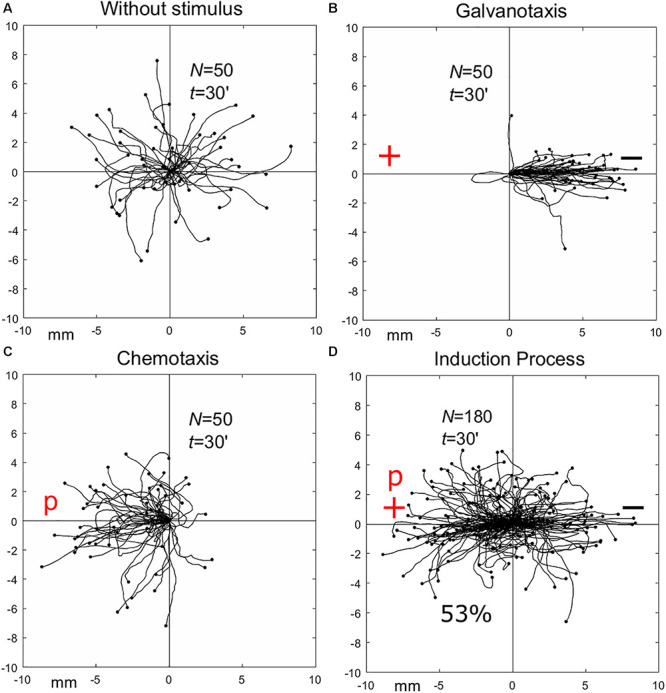
Migration trajectories of *Amoebae proteus* under four basic and independent experimental conditions. **(A)** In the absence of stimuli, amoebae showed *random* motion in all directions from the central point where they were placed. **(B)** In the DC electric field, all cells moved to the negative pole (galvanotaxis). **(C)** In the chemotaxis assay, 86% of the amoebae exhibited robust directionality toward the peptide location. **(D)** In the induction process (simultaneous galvanotaxis and chemotaxis), 53% of cells migrated toward the anode-peptide. “N” number of cells tested, “t” duration of the experiment. “p” nFML chemotactic tripeptide, “+” anode, “-” cathode. In panels **(A–D)**, the distance in both axes is in mm. The initial location of each cell was at the center of the diagram. Part of this figure has been reported previously by De la Fuente et al. (2019a).

**FIGURE 9:**
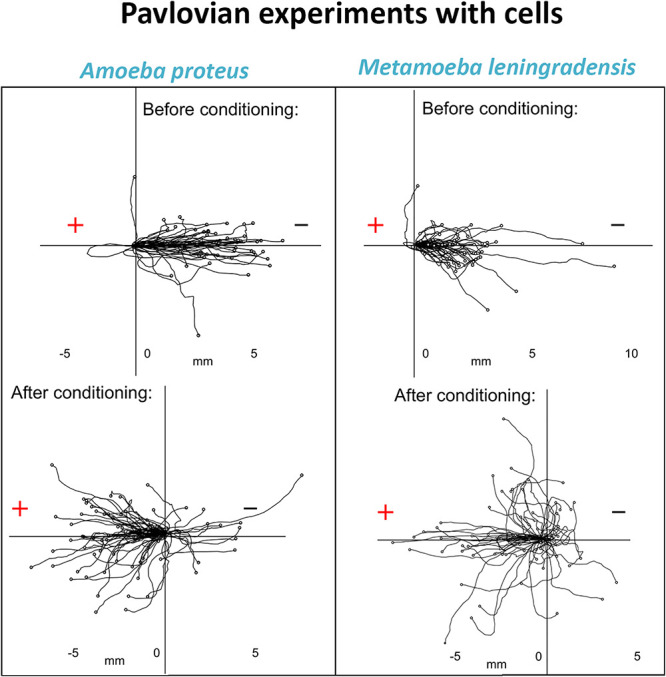
Experimental evidence of associative memory in cells. In the galvanotaxis, almost all cells of two species generally move to the cathode. After the conditioning process (simultaneous application of both galvanotactic and chemotactic stimuli), the cells, which showed migration to the anode, were tested in another galvanotaxis; most of them persisted in motion toward the anode in the absence of peptide. Thus, these cells revealed the unknown ability to acquire a new behavior and memorize it for an average of 40 min, a relatively long time compared to their life cycle. Part of this figure has been reported previously by De la Fuente et al. (2019a).

The most important aspects of this work on cellular associative conditioning are summarized below:

- First, it was found that in the absence of stimuli cellular locomotion directionality displayed a random distribution when amoebae and metamoebae probed almost all the directions of the experimental chamber (Figure 8A).

- In the next experimental step, galvanotaxis, amoebae, and metamoebae exhibited an unambiguous systemic reaction consisting of movement to the negative pole when subjected to a strong DC electric field of about 300–600 mV/mm (Figure 8B).
- In the third step, the response of both unicellular organisms was studied during their exposure to a gradient of nFMLP, distributed in the left part of the experimental chamber (chemotaxis, Figure 8C). In these circumstances, most cells showed a stochastic migration with robust directionality toward the peptide.
- In the fourth step, an induction process was performed. To this end, both stimuli, galvanotactic and chemotactic, were applied to amoebae simultaneously for 30 min. The nFMLP peptide solution was added to the anode well, on the left part of the chamber (Figure 8D). In these experiments, only about half of the amoebae and metamoebae migrated to the cathode and the rest moved to the anode site, where the peptide was deposited.
- Finally, to check if the cells conserve their movement pattern to the anode-nFMLP, the cells that showed such migration in the fourth step were exposed once more for 30 min to a single electric field stimulus, omitting peptide at the anode. The individual cell trajectories analyzed after the experiments revealed the migration of most of these cells to the anode in the absence of peptide (Figure 9), so it was shown that after the induction process, amoebae and metamoebae developed a new pattern of locomotion: moving to the anode under galvanotaxis conditions. It is worth remembering that almost all cells in the DC field move to the cathode (see Figure 8B).

Under the application of two simultaneous stimuli (induction process), the DC electric field and the peptide related to the nutrition of amoeba, placed at the positive pole, some amoebae seem to relate these stimuli (anode and peptide), and subsequent control tests showed that most of the conditioned *A. proteus* and *M. leningradensis* changed their systemic behavior, moving toward the anode where there was no nFMLP peptide.

They acquired a new persistent pattern of cellular motion, described by the move toward the anode (Figure 9), instead of their established propensity to run to the cathode. The conditioned amoebae were able to associate simultaneous but unrelated past events, producing persistent movement (contrary to their natural tendency), which on average could continue for 44 min. Besides, a vast number of strict controls proved the robustness and predictability of this behavior.

These experiments, in which a single cell links two different simultaneous stimuli thus developing a new behavior, are the first published evidence that an associative Pavlovian-like memory is present in single cells (De la Fuente et al., 2019a).

Cellular conditioning may be necessary for critical functions, for example, the regulation of migration. Locomotion movements are fundamental not only to survive and avoid predators, but also in individual cells of multicellular organisms to carry out essential life processes like embryogenesis, organogenesis, immune response, tissue repair, and others. Several severe health conditions, such as cancer, vascular and heart diseases, and intellectual disability, may develop as the consequences of inaccurate control of this systemic motility property. Such migratory cellular abilities are also present in the metastatic process of cancer (De la Fuente and López, 2020).

## Integrative Summary

In this review, we have mainly approached four aspects that characterize cellular functioning: the role of dynamic molecular processes, the mechanisms implicated in self-organization (dissipative structures) and the self-regulation of metabolic dynamics (molecular information processing), and the emergence of the cellular systemic properties.

More specifically, the work focuses on the main principles that govern enzymatic activity, under complex dynamic conditions prevailing inside the cell, which are crucial to elucidate the structural and functional architecture of basic units of life.

The scope of Systems Biology applied here is based on integrating concepts and knowledge in Biochemistry, Enzymology, Molecular and Cell Biology, and other biological sciences. We also discussed the results of quantitative research, analyzed by application of physical-mathematical tools such as statistical mechanics, differential calculus, discrete mathematics, artificial intelligence, computing, and others. Nevertheless, favoring the didactic purpose of the review we did not use the mathematical formulations.

The following paragraphs add some conclusive examples and summarize the main aspects of this review in an integrative way.

### Cellular Molecular Dynamics

Strictly speaking from a biochemical viewpoint, the cell is a complex molecular reactor, extremely self-organized in a sophisticated manner, and in a permanent biomolecular recycling status (section “The Dynamics Originated by the Molecular Turnover and the Role of the Enzymes in These Super Complex Dynamics”).

Millions of biochemical reactions happen simultaneously in every basic unit of life at any time. This process of reactive transformations also includes subcellular structures. Nothing is inert, all the molecules and structures that make up each cell undergo complex chemical transformations (see Supplementary Material 01). Notice that, despite the efforts, many aspects of cellular recycling remain poorly understood by molecular experimentalists.

The cell is an extremely complex and dynamic system; the endless molecular turnover, which characterizes it, modifies the chemical composition of the whole reactor every second and, as a consequence, the concentration patterns of ions and molecules change permanently over time. These continuous cycles of synthesis and destruction, and incessant chemical molecular transformations shape the essential scenario in which cellular life is possible. When this dynamic process of molecular turnover collapses, the cell dies. This is the main systemic characteristic of all living unicellular organisms.

The consequences of self-regulated turnover dynamics are adequate cellular growth, the development of all physiological processes to maintain its functional structures, continuous adaptation to the environment, and mitosis.

The highly complex organization of cellular turnover dynamics defies the human intellect. Any attempt to synthetically reproduce this global molecular turnover, either *in vitro* or *in silico*, has failed so far. In every cell, molecular synthesis and destruction are compensated and harmonized between them, following complex and unrepeatable reactive patterns, whose laws and defining principles are still unknown.

### Enzymes, Essential Cellular Molecules

Enzymes are fundamental molecules for metabolic life. Molecular reactive transformations are essentially chemical reactions, modifications on how atoms are bonded. Practically, all reactive transformations in the cell are mediated by enzymes, extraordinary macromolecular nanomachines responsible for breaking and joining covalent bonds, the fundamental chemical links in biological molecules (section “The Dynamics Originated by the Molecular Turnover and the Role of the Enzymes in These Super Complex Dynamics”). Cellular life would be impossible without enzymes; they are key protagonists in all reactive and physiological processes in the cell.

Unlike other cellular molecules, enzymes are *active* macromolecules responsible for the chemical work. Through their ability to decrease the activation energy of bonds, enzymes permanently modify how atoms are bonded, making possible the accelerated creation and destruction of molecules.

Catalytic enzymatic activities are termed metabolic processes. There are two basic types of enzymatic reactions, catabolism (implicated in molecule destruction) and anabolism (implicated in molecule synthesis). Together, these enzymatic activities are called cellular metabolism. In short, the cell is a complex enzyme-mediated metabolic system where reactive molecular transformations of synthesis and destruction happen unceasingly mediated by catalytic activities.

### Dissipative Self-Organization, the Primary Source of Molecular Organization in the Cell

Reactive cellular processes have little to do with the Chemistry of Equilibrium. The fundamental characteristic of cellular biochemistry is that the catalytic reactions, considered globally, occurs far from the Thermodynamic Equilibrium. Under these particular conditions, irreversible enzymatic processes raise the spontaneous emergence of self-organized dissipative structures (section “Dissipative Self-Organization of Enzymatic Activities, the Main Source of Molecular Organization in the Cell”).

Different catalytic mechanisms allow processes far from Thermodynamic Equilibrium, and one of the most important is the non-linear enzyme kinetics of many irreversible enzymes with allosteric regulation (Goldbeter, 2002, 2007).

Two principal types of self-organized dissipative structures emerge in the cell, temporal molecular rhythms, and spatial waves. In temporal rhythms, molecular concentrations fluctuate over time in an oscillatory manner (Figure 1). Every cell appears as a dynamic system in which cellular self-destruction and self-construction happen following complex quasi-stationary patterns and unrepeatable molecular rhythms. On the other hand, the dissipative spatial molecular waves modulate the cellular metabolic activities, synchronizing and regulating functionally different enzymatic processes. All these self-organized behaviors are unlikely for the Chemistry of the Thermodynamic Equilibrium (De la Fuente, 2010).

So, the cell is a metabolic system in which self-construction and self-destruction occur following complex catalytic rhythms, mainly coordinated by spatial molecular waves.

The self-organized dissipative structures were discovered by Nobel Laureate Ilya Prigogine (Nicolis and Prigogine, 1977), and these dynamic processes constitute the primary source of molecular order in the cell. The concept of dissipative order is a fundamental issue to describe the molecular-functional architecture of living cells. Dissipative self-assembling and dissipative self-organization are the two pillars of the molecular order and the emergent functional complexity of cells (De la Fuente, 2015). However, being biologically essential, many issues of cellular dissipative structures remain unclear, which warrant further investigation.

### Cellular Enzyme Organization. *Cellular Metabolic Structure*

At the cellular level, enzymatic activity dramatically depends on the collective functional structure of catalytic processes which is defined by the two fundamental principles that connect the main forms of enzyme organization in the cell: *metabolic segregation* and *systemi*c *metabolic integration*, operating simultaneously (section “Dissipative Metabolic Networks and the Emergence of the Systemic Metabolic Structure”).

Enzymes do not work independently of each other in the cellular molecular crowding, but instead shape different types of multienzyme associations, grouped by their metabolic tasks and catalytic roles. Thus all chemical reactions that occur in living cells are segregated functionally (De la Fuente, 2015).

Moreover, these catalytic complexes interact reversibly with structural proteins and membranes originating thermodynamically open microcompartments, in which molecular rhythms can regulate the efficiency of the enzymatic reactions involved (De la Fuente, 2010).

The term *Metabolic Subsystem* was suggested in 1999 to refer to these self-organized multienzyme sets in which complex quasi-steady-state behavior and molecular oscillations may emerge spontaneously inside the cell (De la Fuente et al., 1999b). These dissipative catalytic complexes (Figure 1) represent highly efficient nanostructures capable to perform autonomous biochemical works which constitute basic catalytic units of the cellular organisms (De la Fuente, 2015).

Multienzyme complexes are organized into distinct modular networks with specific and coherent autonomous activities. At a superior level, the metabolic networks (Figure 2) appear to be integrated (*systemic metabolic integration*) forming a very complex dynamic super-system, the *CMS*. Such global structure was observed for the first time in 1999 by analyzing DMN through numerical studies (De la Fuente et al., 1999b, 2008) and was later corroborated by flux balance analysis in prokaryotic and eukaryotic cells (Almaas et al., 2004, 2005; Almaas, 2007; De la Fuente, 2015).

### Information Processing. The Second Source of Molecular Order in the Cell

A large number of experimental studies have shown that unicellular organisms possess complex self-regulatory behavior originated by biomolecular information processing, another fundamental source of organization in the cell (section “Enzymatic Information Processing, the Second Leading Source of Molecular Order in the Cell”).

The dissipative multienzyme complexes permanently send information through metabolite patterns between their different enzyme components. As a result of the overall process, these enzymatic assemblies operate as basic molecular information processing units (Figure 1). Thus, each dissipative multienzyme complex defines, at any time, precise sets of molecular instruction fluxes, and as a result, each multienzyme complex exhibits well-defined and specific metabolic activities (De la Fuente and Cortés, 2012).

On the other hand, a complex parallel super-system of information processing appears in the metabolic networks when the systemic activity is considered (Figure 2). *CMS* behaves as a self-regulated dynamic entity that permanently sends specific regulatory signals to multi-enzyme complexes make them evolve in well-defined and precise metabolic activities by information processing, see Figures 3–5 (De la Fuente et al., 2010, 2011, 2013; De la Fuente, 2015).

Molecular information processing allows the formation in the cellular metabolism of a network of functional links that readjust the catalytic patterns, adapting them to the physiological needs of cells during environmental changes (self-regulation process). Besides, the information generated in these processes highly increases the complexity of cellular system. *Physarum polycephalum* is a paradigmatic example of the emergence of complex cellular behaviors mediated by information processing in unicellular organisms. This microorganism can discover the minimum-length option between two distant points in a labyrinth (Nakagaki et al., 2000; Nakagaki, 2001). Note that finding the shortest path problem in a maze needs a rigorous mathematical analysis (Miyaji and Ohnishi, 2008). *P. polycephalum* is also capable of improving the selection of the better route configuration obtained by the shortest Steiner’s minimum tree connections, thus developing adapted strategies to maximize its access to nutrients (Nakagaki et al., 2004a; 2004b). Even more, *P. polycephalum* achieves to solve difficult problems, for example, finding a high-quality solution to the problem of the traveling salesman, a question which is known to be NP-hard (Aono et al., 2011a, b; Zhu et al., 2011). This multinucleated amoeba is capable of designing an optimal network very similar to the purpose-intended system in the Tokyo railway organization (Tero et al., 2010). During the process of adaptation to different inputs, *P. polycephalum* succeeds memorizing changes in its environment, recalling them later to adapt its behavior to the new conditions appropriately, for example, anticipating a cold-dry pattern in the environment 1 h before the change happens (Saigusa et al., 2008). It has also been observed that this protist accomplishes complex dilemmas of multi-objective foraging (Bonner, 2010; Dussutour et al., 2010; Latty and Beekman, 2011). Recently it has been shown that *P. polycephalum* is capable of developing a kind of rudimentary learning (Boisseau et al., 2016). Molecular information processing has been observed in numerous other biochemical systems (see Supplementary Material 02).

### Hopfield-Like Dynamics and Associative Memory

Efficient information processing is impossible without memory (section “Hopfield-Type Systemic Attractors in Dissipative Metabolic Networks. Cellular Systemic Behaviors”). The presence of Hopfield-like dynamics characterized by associative memory in dissipative enzymatic networks was numerically verified in 2013 (De la Fuente et al., 2013). Such a memory would be a manifestation of emergent properties underlying the complex dynamics of the systemic cellular metabolic networks when dissipative enzymatic self-organization and molecular information processing act together. This study was the first quantitative evidence that an individual cell can use associative memory, which is supported by epigenetic mechanisms (De la Fuente et al., 2013; De la Fuente, 2015). As a consequence of the emergent Hopfield-like attractors, the systemic metabolism works as a fully integrated and individual entity (Figures 6, 7).

Associative memory enables all organisms with a developed nervous system, from mollusks to humans, to learn and adapt successfully to specific environmental stimuli (Pavlov, 1927; Mackintosh, 1983), but until now this type of learning has never been described in unicellular organisms. Very recently, patterns of locomotion consistent with an associative conditioned behavior have been observed in two microorganisms, *A. proteus* and *M. leningradensis* (De la Fuente et al., 2019a). Both amoebae species were capable of linking two simultaneous but unrelated past events, demonstrating a new migration behavior that prevailed for a relatively long time, *44 minutes on average* (Figures 8, 9). This finding confirmed in 2019 the prediction from the previous studies of 2013 on the emergence of Hopfield-like attractors with associative memory at the systemic cellular level (De la Fuente et al., 2013).

Along with the investigation of associative conditioning in single cells, a quantitative study on the dynamic characteristics of the systemic motility of *A. proteus* was carried out recently through advanced analysis of locomotion movements in intact and enucleated (cytoplasts) amoebae. This research also allowed the quantification of the importance of the nucleus during the cellular migration (De la Fuente et al., 2019b). Such study calculated specifically the movement fluctuations along the locomotion trajectories of the amoebae utilizing the “rmsf” method, a known technique in Statistical Mechanics based on a work by Einstein (1905), later elaborated and widely used to analyze time-series. The research revealed that individual cells without nuclei (cytoplasts) and intact cells displayed a similar migratory structure mainly described by non-trivial long-range correlations. “This dynamic memory (non-trivial correlations) represents a key characteristic of *A. proteus* movements during cell migration. It is worth noting that this temporal persistence (correlations with 41.5 min on average) in cells and cytoplasts matches with the Pavlovian-like dynamic memory (44 min on average)” (De la Fuente et al., 2019a).

The nucleus has been classically considered an essential element governing cell movement, nevertheless direct quantitative evidence was lacking. The migratory analysis of enucleated and non-enucleated amoebae described above (Bringas et al., 2017; De la Fuente et al., 2019b; De la Fuente and López, 2020) also demonstrated that the absence of nucleus does not impact the systemic movements of amoebae significantly. An independent group has reached a similar conclusion using other experimental methods in human cells (Graham et al., 2018).

To summarize, between the extracellular space and the DNA a dissipative self-organized *CMS* exists (De la Fuente, 2015). This super-complex dynamic structure behaves as a decentralized information processing system, “generating sets of biochemical instructions that drive each enzymatic activity to a particular and precise dynamic of change, allowing self-regulation and adaptation to the external medium. CMS permanently sends a flow of molecular signals to the DNA-associated metabolism, thus contributing to forming the complex transcriptional system. These molecular flows allow the accurate regulation of gene expression, so that only the specific polypeptides necessary for the adaptive maintenance of the CMS are synthesized. Altogether, both informative systems (CMS and DNA) coordinate the physiological development of the cell” (De la Fuente, 2015).

An extensive study performed with DMN (around 15,210,000), to research the mechanism behind the emergence of the *CMS*, allowed us to observe *that this global superstructure is a property common to all SMSs with an high number of dissipative self-organized multienzyme complexes* (De la Fuente et al., 2009). The fundamental factor that ensures the spontaneous emergence of CMS is a high multiplicity of dissipative metabolic sets inside the cell.

The high redundancy of the enzymes (with an increased number of copies) and the consequent multiplicity of dissipative self-organized multienzyme complexes that prevail inside the cell would determine the spontaneous formation of the SMS.

A continuous process of molecular synthesis and destruction represents thousands of metabolic reactions that happen permanently and simultaneously in the cell, ensuring the emergence, robustness, and stability of the *CMS*.

The only possible scenario for cellular life is a dynamic system in permanent self-construction and self-destruction process that guarantee the functionality of a high number of dissipative self-organized multienzyme complexes. There is no other alternative. This sophisticated and massive dynamics of endless molecular transformations encompasses the whole metabolic system and constitutes the most critical dynamic property, essential and definitory, of cellular life.

The word “metabolism” comes from the Greek *metabole* meaning “change” or “transformation.” This word reflects exactly the essential characteristic of the basic units of life. Rather than a molecular genotheque (box of genes) in evolution, the cell could be considered a singular dynamic metabolic unit capable to self-organize and self-regulate in an endless process of molecular turnover.

“Metabolism can be considered the largest known source of molecular complexity in nature” (De la Fuente, 2015). Roughly 3,700 million years ago (Dodd et al., 2017), an exceptional and singular metabolic dynamic emerged from primeval matter, characterized by a high structural and functional order, improbable for the Chemistry of Equilibrium. This extraordinary molecular organization perpetuates itself by direct transmission after every mitosis, and there is no scientific proof that another parallel metabolic organization has emerged “*de novo.*”

Across the millennia, many adaptive molecular mechanisms have been acquired in the biological evolution by the *CMS*. “Through the structural and functional complexities, the metabolic evolution has developed a large diversity of biochemical organizational forms, ranging from sophisticated bacterial metabolism, via the complex enzymatic networks, characteristic of protists, to the extraordinary structures and processes derived from the multicellular eukaryotic organization, such as embryogenesis or the neural networks present in higher mammals. The millions of living metabolic species represent the inexhaustible source of complexity developed by the dynamic forces of the cellular metabolism” (De la Fuente, 2015).

Every unicellular organism results from the direct and uninterrupted transmission of this extraordinary and singular metabolic organization. Over millions of years, sophisticated metabolic dynamics have evolved to originate complex biological supracellular structures, reproducing at different scales the singular intrinsic organization enclosed in itself … up to become self-aware.

## Author Contributions

ID, LM, MF, JC-P, JL, and IM performed the analysis and design of the research mapping. LM involved in main funding. All authors wrote the manuscript and agreed with its submission. IM conceived, designed, and directed the investigation.

## Conflict of Interest

The authors declare that the research was conducted in the absence of any commercial or financial relationships that could be construed as a potential conflict of interest.

## References

[B1] Agarwal K. P. (2019). A biophysical perspective on enzyme catalysis. *Biochemistry* 58(6): 438–449. 10.1021/acs.biochem.8b01004 30507164 PMC6386455

[B2] Allard J. Mogilner A. (2013). Traveling waves in actin dynamics and cell motility. *Curr. Opin. Cell. Biol.* 25 107–115. 10.1016/j.ceb.2012.08.012 22985541 PMC3542403

[B3] Almaas E. Kovacs B. Vicsek T. Oltvai Z. N. Barabási A. L. (2004). Global organization of metabolic fluxes in the bacterium *Escherichia coli*. *Nature* 427 839–843. 10.1038/nature02289 14985762

[B4] Almaas E. Oltvai Z. N. Barabási A. L. (2005). The activity reaction core and plasticity of metabolic networks. *PLoS Comput. Biol.* 1:e68. 10.1371/journal.pcbi.0010068 16362071 PMC1314881

[B5] Almaas E. (2007). Biological impacts and context of network theory. *J. Exp. Biol.* 210(Pt. 9) 1548–1558. 10.1242/jeb.003731 17449819

[B6] Almeira N. Gusman S. R. (2017). Role of transcriptional bursts in cellular oscillations. *J. Theor. Biol.* 7 49–56. 10.1016/j.jtbi.2017.05.029 28549618

[B7] Amit D. J. (1992). *Modeling Brain Function: The World of Attractor Neural Networks*. Cambridge: Cambridge University Press.

[B8] Aon M. A. Cortassa S. O’Rourke B. (2006). The fundamental organization of cardiac mitochondria as a network of coupled oscillators. *Biophys. J.* 91 4317–4327. 10.1529/biophysj.106.087817 16980364 PMC1635654

[B9] Aon M. A. Roussel M. R. Cortassa S. O’Rourke B. Murray D. B. Beckmann M. (2008). The scale-free dynamics of eukaryotic cells. *PLoS One* 3:e3624. 10.1371/journal.pone.0003624 18982073 PMC2575856

[B10] Aono M. Zhu L. Hara M. (2011a). Amoeba-based neurocomputing for 8-city traveling salesman problem. *Int. J. Unconv. Comput.* 7 463–480. 10.1016/j.biosystems.2013.01.008 23438635

[B11] Aono M. Zhu L. Kim S. J. Hara M. (2011b). Performance enhancement of amoeba-based neurocomputer for 8-city traveling salesman problem. *Proc. NOLTA* A2L-B3, 104–107.

[B12] Araiza-Olivera D. Chiquete-Felix N. Rosas-Lemus M. Sampedro J. G. Peña A. Mujica A. (2013). A glycolytic metabolon in Saccharomyces cerevisiae is stabilized by F-actin. *FEBS J.* 280 3887–3905. 10.1111/febs.12387 23763840

[B13] Asano Y. Nagasaki A. Uyeda T. Q. P. (2008). Correlated waves of actin filaments and PIP3 in Dictyostelium cells. *Cell Mot. Cytosk.* 65 923–934. 10.1002/cm.20314 18814278

[B14] Atkinson D. E. Walton G. M. (1967). Adenosine triphosphate conservation in metabolic regulation. Rat liver citrate cleavage enzyme. *J. Biol. Chem.* 242 3239–3241. 6027798

[B15] Barril E. F. Potter A. R. (1968). Systematic oscillations of amino acid transport in liver from rats adapted to controlled feeding schedules. *J. Nutr.* 95 228–237. 10.1093/jn/132.11.3369 12421853

[B16] Bentele M. Lavrik I. Ulrich M. Stösser S. Heermann D. W. Kalthoff H. (2004). Mathematical modeling reveals threshold mechanism in CD95-induced apoptosis. *J. Cell Biol.* 166 839–851. 10.1083/jcb.200404158 15364960 PMC2172102

[B17] Bernardinelli Y. Magistretti P. J. Chatton J. Y. (2004). Astrocytes generate Na+-mediated metabolic waves. *Proc. Natl. Acad. Sci. U.S.A.* 101 14937–14942. 10.1073/pnas.0405315101 15466714 PMC522032

[B18] Berridge M. J. Galione A. (1988). Cytosolic calcium oscillators. *FASEB J.* 2 3074–3082. 10.1096/fasebj.2.15.2847949 2847949

[B19] Bertschinger N. Natschlager T. (2004). Real-time computation at the edge of chaos in recurrent neural networks. *Neural Comput.* 16 1413–1436. 10.1162/089976604323057443 15165396

[B20] Bier M. Teusink B. Kholodenko B. N. Westerhoff H. V. (1996). Control analysis of glycolytic oscillations. *Biophys. Chem.* 62 15–24. 8962468 10.1016/s0301-4622(96)02195-3

[B21] Boisseau R. P. Vogel D. Dussutour A. (2016). Habituation in non-neural organisms: evidence from slime moulds. *Proc. R. Soc. B.* 283 20160446. 10.1098/rspb.2016.0446 27122563 PMC4855389

[B22] Bonner J. T. (2010). Brainless behavior: a myxomycete chooses a balanced diet. *Proc. Natl. Acad. Sci. U.S.A.* 107 5267–5268. 10.1073/pnas.1000861107 20332217 PMC2851763

[B23] Boruc J. Van den Daele H. Hollunder J. Rombauts S. Mylle E. Hilson P. (2010). Functional modules in the *Arabidopsis* core cell cycle binary protein-protein interaction network. *Plant Cell* 22 1264–1280. 10.1105/tpc.109.073635 20407024 PMC2879739

[B24] Bringas C. Malaina I. Pérez-Samartín A. Boyano M. D. Fedetz M. Pérez-Yarza G. (2017). Long-term memory in the migration movements of enucleated *Amoeba proteus*. *BioRxiv* [Preprint] 10.1101/125054

[B25] Brodsky V. Y. (2006). Direct cell-cell communication: a new approach derived from recent data on the nature and self-organisation of ultradian (circahoralian) intracellular rhythms. *Biol. Rev. Camb. Philos. Soc.* 81 143–162. 10.1017/S1464793105006937 16336746

[B26] Buescher J. M. Liebermeister W. Jules M. Uhr M. Muntel J. Botella E. (2012). Global network reorganization during dynamic adaptations of *Bacillus subtilis* metabolism. *Science.* 335 1099–1103. 10.1126/science.1206871 22383848

[B27] Byrne K. M. Monsefi N. Dawson J. C. Degasperi A. Bukowski-Wills J.-C. Volinsky N. (2016). Bistability in the Rac1, PAK, and RhoA signaling network drives actin cytoskeleton dynamics and cell motility switches. *Cell Syst.* 2 38–48. 10.1016/j.cels.2016.01.003 27136688 PMC4802415

[B28] Carere J. Baker P. Seah S. Y. K. (2011). Investigating the molecular determinants for substrate channeling in BphI–BphJ, an aldolase–dehydrogenase complex from the polychlorinated biphenyls degradation pathway. *Biochemistry* 50 8407–8416. 10.1021/bi200960j 21838275

[B29] Carsten B. Karsten K. (2017). Intracellular oscillations and waves. *Annu. Rev. Condens. Matt. Phys.* 8 239–264. 10.1146/annurev-conmatphys-031016-025210

[B30] Castellana M. Wilson M. Z. Xu Y. Joshi P. Cristea I. M. Rabinowitz J. D. (2014). Enzyme clustering accelerates processing of intermediates through metabolic channeling. *Nat. Biotechnol.* 32 1011–1018. 10.1038/nbt.3018 25262299 PMC4666537

[B31] Causton H. C. (2020). Metabolic rhythms: a framework for coordinating cellular function. *Eur. J. Neurosci.* 51 1–12. 10.1111/ejn.14296 30548718

[B32] Chabot J. R. Pedraza J. M. Luitel P. van Oudenaarden A. (2007). Stochastic gene expression out-of-steady-state in the cyanobacterial circadian clock. *Nature* 450 1249–1252. 10.1038/nature06395 18097413

[B33] Chance B. Pye E. K. Ghosh A. D. Hess B. (eds) (1973). *Biological and Biochemical Oscillations.* New York, NY: Academic Press.

[B34] Cheng X. Ferrell J. E. Jr. (2018). Apoptosis propagates through the cytoplasm as trigger waves. *Science* 361 607–612. 10.1126/science.aah4065 30093599 PMC6263143

[B35] Chiam H. K. Rajagopal G. (2007). Oscillations in intracellular signaling cascades. *Phys. Rev. E.* 75(Pt. 1) 061901. 10.1103/PhysRevE.75.061901 17677294

[B36] Connor K. M. Gracey A. Y. (2011). Circadian cycles are the dominant transcriptional rhythm in the intertidal mussel *Mytilus californianus*. *Proc. Natl. Acad. Sci. U.S.A.* 108 16110–16115. 10.1073/pnas.1111076108 21911390 PMC3179051

[B37] Conrado R. J. Varner J. D. DeLisa M. P. (2008). Engineering the spatial organization of metabolic enzymes: mimicking nature’s synergy. *Curr. Opin. Biotechnol.* 19 492–499. 10.1016/j.copbio.2008.07.006 18725290

[B38] Dano S. Sorensen P. Hynne F. (1999). Sustained oscillations in living cells. *Nature.* 402 320–322. 10.1038/46329 10580506

[B39] De Forest M. Wheeler C. J. (1999). Coherent oscillations in membrane potential synchronize impulse bursts in central olfactory neurons of the crayfish. *J. Neurophysiol.* 81 1231–1241. 10.1152/jn.1999.81.3.1231 10085350

[B40] De la Fuente I. M. Martínez L. Veguillas J. (1995). Dynamic behavior in glycolytic oscillations with phase shifts. *Biosystems* 35 1–13. 7772719 10.1016/0303-2647(94)01473-k

[B41] De la Fuente I. M. Martínez L. Veguillas J. Aguirregabiria J. M. (1996a) Intermittency Route to Chaos. Quasiperiodicity route to chaos in a biochemical system. *Biophys. J* 71 2375–2379. 10.1016/S0006-3495(96)79431-6 8913578 PMC1233727

[B42] De la Fuente I. M. Martínez L. Veguillas J. (1996b). Intermittency route to chaos in a biochemical system. *Biosystems* 39 87–92. 8866045 10.1016/0303-2647(95)01603-1

[B43] De la Fuente I. M. Martínez L. Aguirregabiria J. M. Veguillas J. (1998a). R/S analysis in strange attractors. *Fractals* 6 95–100.

[B44] De la Fuente I. M. Martínez L. Benitez N. Veguillas J. Aguirregabiria J. M. (1998b). Persistent behavior in a phase-shift sequence of periodical biochemical oscillations. *Bull. Math. Biol.* 60 689–702.

[B45] De la Fuente I. M. Martínez L. Veguillas J. Aguirregabiria J. M. (1998c). Coexistence of multiple periodic and chaotic regimes in biochemical oscillations. *Acta Biotheor.* 46 37–51. 9558751 10.1023/a:1000899820111

[B46] De la Fuente I. M. Benítez N. Santamaría A. Veguillas J. Aguirregabiria J. M. (1999b). Persistence in metabolic nets. *Bull. Mathemat. Biol* 61 573–595. 10.1006/bulm.1999.0103 17883232

[B47] De la Fuente I. M. (1999). Diversity of temporal self-organized behaviors in a biochemical system. *BioSystems* 50 83–97. 10.1016/s0303-2647(98)00094-x 10367973

[B48] De la Fuente I. M. Martínez L. Aguirregabiria J. M. Veguillas J. Iriarte M. (1999a). Long-range correlations in the phase-shifts of numerical simulations of biochemical oscillations and in experimental cardiac rhythms. *J. Biol. Syst.* 7 113–130.

[B49] De la Fuente I. M. Martínez L. Pérez-Samartín A. L. Ormaetxea L. Amezaga C. Vera-López A. (2008). Global self-organization of the cellular metabolic structure. *PLoS One* 3:e3100. 10.1371/journal.pone.0003100 18769681 PMC2519785

[B50] De la Fuente I. M. Vadillo F. Pérez-Pinilla M. Vera-López A. Veguillas J. (2009). The number of catalytic elements is crucial for the emergence of metabolic cores. *PLoS One* 4:e7510. 10.1371/journal.pone.0007510 PMC2770363 19888419

[B51] De la Fuente I. M. (2010). Quantitative analysis of cellular metabolic dissipative, self-organized structures. *Int. J. Mol. Sci* 11 3540–3599. 10.3390/ijms11093540 20957111 PMC2956111

[B52] De la Fuente I. M. Vadillo F. Pérez-Samartín A. Pérez-Pinilla M. Bidaurrazaga J. Vera-López A. (2010). Global self-regulations of the cellular metabolic structure. *PLoS One* 5:e9484. 10.1371/journal.pone.0009484 20209156 PMC2830472

[B53] De la Fuente I. M. Cortés J. Pérez-Pinilla M. Ruiz-Rodríguez V. Veguillas J. (2011). The metabolic core and catalytic switches are fundamental elements in the self-regulation of the systemic metabolic structure of cells. *PLoS One* 6:e2722. 10.1371/journal.pone.0027224 22125607 PMC3220688

[B54] De la Fuente I. M. Cortés J. M. (2012). Quantitative analysis of the effective functional structure in yeast glycolysis. *PLoS One* 7:e30162. 10.1371/journal.pone.0030162 22393350 PMC3290614

[B55] De la Fuente I. M. Cortes J. M. Pelta D. A. Veguillas J. (2013). Attractor metabolic networks. *PLoS One* 8:e58284. 10.1371/journal.pone.0058284 23554883 PMC3598861

[B56] De la Fuente I. M. (2014). “Metabolic dissipative structures,” in *Systems Biology of Metabolic and Signaling Networks: Energy, Mass and Information Transfer*, eds Aon M. A. Saks V. Schlattner U. (New York, NY: Springer Books) 179–212.

[B57] De la Fuente I. M. Cortés J. M. Valero E. Desroches M. Rodrigues S. Malaina I. (2014). On the dynamics of the adenylate energy system: homeorhesis vs homeostasis. *PLoS One* 9:e108676. 10.1371/journal.pone.0108676 25303477 PMC4193753

[B58] De la Fuente I. M. (2015). Elements of the cellular metabolic structure. *Front. Mol. Biosc* 2:16. 10.3389/fmolb.2015.00016 25988183 PMC4428431

[B59] De la Fuente I. M. Bringas C. Malaina I. Fedetz M. Carrasco-Pujante J. Morales M. (2019a). Evidence of conditioned behavior in amoebae. *Nat. Commun.* 10:3690. 10.1038/s41467-019-11677-w 31417086 PMC6695432

[B60] De la Fuente I. M. Bringas C. Malaina I. Regner B. Pérez-Samartin A. Boyano M. D. (2019b). The nucleus does not significantly affect the migratory trajectories of amoeba in two-dimensional environments. *Sci. Rep.* 9:16369. 10.1038/s41598-019-52716-2 31704992 PMC6841717

[B61] De la Fuente I. M. López J. I. (2020). Cell motility and cancer. *Cancers* 12 2177. 10.3390/cancers12082177 32764365 PMC7464129

[B62] Dekhuijzen A. Bagust J. (1996). Analysis of neural bursting: nonrhythmic and rhythmic activity in isolated spinal cord. *J. Neurosci. Methods* 67 141–147. 10.1016/S0165-0270(96)00033-7 8872879

[B63] Deneke V. E. Melbinger A. Vergassola M. Di Talia S. (2016). Waves of Cdk1 activity in S phase synchronize the cell cycle in *Drosophila* embryos. *Dev. Cell* 38 399–412. 10.1016/j.devcel.2016.07.023 27554859 PMC4999262

[B64] Dodd M. S. Papineau D. Grenne T. Slack J. F. Rittner M. Pirajno F. (2017). Evidence for early life in Earth’s oldest hydrothermal vent precipitates. *Nature* 543 60–64. 10.1038/nature21377 28252057

[B65] Dou T.-Y. Luan H.-W. Ge G.-B. Dong M.-M. Zou H.-F. He Y.-Q. (2015). Functional and structural properties of a novel cellulosome-like multienzyme complex: efficient glycoside hydrolysis of water-insoluble 7-xylosyl-10-deacetylpaclitaxel. *Sci. Rep.* 5:13768. 10.1038/srep13768 26347949 PMC4562250

[B66] Dunaway-Mariano D. (2008). Enzyme function discovery. *Structure* 16 1599–1600. 10.1016/j.str.2008.10.001 19000810

[B67] Dussutour A. Latty T. Beekman M. Simpson S. J. (2010). Amoeboid organism solves complex nutritional challenges. *Proc. Natl. Acad. Sci. U.S.A.* 107 4607–4611. 10.1073/pnas.0912198107 20142479 PMC2842061

[B68] Ebeling W. Ulbricht H. (eds) (1986). *Self-Organization by Non-Linear Irreversible Processes.* Berlin: Springer-Verlag.

[B69] Einstein A. (1905). Zum gegenwärtigen Stand des Strahlungsproblesm. *Phys. Zeits.* 10 185–193.

[B70] Fenimore P. W. Frauenfelder H. McMahon B. H. Young R. D. (2004). Bulk-solvent and hydration-shell fluctuations, similar to alpha- and beta-fluctuations in glasses, control protein motions and functions. *P. Natl. Acad. Sci. U.S.A.* 101 14408–14413. 10.1073/pnas.0405573101 15448207 PMC521939

[B71] Fuentes J. M. Pascual M. R. Salido G. Soler J. A. (1994). Oscillations in rat liver cytosolic enzyme activities of the urea cycle. *Arch. Int. Physiol. Biochim Biophys.* 102 237–241. 10.3109/13813459409003936 7849268

[B72] Garmendia-Torres C. Goldbeter A. Jacquet M. (2007). Nucleocytoplasmic oscillations of the yeast transcription factor Msn2: evidence for periodic PKA activation. *Curr. Biol.* 17 1044–1049. 10.1016/j.cub.2007.05.032 17570669

[B73] Gavin A. C. Bosche M. Krause R. Grandi P. Marzioch M. Bauer A. (2002). Functional organization of the yeast proteome by systematic analysis of protein complexes. *Nature* 415 141–147. 10.1038/415141a 11805826

[B74] Gavin A. C. Superti-Furga G. (2003). Protein complexes and proteome organization from yeast to man. *Curr. Opin. Chem. Biol.* 7 21–27. 10.1016/S1367-5931(02)00007-8 12547422

[B75] Gennarino V. A. D’Angelo G. Dharmalingam G. Fernandez S. Russolillo G. Sanges R. (2012). Identification of microRNA-regulated gene networks by expression analysis of target genes. *Genome Res.* 22 1163–1172. 10.1101/gr.130435.111 22345618 PMC3371699

[B76] Geryk J. Slanina F. (2013). Modules in the metabolic network of *E. coli* with regulatory interactions. *Int. J. Data Min. Bioinform.* 8 188–202. 10.1504/IJDMB.2013.055500 24010267

[B77] Getty L. Panteleon A. E. Mittelman S. D. Dea M. K. Bergman R. N. (2000). Rapid oscillations in omental lipolysis are independent of changing insulin levels in vivo. *J. Clin. Invest.* 106 421–430. 10.1172/JCI7815 10930445 PMC314322

[B78] Getty-Kaushik L. Richard A. T. Corkey B. E. (2005). Free fatty acid regulation of glucose-dependent intrinsic oscillatory lipolysis in perifused isolated rat adipocytes. *Diabetes* 54 629–637. 10.2337/diabetes.54.3.629 15734837

[B79] Goldbeter A. (2002). Computational approaches to cellular rhythms. *Nature* 420 238–245. 10.1038/nature01259 12432409

[B80] Goldbeter A. (2007). Biological rhythms as temporal dissipative structures. *Adv. Chem. Phys.* 135 253–295.

[B81] Goldbeter A. (2018). Dissipative structures in biological systems: bistability, oscillations, spatial patterns and waves. *Philos. Trans. R. Soc. Lond. Ser. A Mat. Phys. Eng. Sci.* 376 20170376. 10.1098/rsta.2017.0376 29891498 PMC6000149

[B82] Gontero B. Lebreton S. Graciet E. (2018). “Multienzyme complexes involved in the benson–calvin cycle and in fatty acid metabolism,” in *Annual Plant Reviews Book Series*, eds Andrew C. A. William A. L. Michael T. M. (Weinheim: Wiley-VCH), 125–157.

[B83] Gonze D. Halloy J. Goldbeter A. (2004). Stochastic models for circadian oscillations: Emergence of a biological rhythm. *In.t J. Quantum Chem.* 98 228–238.

[B84] Graham D. M. Andersen T. Sharek L. Uzer G. Rothenberg K. Hoffman B. D. (2018). Enucleated cells reveal differential roles of the nucleus in cell migration, polarity, and mechanotransduction. *J. Cell Biol.* 217 895–914. 10.1083/jcb.201706097 29351995 PMC5839789

[B85] Greco T. M. Cristea I. M. (2016). The biochemical evolution of protein complexes. *Trends Biochem. Sci.* 41 4–6. 10.1016/j.tibs.2015.11.007 26682499 PMC4706493

[B86] Guthrie P. B. Knappenberger J. Segal M. Bennett M. V. L. Charles A. C. Kater S. B. (1999). ATP released from astrocytes mediates glial calcium waves. *J. Neurosci.* 19 520–528. 10.1523/JNEUROSCI.19-02-00520.1999 9880572 PMC6782195

[B87] Guttman R. Lewis S. Rinzel J. (1980). Control of repetitive firing in squid axon membrane as a model for a neuroneoscillator. *J. Physiol.* 305 377–395. 10.1113/jphysiol.1980.sp013370 7441560 PMC1282979

[B88] Halley J. D. Winkler D. A. (2008). Consistent concepts of self-organization and self-assembly. *Complexity* 14 10–17. 10.1002/cplx.20235

[B89] Hans M. A. Heinzle E. Wittmann C. (2003). Free intracellular amino acid pools during autonomous oscillations in Saccharomyces cerevisiae. *Biotechnol. Bioeng.* 82 143–151. 10.1002/bit.10553 12584756

[B90] Hao D. Ren C. Li C. (2012). Revisiting the variation of clustering coefficient of biological networks suggests new modular structure. *BMC Syst. Biol.* 6:34. 10.1186/1752-0509-6-34 22548803 PMC3465239

[B91] Harrington H. A. Ho K. L. Ghosh S. Tung K. C. (2008). Construction and analysis of a modular model of caspase activation in apoptosis. *Theor. Biol. Med. Model.* 5 26. 10.1186/1742-4682-5-26 19077196 PMC2672941

[B92] Hartig K. Beck E. (2005). Endogenous cytokinin oscillations control cell cycle progression of tobacco BY-2 cells. *Plant. Biol.* 7 33–40. 10.1055/s-2004-830474 15666212

[B93] Heltberg M. L. Krishna S. Jensen M. H. (2019). On chaotic dynamics in transcription factors and the associated effects in differential gene regulation. *Nat. Commun.* 10 71. 10.1038/s41467-018-07932-1 30622249 PMC6325146

[B94] Hertz J. A. Krogh A. S. Palmer R. G. (1991). *Introduction to the Theory of Neural Computation. Santa Fe Institute Series*. Boca Raton: CRC Press 352. 10.1201/9780429499661

[B95] Hilario E. Caulkins B. G. Huang Y.-M. M. You W. Chang C.-E. A. Mueller L. J. (2016). Visualizing the tunnel in tryptophan synthase with crystallography: insights into a selective filter for accommodating indole and rejecting water. *Biochim. Biophys. Acta* 1864 268–279. 10.1016/j.bbapap.2015.12.006 26708480 PMC4732270

[B96] Ho Y. Gruhler A. Heilbut A. Bader G. D. Moore L. Adams S. L. (2002). Systematic identification of protein complexes in Saccharomyces cerevisiae by mass spectrometry. *Nature* 415 180–183. 10.1038/415180a 11805837

[B97] Holz G. G. Heart E. Leech C. A. (2008). Synchronizing Ca2+ and cAMP oscillations in pancreatic beta cells: a role for glucose metabolism and GLP-1 receptors? Focus on “Regulation of cAMP dynamics by Ca2+ and G protein-coupled receptors in the pancreatic β-cell: a computational approach”. *Am. J. Physiol. Cell Physiol.* 294 C4–C6. 10.1152/ajpcell.00522.2007 17989206 PMC3501003

[B98] Hopfield J. J. (1982). Neural networks and physical systems with emergent collective computational abilities. *Proc. Nat. Acad. Sci. U.S.A.* 79 2554–2558. 10.1073/pnas.79.8.2554 6953413 PMC346238

[B99] Hoskins A. A. Anand R. Ealick E. E. Stubbe J. (2004). the formylglycinamide ribonucleotide amidotransferase complex from *Bacillus subtilis*: metabolite-mediated complex formation. *Biochemistry* 43 10314–10327. 10.1021/bi049127h 15301530

[B100] Hsu J. T. Peng C. H. Hsieh W. P. Lan C. Y. Tang C. Y. (2011). A novel method to identify cooperative functional modules: study of module coordination in the Saccharomyces cerevisiae cell cycle. *BMC Bioinform.* 12:281. 10.1186/1471-2105-12-281 21749690 PMC3143111

[B101] Hungerbuehler A. K. Philippsen P. Gladfelter A. S. (2007). Limited functional redundancy and oscillation of cyclins in multinucleated *Ashbya gossypii* fungal cells. *Eukaryot. Cell* 6 473–486. 10.1128/EC.00273-06 17122387 PMC1828934

[B102] Hyman A. A. Weber C. A. Jülicher F. (2014). Liquid-liquid phase separation in biology. *Ann. Rev. Cell Dev. Biol.* 30 39–58. 10.1146/annurev-cellbio-100913-013325 25288112

[B103] Ishii K. Hirose K. Iino M. (2006). Ca2+ shuttling between endoplasmic reticulum and mitochondria underlying Ca2+ oscillations. *EMBO Rep.* 7 390–396. 10.1038/sj.embor.7400620 16415789 PMC1456907

[B104] Izard T. Aevarsson A. Allen M. D. Westphal A. H. Perham R. N. de Kok A. (1999). Principles of quasi-equivalence and Euclidean geometry govern the assembly of cubic and dodecahedral cores of pyruvate dehydrogenase complexes. *Proc. Natl. Acad. Sci. U.S.A.* 96 1240–1245. 10.1073/pnas.96.4.1240 9990008 PMC15447

[B105] Jaffe L. F. (2002). On the conservation of fast calcium wave speeds. *Cell Calcium* 32 217–229. 10.1016/s0143416002001574 12379182

[B106] Jaffe L. F. (2008). Calcium waves. *Philos. Trans. R. Soc. Lond. B Biol. Sci.* 363 1311–1317. 10.1098/rstb.2007.2249 18192175 PMC2610120

[B107] Jules M. Francois J. Parrou J. L. (2005). Autonomous oscillations in Saccharomyces cerevisiae during batch cultures on trehalose. *FEBS J.* 272 1490–1500. 10.1111/j.1742-4658.2005.04588.x 15752364

[B108] Jung T. Grune T. (2012). Structure of the proteasome. *Prog. Mol. Biol. Transl. Sci.* 109 1–39. 10.1016/b978-0-12-397863-9.00001-8 22727418

[B109] Kaltenbach H. M. Stelling J. (2012). Modular analysis of biological networks. *Adv. Exp. Med. Biol.* 736 3–17. 10.1007/978-1-4419-7210-1_1 22161320

[B110] Kass L. Bray W. O. (1996). Kinetic model for phototransduction and G-protein enzyme cascade: understanding quantal bumps during inhibition of CaM-KII or PP2B. *J. Photochem. Photobiol. B Biol.* 35 105–113. 10.1016/1011-1344(96)07301-0 8823940

[B111] Kastritis P. L. Gavin A. C. (2018). Enzymatic complexes across scales. *Essays Biochem.* 62 501–514. 10.1042/EBC20180008 30315098 PMC6204551

[B112] Kauffman S. Johnsen S. (1991). Coevolution to the edge of chaos: Coupled fitness landscapes, poised states, and coevolutionary avalanches. *J. Theo. Biol.* 149 467–505. 10.1016/S0022-5193(05)80094-3 2062105

[B113] Keller M. Piedrafita G. Ralser M. (2015). The widespread role of non-enzymatic reactions in cellular metabolism. *Curr. Opin. Biotechnol* 34 153–161. 10.1016/j.copbio.2014.12.020 25617827 PMC4728180

[B114] Kim G. Yang J. Jang J. Choi J. Roe A. J. Olwyn B. (2020). Aldehyde-alcohol dehydrogenase undergoes structural transition to form extended spirosomes for substrate channeling. *Commun. Biol.* 3:298. 10.1038/s42003-020-1030-1 32523125 PMC7286902

[B115] Kindzelskii A. L. Zhou M. J. Haugland P. R. Boxer A. L. Petty R. H. (1998). Oscillatory pericellular proteolysis and oxidant deposition during neutrophil locomotion. *Biophys. J.* 74 90–97. 10.1016/S0006-3495(98)77770-7 9449313 PMC1299365

[B116] Kindzelskii A. L. Petty H. R. (2002). Apparent role of traveling metabolic waves in oxidant release by living neutrophils. *Proc. Natl. Acad. Sci. U.S.A.* 99 9207–9212. 10.1073/pnas.132630999 12082178 PMC123119

[B117] Klevecz R. R. Murray D. B. (2001). Genome wide oscillations in expression. Wavelet analysis of time series data from yeast expression arrays uncovers the dynamic architecture of phenotype. *Mol. Biol. Rep.* 28 73–82. 10.1023/A:1017909012215 11931391

[B118] Klevecz R. R. Bolen J. Forrest G. Murray D. B. (2004). A genomewide oscillation in transcription gates DNA replication and cell cycle. *Proc. Natl. Acad. Sci. U.S.A.* 101 1200–1205. 10.1073/pnas.0306490101 14734811 PMC337030

[B119] Klimontovich Y. L. (1999). Entropy and information of open systems. *Phys. Uspekhi.* 42 375–384. 10.1515/JNETDY.2008.006

[B120] Kohnhorst C. L. Kyoung M. Jeon M. Schmitt D. L. Kennedy E. L. Ramirez J. (2017). Identification of a multienzyme complex for glucose metabolism in living cells. *J. Biol. Chem.* 292 9191–9203. 10.1074/jbc.M117.783050 28424264 PMC5454101

[B121] Kondepudi D. Prigogine I. (2014). *Modern Thermodynamics: From Heat Engines to Dissipative Structures*, 2nd Edn. Weinheim: Wiley-VCH.

[B122] Korzeniewski B. Zoladz J. A. (2001). A model of oxidative phosphorylation in mammalian skeletal muscle. *Biophys. Chem.* 92 17–34. 11527576 10.1016/s0301-4622(01)00184-3

[B123] Korcsmáros T. Kovács I. A. Szalay M. S. Csermely P. (2007). Molecular chaperones: the modular evolution of cellular networks. *J. Biosci.* 32 441–446. 10.1007/s12038-007-0043-y 17536163

[B124] Kruse K. Jülicher F. (2005). Oscillations in cell biology. *Curr. Opin. Cell Biol.* 17 20–26. 10.1016/j.ceb.2004.12.007 15661515

[B125] Kühner S. van Noort V. Betts M. J. Leo-Macias A. Batisse C. Rode M. (2009). Proteome organization in a genome-reduced bacterium. *Science* 326 1235–1240. 10.1126/science.1176343 19965468

[B126] Küken A. Sommer F. Yaneva-Roder L. Mackinder L. C. Höhne M. Geimer S. (2018). Effects of microcompartmentation on flux distribution and metabolic pools in Chlamydomonas reinhardtii chloroplasts. *eLife* 7:e37960. 10.7554/eLife.37960 30306890 PMC6235561

[B127] Kurz F. T. Aon M. A. O’Rourke B. Armoundas A. A. (2010). Spatio-temporal oscillations of individual mitochondria in cardiac myocytes reveal modulation of synchronized mitochondrial clusters. *Proc. Natl. Acad. Sci. U.S.A.* 107 14315–14320. 10.1073/pnas.1007562107 20656937 PMC2922526

[B128] Kuzin A. M. (1970). *Structural – Metabolic Hypothesis in Radiobiology.* Moscow: Nauka.

[B129] Lahtvee P. Seiman A. Arike L. Adamberg K. Vilu R. (2014). Protein turnover forms one of the highest maintenance costs in *Lactococcus lactis*. *Microbiology* 160(Pt .7) 1501–1512. 10.1099/mic.0.078089-0 24739216

[B130] Lange G. Mandelkow E. M. Jagla A. Mandelkow E. (1988). Tubulin oligomers and microtubule oscillations. antagonistic role of microtubule stabilizers and destabilizers. *Eur. J. Biochem.* 178 61–69. 10.1111/j.1432-1033.1988.tb14429.x 3203694

[B131] Latty T. Beekman M. (2011). Speed-accuracy trade-offs during foraging decisions in the acellular slime mould Physarum polycephalum. *Proc. Biol. Sci.* 278 539–545. 10.1098/rspb.2010.1624 20826487 PMC3025689

[B132] Lloyd D. Eshantha L. Salgado J. Turner M. P. Murray D. B. (2002). Respiratory oscillations in yeast: clock-driven mitochondrial cycles of energization. *FEBS Lett.* 519 41–44. 10.1016/S0014-5793(02)02704-7 12023015

[B133] Lloyd D. Murray D. B. (2005). Ultradian metronome: timekeeper for orchestration of cellular coherence. *Trends Biochem. Sci.* 30 373–377. 10.1016/j.tibs.2005.05.005 15935677

[B134] Lloyd D. Murray D. B. (2006). The temporal architecture of eukaryotic growth. *FEBS Lett.* 580 2830–2835. 10.1016/j.febslet.2006.02.066 16545376

[B135] Mackintosh N. J. (1983). *Conditioning and Associative Learning.* Oxford: Clarendon Press.

[B136] Maier T. Jenni S. Ban N. (2006). Architecture of mammalian fatty acid synthase at 4.5 A resolution. *Science* 311 1258–1262. 10.1126/science.1123248 16513975

[B137] Markevich N. I. Tsyganov M. A. Hoek J. B. Kholodenko B. N. (2006). Long−range signaling by phosphoprotein waves arising from bistability in protein kinase cascades. *Mol. Syst. Biol.* 2 61. 10.1038/msb4100108 17102806 PMC1682027

[B138] Márquez S. Crespo P. Carlini V. Garbarino-Pico E. Baler R. Caputto B. L. (2004). The metabolism of phospholipids oscillates rhythmically in cultures of fibroblasts and is regulated by the clock protein PERIOD 1. *FASEB J.* 18 519–521. 10.1096/fj.03-0417fje 14715703

[B139] Misteli T. (2009). Self-organization in the genome. *Proc. Natl. Acad. Sci. U.S.A.* 106 6885–6886. 10.1073/pnas.0902010106 19416923 PMC2678480

[B140] Miyaji T. Ohnishi I. (2008). Physarum can solve the shortest path problem on riemannian surface mathematically rigourously. *Int. J. Pure App. Math.* 47 353–369.

[B141] Møller A. C. Hauser M. J. B. Olsen L. F. (1998). Oscillations in peroxidase-catalyzed reactions and their potential function in vivo. *Biophys. Chem.* 72 63–72. 10.1016/S0301-4622(98)00123-9 17029705

[B142] Mogilevskaya E. Demin O. Goryanin I. (2006). Kinetic model of mitochondrial krebs cycle: unraveling the mechanism of salicylate hepatotoxic effects. *J. Biol. Phys.* 32 245–271. 19669466 10.1007/s10867-006-9015-y PMC2651525

[B143] Monge C. Grichine A. Rostovtseva T. Sackett P. Saks V. A. (2009). Compartmentation of ATP in cardiomyocytes and mitochondria. kinetic studies and direct measurements. *Biophys. J.* 96:241a. 10.1016/j.bpj.2008.12.1188

[B144] Murray D. Beckmann M. Kitano H. (2007). Regulation of yeast oscillatory dynamics. *Proc. Natl. Acad. Sci. U.S.A.* 104 2241–2246. 10.1073/pnas.0606677104 17284613 PMC1794218

[B145] Muto A. Kotera M. Tokimatsu T. Nakagawa Z. Goto S. Kanehisa M. (2013). Modular architecture of metabolic pathways revealed by conserved sequences of reactions. *J. Chem. Inf. Model.* 53 613–622. 10.1021/ci3005379 23384306 PMC3632090

[B146] Nakagaki T. Yamada H. Toth A. (2000). Maze-solving by an amoeboid organism. *Nature* 407:470. 10.1038/35035159 11028990

[B147] Nakagaki T. (2001). Smart behavior of true slime mold in a labyrinth. *Res. Microbiol.* 152 767–770. 10.1016/S0923-2508(01)01259-1 11763236

[B148] Nakagaki T. Kobayashi R. Ueda T. Nishiura Y. (2004a). Obtaining multiple separate food sources: behavioural intelligence in the *Physarum plasmodium*. *Proc. Biol. Sci.* 271 2305–2310. 10.1098/rspb.2004.2856 15539357 PMC1691859

[B149] Nakagaki T. Yamada H. Hara M. (2004b). Smart network solutions in an amoeboid organism. *Biophys. Chem.* 107 1–5. 10.1016/S0301-4622(03)00189-3 14871595

[B150] Nakamura N. Wei J. H. Seemann J. (2012). Modular organization of the mammalian *Golgi apparatus*. *Curr. Opin. Cell. Biol.* 24 467–474. 10.1016/j.ceb.2012.05.009 22726585 PMC3495003

[B151] Nakamura Y. (2018). Membrane Lipid oscillation: an emerging system of molecular dynamics in the plant membrane. *Plant Cell Physiol.* 59 441–447. 10.1093/pcp/pcy023 29415166

[B152] Negrete J. Pumir A. Hsu H.-F. Westendorf C. Tarantola M. Beta C. (2016). Noisy oscillations in the actin cytoskeleton of chemotactic amoeba. *Phys. Rev. Lett.* 117 148102. 10.1103/PhysRevLett.117.148102 27740793

[B153] Neph S. Stergachis A. B. Reynolds A. Sandstrom R. Borenstein E. Stamatoyannopoulos J. A. (2012). Circuitry and dynamics of human transcription factor regulatory networks. *Cell* 150 1274–1286. 10.1016/j.cell.2012.04.040 22959076 PMC3679407

[B154] Newman E. A. (2001). Propagation of intercellular calcium waves in retinal astrocytes and müller cells. *J. Neurosci.* 21 2215–2223. 10.1523/JNEUROSCI.21-07-02215.2001 11264297 PMC2409971

[B155] Niebel B. Leupold S. Heinemann M. (2019). An upper limit on gibbs energy dissipation governs cellular metabolism. *Nat. Metab.* 1 125–132. 10.1038/s42255-018-0006-7 32694810

[B156] Nicolis G. Prigogine I. (1977). *Self-Organization in Nonequilibrium Systems. From Dissipative Structures to Order Through Fluctuations.* New York, NY: Wiley.

[B157] Noble D. Jablonka E. Joyner M. J. Müller G. B. Omholt S. W. (2014). Evolution evolves: physiology returns to centre stage. *J. Physiol.* 592(Pt. 11) 2237–2244. 10.1113/jphysiol.2014.273151 24882808 PMC4048083

[B158] Nolet F. E. Vandervelde A. Vanderbeke A. Piñeros L. Chang J. B. Gelens L. (2020). Nuclei determine the spatial origin of mitotic waves. *eLife* 9:e52868. 10.7554/eLife.52868 32452767 PMC7314552

[B159] Nurse P. (2008). Life, logic and information. *Nature* 454 424–426. 10.1038/454424a 18650911

[B160] Mathieson T. Franken H. Kosinski J. Kurzawa N. Zinn N. Sweetman G. (2018). Systematic analysis of protein turnover in primary cells. *Nat. Commun.* 9:689. 10.1038/s41467-018-03106-1 29449567 PMC5814408

[B161] Olsen L. F. Degn H. (1985). Chaos in biological systems. *Q. Rev. Biophys.* 18 165–225. 10.1017/s0033583500005175 3912797

[B162] Ovádi J. Srere P. A. (2000). Macromolecular compartmentation and channeling. *Int. Rev. Cytol.* 192 255–280. 10.1016/S0074-7696(08)60529-X 10553282

[B163] Ovádi J. Saks V. (2004). On the origin of the ideas of intracellular compartmentation and organized metabolic systems. *Mol. Cell. Biochem.* 256 5–12. 10.1023/B:MCBI.0000009855.14648.2c 14977166

[B164] Ozalp V. C. Pedersen T. R. Nielsen L. J. Olsen L. F. (2010). Time-resolved measurements of intracellular ATP in the yeast Saccharomyces cerevisiae using a new type of nanobiosensor. *J. Biol. Chem.* 285 37579–37588. 10.1074/jbc.M110.155119 20880841 PMC2988363

[B165] Pang C. N. Krycer J. R. Lek A. Wilkins M. R. (2008). Are protein complexes made of cores, modules and attachments? *Proteomics* 8 425–434. 10.1002/pmic.200700801 18175372

[B166] Pavlov I. P. (1927). *Conditioned Reflexes: An Investigation of the Physiological Activity of the Cerebral Cortex.* Oxford: Oxford Univ. Press. 10.5214/ans.0972-7531.1017309 PMC4116985 25205891

[B167] Pêkalski J. Zuk P. J. Kochańczyk M. Junkin M. Kellogg R. Tay S. (2013). Spontaneous NF-κB activation by autocrine TNFα signaling: a computational analysis. *PLoS One* 8:e78887. 10.1371/journal.pone.0078887 24324544 PMC3855823

[B168] Peretto P. (1992). *An Introduction to the Modeling of Neural Networks*. Cambridge: Cambridge University Press.

[B169] Petrovic A. Mosalaganti S. Keller J. Mattiuzzo M. Overlack K. Krenn V. (2014). Modular assembly of RWD domains on the Mis12 complex underlies outer kinetochore organization. *Mol. Cell* 53 591–605. 10.1016/j.molcel.2014.01.019 24530301

[B170] Petty H. R. Worth R. G. Kindzelskii A. L. (2000). Imaging sustained dissipative patterns in the metabolism of individual living cells. *Phys. Rev. Lett.* 84 2754–2757. 10.1103/PhysRevLett.84.2754 11017317

[B171] Placantonakis D. G. Welsh J. P. (2001). Two distinct oscillatory states determined by the NMDA receptor in rat inferior olive. *J. Physiol.* 534(Pt. 1) 123–140. 10.1111/j.1469-7793.2001.t01-1-00123.x 11432997 PMC2278697

[B172] Postma M. Bosgraaf L. Loovers H. M. Van Haastert P. J. (2004). Chemotaxis: signalling modules join hands at front and tail. *EMBO Rep.* 5 35–40. 10.1038/sj.embor.7400051 14710184 PMC1298962

[B173] Radzicka A. Wolfenden R. (1995). A proficient enzyme. *Science* 267 90–93. 10.1126/science.7809611 7809611

[B174] Ramanathan A. Savol A. Burger V. Chennubhotla C. S. Agarwal P. K. (2014). Protein conformational populations and functionally relevant substates. *Acc. Chem. Res.* 47 149–156. 10.1021/ar400084s 23988159

[B175] Ravasz E. Somera A. L. Mongru D. A. Oltvai Z. N. Barabási A. L. (2002). Hierarchical organization of modularity in metabolic networks. *Science* 297 1551–1555. 10.1126/science.1073374 12202830

[B176] Rengan R. Omann G. M. (1999). Regulation of oscillations in filamentous actin content in polymorphonuclear leukocytes stimulated with leukotriene B4 and platelet-activating factor. *Biochem. Biophys. Res. Commun.* 262 479–486. 10.1006/bbrc.1999.1222 10462500

[B177] Rieß B. Grötsch R. K. Boekhoven J. (2020). The design of dissipative molecular assemblies driven by chemical reaction cycles. *Chemistry* 6 552–578. 10.1016/j.chempr.2019.11.008

[B178] Ringe D. Petsko G. A. (2009). How enzymes work. *Science* 320 1428–1429. 10.1126/science.1159747 18556536

[B179] Romashko D. N. Marban E. O’Rourke B. (1998). Subcellular metabolic transients and mitochondrial redox waves in heart cells. *Proc. Natl. Acad. Sci. U.S.A.* 95 1618–1623. 10.1073/pnas.95.4.1618 9465065 PMC19119

[B180] Ross A. B. Langer J. D. Jovanovic M. (2020). Proteome turnover in the spotlight: approaches, applications, and perspectives. *Mol. Cell Proteomics.* 20:00016. 10.1074/mcp.R120.002190 33556866 PMC7950106

[B181] Roussel M. R. Ivlev A. A. Igamberdiev A. U. (2006). Oscillations of the internal CO2 concentration in tobacco leaves transferred to low CO2. *J. Plant Physiol.* 34 1188–1196. 10.1016/j.jplph.2006.08.004 17007962

[B182] SabziNezhad A. Jalili S. (2020). DPCT: a dynamic method for detecting protein complexes From TAP-aware weighted PPI network. *Front. Genet* 11:567. 10.3389/fgene.2020.00567 32676097 PMC7333736

[B183] Saigusa T. Tero A. Nakagaki T. Kuramoto Y. (2008). Amoebae anticipate periodic events. *Phys. Rev. Lett.* 100:018101. 10.1103/PhysRevLett.100.01810 18232821

[B184] Saks V. A. Monge C. Anmann T. Dzeja P. (2007). “integrated and organized cellular energetic systems: theories of cell energetics, compartmentation and metabolic channeling,” in *Molecular System Bioenergetics. Energy for Life*, ed. Saks V. A. (Weinheim: Wiley-VCH), 59–110.

[B185] Saks V. A. Monge C. Guzun R. (2009). Philosophical basis and some historical aspects of systems biology: From Hegel to Noble – Applications for bioenergetic research. *Int. J. Mol. Sci.* 10 1161–1192. 10.3390/ijms10031161 19399243 PMC2672024

[B186] San Román M. Cancela H. Acerenza L. (2014). Source and regulation of flux variability in *Escherichia coli*. *BMC Syst. Biol.* 8:67. 10.1186/1752-0509-8-67 24927772 PMC4074586

[B187] Sánchez-Armáss S. Sennoune S. R. Maiti D. Ortega F. Martínez-Zaguila R. (2006). Spectral imaging microscopy demonstrates cytoplasmic pH oscillations in glial cells. *Am. J. Physiol. Cell Physiol.* 290 C524–C538. 10.1152/ajpcell.00290.2005 16135543

[B188] Santillán M. Mackey M. C. (2008). Quantitative approaches to the study of bistability in the lac operon of *Escherichia coli*. *J. R. Soc. Interf.* 5(Suppl 1) S29–S39. 10.1098/rsif.2008.0086.focus 18426771 PMC2504340

[B189] Sartori P. Leibler S. (2020). Lessons from equilibrium statistical physics regarding the assembly of protein complexes. *P. Natl. Acad. Sci.* 117 114–120. 10.1073/pnas.1911028117 31871201 PMC6955335

[B190] Scemes E. Giaume C. (2006). Astrocyte calcium waves: what they are and what they do. *Glia* 54 716–725. 10.1002/glia.20374 17006900 PMC2605018

[B191] Schreiber T. (2000). Measuring information transfer. *Phys. Rev. Lett.* 85 461–464. 10.1103/PhysRevLett.85.461 10991308

[B192] Schubert V. (2014). RNA polymerase II forms transcription networks in rye and *Arabidopsis* nuclei and its amount increases with endopolyploidy. *Cytogenet. Genome Res.* 143 69–77. 10.1159/000365233 25060696

[B193] Serre V. Guy H. Liu X. Penverne B. Hervé G. Evans D. (1998). Allosteric regulation and substrate channeling in multifunctional pyrimidine biosynthetic complexes: analysis of isolated domains and yeast-mammalian chimeric proteins. *J. Mol. Biol.* 281 363–377. 10.1006/jmbi.1998.1856 9698553

[B194] Shankaran H. Ippolito D. L. Chrisler W. B. Resat H. Bollinger N. Opresko L. K. (2009). Rapid and sustained nuclear–cytoplasmic ERK oscillations induced by epidermal growth factor. *Mol. Syst. Biol.* 5:332. 10.1038/msb.2009.90 19953086 PMC2824491

[B195] Shimizu T. S. Tu Y. Berg H. C. (2010). A modular gradient-sensing network for chemotaxis in *Escherichia coli* revealed by responses to time-varying stimuli. *Mol. Syst. Biol.* 6 382. 10.1038/msb.2010.37 20571531 PMC2913400

[B196] Slaby O. Lebiedz D. (2009). Oscillatory NAD(P)H waves and calcium oscillations in neutrophils? A modeling study of feasibility. *Biophys. J.* 96 417–428. 10.1016/j.bpj.2008.09.044 19167293 PMC2716467

[B197] Smrcinová M. Sørensen P. G. Krempasky J. Ballo P. (1998). Chaotic oscillations in a chloroplast system under constant illumination. *Int. J. Bifurcat. Chaos.* 8 2467–2470. 10.1142/S0218127498001984

[B198] Srere P. A. (1972). “Is there an organization of krebs cycle enzymes in the mitochondrial matrix?,” in *Energy Metabolism and the Regulation of Metabolic Processes in Mitochondria*, eds Hanson R. W. Mehlman W. A. (New York, NY: Academic Press), 79–91.

[B199] Srere P. A. (1985). The metabolon. *Trends Biochem. Sci.* 10 109–110. 10.1016/0968-0004(85)90266-X

[B200] Srere P. A. (1987). Complexes of sequential metabolic enzymes. *Annu. Rev. Biochem.* 56 89–124. 10.1146/annurev.bi.56.070187.000513 2441660

[B201] Stryer L. Berg J. M. Tymoczko J. L. (2002). *Biochemistry*, 5th Edn. San Francisco, CA: W.H. Freeman.

[B202] Sun M. Leyh T. S. (2006). Channeling in sulfate activating complexes. *Biochemistry* 45 11304–11311. 10.1021/bi060421e 16981690

[B203] Svedružić Z. M. Odorèić I. Chang C. H. Svedružić D. (2020). Substrate channeling via a transient protein-protein complex: the case of D-Glyceraldehyde-3-phosphate dehydrogenase and L-lactate dehydrogenase. *Sci. Rep.* 10:10404. 10.1038/s41598-020-67079-2 32591631 PMC7320145

[B204] Sweetlove L. J. Fernie A. R. (2018). The role of dynamic enzyme assemblies and substrate channelling in metabolic regulation. *Nat. commun.* 9:2136. 10.1038/s41467-018-04543-8 29849027 PMC5976638

[B205] Tero A. Takagi S. Saigusa T. Ito K. Bebber D. P. Fricker M. D. (2010). Rules for biologically inspired adaptive network design. *Science* 327 439–442. 10.1126/science.1177894 20093467

[B206] Thoden J. B. Holden H. M. Wesenberg G. Raushel F. M. Rayment I. (1997). Structure of carbamoyl phosphate synthetase: a journey of 96 Å from substrate to product. *Biochemistry* 36 6305–6316. 10.1021/bi970503q 9174345

[B207] Tian B. Nowak D. E. Brasier A. R. (2005). A TNF-induced gene expression program under oscillatory NF-κB control. *BMC Genomics* 6:137. 10.1186/1471-2164-6-137 16191192 PMC1262712

[B208] Tonozuka H. Wang J. Mitsui K. Saito T. Hamada Y. Tsurugi K. (2001). Analysis of the upstream regulatory region of the GTS1 gene required for its oscillatory expression. *J. Biochem.* 130 589–595. 10.1093/oxfordjournals.jbchem.a003023 11686920

[B209] Tretter L. Adam-Vizi V. (2005). Alpha-ketoglutarate dehydrogenase: a target and generator of oxidative stress. *Philos. Trans. R. Soc. Lond. B. Biol. Sci.* 360 2335–2345. 10.1098/rstb.2005.1764 16321804 PMC1569585

[B210] Tsai S. Ames B. D. (2009). Structural enzymology of polyketide synthases. *Methods Enzymol.* 459 17–47. 10.1016/S0076-6879(09)04602-3 19362634 PMC2873140

[B211] Tu Y. Shimizu T. S. Berg H. C. (2008). Modeling the chemotactic response of *Escherichia coli* to time-varying stimuli. *Proc. Natl. Acad. Sci. U.S.A* 105 14855–14860. 10.1073/pnas.0807569105 18812513 PMC2551628

[B212] Tuttle L. M. Salis H. Tomshine J. Kaznessis Y. N. (2005). Model-driven designs of an oscillating gene network. *Biophys. J.* 89 3873–3883. 10.1529/biophysj.105.064204 16183880 PMC1366954

[B213] Tyson J. J. (1991). Modeling the cell division cycle: cdc2 and cyclin interactions. *PNAS* 88 7328–7332. 1831270 10.1073/pnas.88.16.7328 PMC52288

[B214] Tyson J. J. (2002). “Biochemical oscillations,” in *Computational Cell Biology. Interdisciplinary Applied Mathematics*, Vol. 20 eds Fall C. P. Marland E. S. Wagner J. M. Tyson J. J. (New York, NY: Springer), 230–260.

[B215] Ueda T. Nakagaki T. Yamada T. (1990). Dynamic organization of ATP and birefringent fibrils during free locomotion and galvanotaxis in the plasmodium of Physarum polycephalum. *J. Cell. Biol.* 110 1097–1102. 10.1083/jcb.110.4.1097 2324194 PMC2116098

[B216] Valero E. Maciá H. de la Fuente I. M. Hernandez J. A. González-Sánchez M. I García-Carmona F. (2016). Modeling the under light/dark conditions. *BMC Syst. Biol.* 10:11. 10.1186/s12918-015-0239-y 26797294 PMC4722729

[B217] von Stockar U. (2010). Biothermodynamics of live cells: a tool for biotechnology and biochemical engineering. *J. Non Equil. Therm.* 35 415–475. 10.1515/JNETDY.2010.024

[B218] Vicker M. G. (2002). Eukaryotic cell locomotion depends on the propagation of self-organized reaction-diffusion waves and oscillations of actin filament assembly. *Exp. Cel. Res.* 275 54–66. 10.1006/excr.2001.5466 11925105

[B219] Vlasova-St Louis I. Bohjanen P. R. (2011). Coordinate regulation of mRNA decay networks by GU-rich elements and CELF1. *Curr. Opin. Genet. Dev.* 21 444–451. 10.1016/j.gde.2011.03.002 21497082 PMC3146975

[B220] Watford M. (1989). Channeling in the urea cycle: a metabolon spanning two compartments. *Trends Biochem. Sci.* 14 313–314. 10.1016/0968-0004(89)90156-4 2678629

[B221] Wettmann L. Karsten K. (2018). The min-protein oscillations in *Escherichia coli*: An example of self-organized cellular protein waves. *Philos. Trans. R. Soc. Lond. B. Biol. Sci* 373 20170111. 10.1098/rstb.2017.0111 29632263 PMC5904297

[B222] Whitesides G. M. Grzybowski B. (2002). Self-assembly at all scales. *Science* 295 2418–2421. 10.1126/science.1070821 11923529

[B223] Wijnen H. Young W. M. (2006). Interplay of circadian clocks and metabolic rhythms. *Annu Rev Genet.* 40 409–448. 10.1146/annurev.genet.40.110405.090603 17094740

[B224] Wittmann C. Hans M. Van Winden A. W. Ras C. Heijnen J. J. (2005). Dynamics of intracellular metabolites of glycolysis and TCA cycle during cell-cycle-related oscillation in Saccharomyces cerevisiae. *Biotechnol. Bioeng.* 89 839–847. 10.1002/bit.20408 15690349

[B225] Xiong W. Ferrell J. E. (2003). A positive-feedback-based bistable “memory module” that governs a cell fate decision. *Nature* 426 460–465. 10.1038/nature02089 14647386

[B226] Yang C. R. Shapiro B. E. Hung S. P. Mjolsness E. D. Hatfield G. W. (2005). A mathematical model for the branched chain amino acid biosynthetic pathways of *Escherichia coli* K12. *J Biol Chem.* 280 11224–11232. 15657047 10.1074/jbc.M411471200

[B227] Yanling X. Qingming T. Guangjin Ch Mengru X. Shaoling Y. Jiajia Z. (2019). New insights into the circadian rhythm and its related diseases. *Front. Physiol* 10:682. 10.3389/fphys.2019.00682 31293431 PMC6603140

[B228] Yoon J. Si Y. Nolan R. Lee K. (2007). Modular decomposition of metabolic reaction networks based on flux analysis and pathway projection. *Bioinformatics* 23 2433–2440. 10.1093/bioinformatics/btm374 17660208

[B229] Zhabotinsky A. M. (2000). Bistability in the Ca(2+) calmodulin-dependent protein kinase-phosphatase system. *Biophys. J.* 79 2211–2221. 10.1016/S0006-3495(00)76469-1 11053103 PMC1301111

[B230] Zhang Y. Fernie A. R. (2020). Metabolons, enzyme–enzyme assemblies that mediate substrate channeling, and their roles in plant metabolism. *Plant Commun.* 2:100081. 10.1016/j.xplc.2020.100081 33511342 PMC7816073

[B231] Zhou L. Aon M. A. Almas T. Cortassa S. Winslow R. L. O’Rourke B. (2010). A reaction-diffusion model of ros-induced ros release in a mitochondrial network. *PLoS Comput. Biol.* 6:e1000657. 10.1371/journal.pcbi.1000657 20126535 PMC2813265

[B232] Zhu L. Aono M. Kim S.-J. Hara M. (2011). Problem-sizes scalability of amoeba-based neurocomputer for traveling salesman problem. *Proc. NOLTA* 45 108–111. 10.34385/proc.45.A2L-B4

