# Supplementary material for: Self-Organization and Information Processing: From Basic Enzymatic Activities to Complex Adaptive Cellular Behavior
Source: Front Genet. 2021 May 21;12:644615. doi: 10.3389/fgene.2021.644615 (PMC8176287; doi:10.3389/fgene.2021.644615)
Supplement: Supplementary file 1 [file Data_Sheet_1.docx]

**Supplementary Material 01**

**Systemic Molecular Turnover, the fundamental property of cellular metabolic life.**

All molecular components of the cell are synthesized and degraded continually following sophisticated interdependent processes that defy the human intellect. This continuous molecular turnover is produced by a super complex dynamic system formed by millions of biochemical reactions, which occur continuously at every moment of cellular life. The continuous recycling and incessant chemical transformations, that encompass practically all molecules, shape a critical scenario, only in which cellular life is possible (De la Fuente, 2015).

The consequences of self-ordered and self-regulated turnover dynamics are adequate cellular growth, the development of all physiological processes to maintain its functional structures, continuous adaptation to the environment, and mitosis.

**01. Proteome turnover**

All proteins are dynamic cellular constituents of the cell, they are being continually synthesized and destroyed through complex metabolic processes in which each protein is broken down partially, into peptides, or completely, into amino acids to later be regenerated "de novo" (Doherty et al., 2009; Qingbo, 2010; Mathieson et al., 2018; Christiano et al., 2020).

Proteins represent the largest molecular cellular component, corresponding to around 50-60 % of the cell’s dry weight (50-55% in *E. coli* and 60% in mammalian cell) (Delgado et al., 2013). As a consequence of global turnover processes, the entire proteome behaves as a dynamic system, in permanent recycling, exhibiting a high proteolytic activity in every moment of its cellular life. For instance, it has been found that of approximately 10^9^ proteins that integrate the proteome of a typical mouse L929 fibrosarcoma cell, 10^6^ are degraded every minute (Princiotta et al., 2003).

Further experimental evidence has shown that a remarkable number of proteins (more than 30%) are degraded by proteasomes shortly after their synthesis (Schubert et al., 2000). Even when a cell is not undergoing a period of growth, the protein pool is in a dynamic state, and the proteolytic machinery continues its relentless process of molecular destruction, requiring for it a significant amount of energy in form of ATP (Eagle et al., 1959; Doherty and Beynon, 2006). Studies in Lactococcus lactis have estimated that protein turnover alone costs between 38 and 47% of the total energy produced by this kind of cell (Lahtvee, 2014).

Inside the cell, each protein has a lifetime that falls within a broad range, from a few minutes to hours and days (Doherty et al., 2009; Schwanhausser et al., 2011; Geva-Zatorsky et al., 2012). Other studies have allowed us to know the synthesis-degradation rates in large numbers of proteins. For example, Belle et al., reported that the average turnover rates of over 3,750 proteins in the yeast proteome were of about 43 min, while an unexpected number of 161 proteins showed a half-life of 4 min (Belle et al., 2006). A similar distribution was observed by Yen et al., (2008), who reported that the turnover rates of over 8000 GFP-tagged human proteins also exhibited a bimodal pattern, with average turnover values of 30 min and 2 h respectively. Moreover, the measure of the half-life dynamics of 100 proteins in human cancer cells (H1299) under normal and stress condi­tions also showed two general patterns with half-lives ranging between 45 minutes and 22.5 hours (Eden et al, 2011).

In another extensive study of 80,098 peptides obtained from 8,041 HeLa proteins, approximately 60% of proteins presented half-life values clustered within 5 h of the average turnover rate of 20 h. In the nucleolus, a distinct group of proteins, mostly corresponding to ribosomal proteins, exhibited a fast turnover rate (<6 h) (Boisvert et al., 2012). In HeLa cells, proteins presenting slow turnover rates have a wide variety of functions such as chromosome organization, RNA processing, tRNA metabolic processes, translation, intracellular transport and mitochondrial membrane organization; these proteins are also commonly present in multiprotein complexes, such as ribosome and spliceosome subunits, proteasome, nuclear pore, exosome and RNA polymerases. On the other hand, proteins with faster turnover are also involved in a wide range of different metabolic processes including ribosome biogenesis, apoptosis, mitosis, cell cycle, cytoskeleton organization, DNA repair, chromosome segregation and nucleotide binding. A general characteristic of protein subunits belonging to multiprotein complexes is that they also show high-speed turnover as free proteins, before they go through the complex assembly sets (Boisvert et al., 2012).

Comparatively, a small number of proteins display degradation rates more than threefold faster than the proteome average. For instance, one rapidly degraded protein is ornithine decarboxylase, which has a half-life of 11 minutes (Russell and Snyder, 1969). Also, the half-life of unbound IκBα, negative feedback regulator of NFκB, is less than 15 minutes (O'Dea et al., 2007). And the p53 protein has a half-life of less than 30 minutes (Reihsaus et al., 1990).

Although many intracellular proteins have similar degradation rates, turnover values for individual proteins can vary depending on different conditions, such as, for example the cell cycle stage (Reed, 2003), post-translational modifications processes (García-Alai et al., 2006), subcellular localization (Larance et al., 2013), specific signals (Loriaux et al., 2013) and stress-response (Sommer and Wolf, 1997; Zhou and Gottesman, 1998; Eden et al, 2011), therefore, in general, none of the degradation rate values represent “absolute” values.

In eukaryotic cells, the major protein degradation pathways correspond to the ubiquitin–proteasome system and autophagy (Kraft et al., 2008). In general, the short-lived proteins are degraded by the ubiquitin/proteasome system (Hershko and Ciechanover, 1998) which involves proteins marked for destruction by the covalent attachment of ubiquitin chains. Polyubiquitinated proteins are then degraded by a large, [ATP](http://www.ncbi.nlm.nih.gov/books/n/stryer/A5607/def-item/A5617/)-dependent complex called the proteasome. Consequently, this process requires a strict regulation and recognition of target proteins with consumption of energy by means of ATP hydrolysis (Lecker and Mitch, 2011; Doherty and Beynon, 2006). Any part of the cell is not outside the reach of the ubiquitin-proteasome regulatory system. Concentration levels of proteins in the nucleus, cytosol, ER lumen, as well as membrane proteins, are all kept in place by ubiquitinating enzymes and proteasome (Glickman and Ciechanover, 2002). Another main protein degradation system is autophagy. A portion of cytoplasm including organelle structures is enclosed by a double membrane, subsequently fuses with a lysosome and ultimately digested by it (Mizushima et al., 2002). Autophagy represents the major intracellular pathway for the degradation and recycling of long-lived proteins and cytoplasmic organelles (Kelekar, 2005). About 30% of cytosolic proteins can be degraded by chaperone-mediated autophagy. Therefore, chaperones also play an important role in the regulation of protein degradation under different physiological conditions (Winchester, 2005).

There are other cytosolic proteolytic systems in mammalian cells, for example, acid proteases in the lysosomal compartment, caspases (cysteine proteases), which are critical in destruction of determined cell constituents during apoptosis and, Ca^2+^ dependent calpains, which seem to be activated when intracellular levels of Ca^2+^ rise in response to injury, therefore having a potentially important role in necrosis and autolysis (Lecker et al., 2006).

Proteins continuously synthesized and degraded throughout the cell cycle in such a way that rigorous control of proteolysis is required to regulate the immensely different and fluctuating half-lives. Cells can alter their protein levels via phosphorylation and other reversible post-translational modifications (PTMs) (García-Alai et al., 2006; Zhou et al., 2013).

Post-translational modification (PTM) is an essential, highly dynamic enzymatic process, which modifies protein functionality, reversibly transforming its structure by adding specific biochemical marks. There are different types of PTMs, e.g., phosphorylation, methylation, glycosylation, acetylation, ubiquitination, SUMOylation, etc., and the number and abundance of these modifications is constantly being updated (Khoury et al., 2011).

Protein phosphorylation is one of the most extensively studied PTM, and the phosphorylation-dephosphorylation mechanism is assumed to take place on a fast time scale in comparison to protein half-lives. Different sites on the same protein can be continuously phosphorylated-dephosphorylated. These events are not distributed along the whole protein structure but are instead constrained to sites of high accessibility and structural flexibility (Gnad et al., 2007), which allows changes in the chemical composition of proteins by adding or removing molecular marks (Thomson and Gunawardena, 2009). In different studies, more than 30,000 distinct sites of phosphorylation (Batth et al., 2014), and around 19,000 sites of ubiquitylation have been identified on about 5,000 proteins (Kim et al., 2011).

Protein turnover is also essential for the maintenance of dynamic cell metabolism in bacteria (Trötschel et al., 2012; Trötschel et al., 2013; Boubakri 2015). While eukaryotic cells use the ubiquitin-tagging machinery to recognize proteins and mark them for degradation by the proteasome, prokaryotes substrate selection is mainly operated at the level of the protease complex itself or together with a chaperone (Gur et al., 2011) or with an adaptor protein which directly interacting with both the substrate and the protease ATPase domains (Battesti and Gottesman, 2013).

Protease complexes are located in every main cellular compartment, that is, in the cytoplasm, cell membrane and periplasm (Gottesman et al., 1997; Lupas et al., 1997; Michalik et al., 2009). Gram negative bacteria have five main ATP-dependent proteases: ClpAP, ClpXP, Lon, HslUV and FtsH, while several Gram-positive bacteria exhibit additional proteases such as ClpCP, ClpEP as well as the bacterial proteasome (Gur et al., 2011). Archaea and some actinobacteria, like mycobacteria, possess a proteasome in addition to the aforementioned proteases (Maupin-Furlow, 2011; Humbard et al., 2013). As in eukaryotes, in prokaryotes, proteolysis is controlled by multiple factors that determine the rate of degradation (Trötschel et al., 2012). Besides, posttranslational modifications have also been widely described in bacteria (Cain et al., 2014; Li et al., 2014).

The turnover rate of proteins in bacteria has been quantified in a wide number of studies. For instance, in *E. coli*, a portion of the soluble protein fraction was analyzed showing a highly dynamic protein turnover with synthesis/degradation ratios varying from approximately 0.1 to 4.4 for a variety of different proteins (Cargile et al., 2004); in *Corynebacterium glutamicum* most proteins have a relative stable turnover in normal growth conditions but said stability is lost in response to specific events such as heat stress (Trötschel et al., 2012), or starvation, which has been shown to enhance proteolysis up to 30% h-1 of total intracellular protein (Trötschel et al., 2013); in *S. cerevisiae* and *S. pombe* nearly all proteins expressed under standard conditions were analyzed (4,425 and 3,705 proteins respectively) showing that turnover rates for all proteins range from a few minutes to more than 100 hr. in both species (Christiano et al., 2014; Mathieson et al ., 2018; Christiano et al., 2020).

In conclusion, all proteins are being continuously synthesized and degraded in all cells. Proteolysis represents the final destination of all proteins and although the continual destruction of proteins might seem wasteful, their turnover processes seem to represent the most extraordinary and essential dynamic phenomenon of the cell.

**2. Lipidome turnover**

The lipidome, the full lipid complement of cellular organisms, may comprise over 1,000 different molecular kinds for a single cell (Wenk, 2010) and 10,000-100,000 species for tissues or organisms, all of them originating from a few hundred individual lipid classes (van Meer et al., 2008; Yetukuri et al., 2008; Schwudke et al., 2011). Overall, they represent around 4-13 % dry weight of the cell, about 7-9% in *E. coli* 4-10% in *S. cerevisiae* and 13% in mammalian cells (Delgado et al., 2013).

In eukaryotic organisms, free fatty acids, the basic building blocks of most of the lipid compounds, are synthetized by fatty acid synthases and are broken down to generate mainly acetyl-coA through beta-oxidation process in the mitochondria. Omega-oxidation also occurs in the endoplasmic reticulum (Sanders et al., 2008). In yeasts and plants fatty acid oxidation is restricted to peroxisomes (Cooper, 2000). Moreover, a complex interplay between lipid metabolism and autophagy machinery has also been described (Xie et al., 2020). As a result of catabolic processes, in general, fatty acids exhibit high-rate turnover, for instance, palmitic acid, the most common of them found in animals, plants and microorganisms are characterized by oxidation rates around 1 μmol/min per gram dry weight in mouse heart cells (Banke et al., 2010).

Triacylglycerols, the major energy reserve in higher eukaryotes, are also in a dynamic state of flux inside the cell, resulting from a large cycle of lipolysis and re-esterification (Kalderon et al., 2000). Their lipolysis is initiated by the action of triacylglycerol lipases, that generate free fatty acids and diacylglycerol which can be hydrolyzed further into monoacylglycerol and glycerol. Certain lipases hydrolyze all acyl chains from the glycerol backbone, while others may act specifically on one kind of glycerolipid (Utsugi et al., 2009) resulting in a relative rapid turnover (Li et al., 2012). An alternative to triglyceride catabolism process through the lysosomal degradative pathway of autophagy has been described and termed lipophagy, by which triglycerides are taken up by autophagosomes and delivered to lysosomes for degradation by acidic hydrolases (Liu and Czaja, 2013).

In most mammalian cells, phospholipids account for approximately 60 mol% of total lipids (Han and Gross, 2005), which represent the major structural component in eukaryotic membranes (van Meer et al., 2008). Molecular degradation of phospholipids usually exhibits half-life on the order of minutes to several hours (Mudd, 1980), e. g., phosphorylcholine, the major membrane phospholipid, exhibits a half-life around 1 hour (Farber et al., 2000); phosphatidyl ethanolamine and phosphatidyl serine show relative half-lives around several hours in yeast cells (Gaigg et al., 2001); ether phospholipids as plasmanylcholine, plasmanylethanolamine, plasmenylethanolamine, and plasmenylcholine present apparent short half-lives about 36.5, 26.7, 23.1, and 15.1 min, respectively in rat cells (Rosenberger et al., 2002). Inositol phospholipids such as phosphatidylinositol, phosphatidylinositol-4-phosphate, and phosphatidylinositol-4,5-bisphosphate also show rapid relative turnover times with 21, 1.6, and 4.0 min, respectively, in canine trachealis muscle (Baron et al., 1989). Other experimental observations have shown that phosphatidylinositol-4,5-bisphosphate undergoes very rapid turnover with a half-life less than 40 s in beta cells (Thore et al., 2007).

Sterols are the major non-polar lipids of the cell and form a significant part of biological membranes. In red cells they have a half-life about 2 hours (Bretscher and Munro, 1993).

Glycolipids/sphingolipids represent approximately 10 mol% of total lipids (Han and Gross, 2005) and constitute another important class of structural molecules which are also in a dynamic state of synthesis and hydrolysis with rapid turnover, for instance, the ceramide of sphingolipids present a relative half-life about 2.7 h in cultured skin fibroblasts (van Echten-Deckert et al., 1997).

Even myelin glycerophospholipids, which are more metabolically stable than most other lipids, are continually removed, replaced and reactively transformed inside the cell (Morell and Ousley, 1994).

Metabolites such as acyl-CoA, lysolipids, acyl carnitines and others, that typically represent less than 5 mol% of total cellular lipids (Han and Gross, 2005) also may undergo rapid turnover, for instance, the time for turnover of the available acyl-CoA pool is of the order of seconds in mice heart cells (Banke et al., 2010).

Although the turnover of transcriptome and proteome of several eukaryotic model organisms have been described in detail, lipidome turnover remain relatively uncharacterized despite their importance. However, the numerous studies done to date suggest that the vast majority of lipids, if not all, are subject to molecular turnover.

The anabolism and catabolism of lipids are different in eukaryotes than prokaryotes, i.e., fatty acids are degraded by the classical ß-oxidation pathway in aerobically growing *E. coli*, but, in addition, they can also be utilized as anaerobic carbon sources involving regulatory mechanisms and enzymes largely distinct from responsible for aerobic catabolism of fatty acids (Campbell et al., 2003). Different works suggest that similar as in eukaryotic organisms, the prokaryotic lipids also may undergo very rapid turnover (Parsons and Rock 2013), for instance, fast degradation of fatty acids (Rock 1984), as well as phospholipids (Yokota and Kito 1982) and sterols (Wilbrink et al., 2011) have been observed in prokaryotic cells.

In short, lipids, as proteins, are critical to cellular function and it is generally accepted that lipid turnover is rapid and deregulation in turnover may results in disease (Dawidowicz 1987; Phillips et al., 2009; White and Ravussin, 2019).

**3. Glycome turnover**

Glycans are the most diverse set of biochemical molecules in cellular organisms (Kim et al. 2014). However, compared with proteome and lipidome, this type of biomolecules has been much less studied.

Carbohydrates or glycans besides being very diverse show often complex molecular configurations, and their cellular repertoire is estimated to be 10–10^4^ times larger than proteome and lipidome, depending on the species (Freeze, 2006).

Carbohydrates are found as monosaccharides or disaccharides, and complex glycans (mainly oligosaccharides, polysaccharides, glycolipids and glycoproteins). Different metabolic processes break down monosaccharides into smaller molecules and simple ions, e.g., glycolysis is the main degradative pathway which occurs in the cytosol of the cell in nearly all organisms, with variations (Romano and Conway, 1996). Pentose sugars may be metabolized through the pentose phosphate pathway, and the carbon skeletons may be converted into intermediates by different enzymatic processes as glycolytic/gluconeogenic routes (Kruger and von Schaewen, 2003). D-xylose may be catabolized in eukaryotic cells through the oxido-reductase and xylitol dehydrogenase pathways (Lee et al., 2012). Prokaryotes use oxidative processes (Weimberg, 1961; Dahms, 1974) and also a type of isomerase pathway (Karhumaa et al., 2007).

When monosaccharides are bound to the active sites of the enzymes, their half-lives are usually a small fraction of 1 s, e.g., under growing conditions, the turnover of cytosolic glucose in *Saccharomyces cerevisiae* can be extremely fast, at a rate of approximately 1 mM s^-1^ (de Koning et al, 1992). Monosaccharides can also be reactively transformed into larger molecules through glycosylation (the covalent addition of monosaccharides and other glycans) forming longer polymers, e.g., glycogen is accumulated in the stationary growth phase or under the presence of excess of carbon in both eukaryotic and prokaryotic cells, however, when it is needed for energy, this polysaccharide is broken down and converted again to glucose molecules in preparation for catabolism (Alonso-Casajús et al.,2006).

Polysaccharides are typically cleaved into smaller monosaccharaides mainly by glycoside hydrolases, polysaccharide lyases (or eliminases) and carbohydrate esterases (Levasseur et al., 2013), and the monosaccharide units can then enter into monosaccharide catabolism. Lytic polysaccharide monooxygenases (or LPMOs) exhibit copper-dependent oxidation to cleave glycosidic bonds in polysaccharides (Harris et al., 2010); Vaaje-Kolstad et al., 2010); Levasseur et al., 2013; Kim et al., 2014). Polysaccharide turnover can be found in all Phyla of prokaryotes (Seibold et al., 2007) and eukaryotes (Bergans eta al., 2000; Brandão et al., 2009).

Another set of glycans correspond to glycoproteins which are essential complex molecules for specific cell functionalities. Approximately half the proteins are glycosylated in eukaryotic cells (Apweiler et al., 1999; Khoury et al., 2011; Kung et al., 2009). Glycosylation of proteins is one of the most complicated posttranslational modifications that a protein can undergo (Spiro, 2002). This enzymatic process occurs throughout the entire phylogenetic spectrum, ranging from archaea and eubacteria to eukaryotes (Spiro, 2002). As is the case of other cellular biomolecules, normal functioning of the cell is characterized by continual formation and degradation of glycoproteins at different rates (Winchester, 2005). Carbohydrates are added to proteins in very complex processes which involve two organelles, the endoplasmic reticulum and the Golgi apparatus. Once formed, glycoproteins are transported to their specific location, and similar to other biomolecules, after a period of time undergo molecular destruction. For catabolism, they are delivered to lysosomes either by endocytosis or by autophagy, and finally all the digestion products are recycled (Winchester, 2005; Suzuki, 2009).

Inside the lysosome, glycoproteins are broken down by a combination of proteases (endopeptidases and exopeptidases) and glycosidases (which breakdown the N- and O-linked glycans). Amino acids, dipeptides and monosaccharides are transported across the lysosomal membrane into the cytosol by a combination of carrier-mediated transport and diffusion processes (Hart and Akimoto, 2009; Winchester, 2005).

Experimental observations have shown that the carbohydrate moieties are degraded faster than the protein portion of the glycoproteins (Tauber et al., 1983). For instance, in several plasma membrane glycoproteins from rat liver the protein moieties exhibited half-lives ranging from 70 to 78 h and the terminal sugars, L-fucose, and N-acetylneuraminic acid and D-galactose presented significantly shorter half-lives, averaging 12.5 h, 33 h and 20 h respectively (Kreisel et al., 1980).

It has been suggested that basal autophagy is required for proper degradation of glycoproteins in the lysosomes. In this sense, the relationship between autophagy and glycan catabolism appears to be specific to sialyl glycans originating from N-glycans (Seino et al., 2013). Incorrectly glycosylated proteins are also broken down in the ER and cytoplasm, and the end fragments of the degradation of glycans are delivered to lysosomes (Cacan and Verbert, 1997; Suzuki et al., 1994). Proteasome and the lysosome also seem to work in conjunction in the breakdown of the incorrectly folded glycoproteins (Hirsch et al., 2003; Suzuki and Lennarz, 2003; de Virgilio et al., 1998)

Alternative degradation for N-glycans and other complex-type glycans through non-lysosomal catabolic processes in the cytosol, also seem to occur but these pathways remain unclear (Suzuki, 2007; Ohashi et al., 1999; Ishizuka et al., 2008). Protein glycosylation plays important roles not only in eukaryotes but also in prokaryotes (Zhou, 2009; Wang et al., 2012). Although the metabolism of glycoproteins in prokaryotes has been little studied, nowadays, more than 70 bacterial glycoproteins have been reported (Zhou, 2009), and different processes of glycoprotein catabolism have been described (Suvorov et al., 2008; Park, 2001).

Glycolipids are a large and heterogeneous family of cellular molecules that form complex patterns in all species, ranging from bacteria to humans (Malhotra, 2012; Nelson and Cox, 2008). Although a plethora of important structural and cellular functions has been assigned to different classes of glycolipids (Sonnino et al., 2009; Prinetti et al., 2009; Kolter, 2011) the explicit role of these complex molecules in cells is largely unclear.

An important part of the glycolipids is located in the outer leaflet of the plasma membrane; however, experimental evidence indicates that in eukaryotic cells glycolipids are also distributed in membranous structures of intracellular organelles, such as Golgi apparatus, endosomes, lysosomes, nuclear membrane, endoplasmic reticulum, and mitochondria (Ardail et al., 2003; Gillard et al., 1993; Malhotra, 2012).

Just like glycoproteins, glycolipids undergo permanent cycles for metabolic turnover in cell. In eukaryotic cells, they are biosynthesized in endoplasmic reticulum and Golgi apparatus, transported and deposited at their specific location, fulfill their function for some period of time, and then undergo molecular catabolic degradation (Aureli et al., 2011; Kolter, 2005, 2011; Sandhoff and Kolter, 2003; Tettamanti, 2003). For instance, gangliosides, the most complex glycolipids, are continuously synthesized and degraded in cells exhibiting different metabolic turnover rates with half-life values which can range from 2 to 6.5 h for up to 3 days depending on the cells used in the experiments (Tettamanti, 2003). Constitutive glycolipid degradation occurs in the acidic compartments of the cells, the endosomes and the lysosomes (Furst and Sandhoff, 1992; Sandhoff and Kolter, 1996; Van der Goot and Gruenberg, 2002). Lysosomal glycosidases sequentially break down the sugar residues from the non-reducing end of their glycolipid substrates, and other catalytic enzymes degrade the part non-glycosylated of the glycolipid. The resulting monosaccharides, sialic acids, fatty acids, sphingoid bases, etc., can leave the lysosome, they are then available for reuse or are further degraded. (Tettamanti, 2004; Sandhoff and Kolter, 2003).

**4. Transcriptome turnover**

Like the proteome, lipidome and glycome, the transcriptome, the total set of transcripts in a given cell, is highly dynamic, exhibiting a permanent molecular turnover. All cellular transcripts including mRNA, rRNA, tRNA and noncoding small RNAs are continually synthesized and destroyed through complex regulatory metabolic processes.

Transcriptome ranges from hundreds of thousands of molecules in prokaryotes to millions in eukaryotic cells, representing around 4-20 % of dry weight of cells (approximately 20% in *E. coli,* 6-12 % in *S. cerevisiae* and 4% in mammalian cell) (Delgado et al., 2013).

There are four main types of RNA: messenger RNA, transfer RNA, ribosomal RNA and small RNAs. mRNAs are translated into proteins, whereas tRNAs and rRNAs have housekeeping roles during mRNA translation, and small RNAs are currently being recognized as an important class of regulatory noncoding transcripts that are implicated in a variety of biological functions.

Messenger RNA (mRNA) is a large family of RNA molecules, including thousands of individual species that can be simultaneously contained in a single cell, i.e. in proliferating cells global mRNA abundance has been estimated to be of about ∼41,000 molecules/cell on average for yeast populations (Marguerat et al., 2012). mRNA molecules are synthesized by specific RNA polymerases and destroyed by nucleases. This degradation process is universal and occurs at a relatively high rate, exhibiting half-lives from minutes to hours (Meyer et al., 2004). Specifically, numerous studies have measured the decay rates of mRNAs in different cellular species. For instance, in HeLa Tet-Off cells the half-life for 11,052 mRNAs varied between 40 min and ∼9 h, with an average half-life of ∼6.9 h (Tani et al., 2012). Decay rates measured for 19,977 non-redundant gene expressions from mouse embryonic stem cells showed an average half-life of ∼7.1 h, and around 100 genes exhibited half-lives of less than 1 h (Sharova et al., 2009). In other studies of thousands of mRNAs belonging to different kinds of human cells, the average half-life was around 10 h and the short half-lives were less than 2 h (Yang et al., 2003). Dynamic transcriptome analyses in yeast (15,000 mRNA per cell studied) have shown that most half-lives range around a median of 11 min, this rapid transcript turnover does not correlate with other published rates, which seem to be obtained with protocols that may perturb certain metabolic processes (Miller et al., 2011). In other studies, the short mRNA half-lives are approximately 2–3 min while the longer stable mRNAs have half-lives of 90 min or more (Herrick et al., 1990; Wang et al., 2002a). Quantitative dynamic studies in *E. coli* have shown a rapid transcript turnover; in these studies, the more unstable mRNAs have half-lives of approximately 1–2 min, whereas the larger half-lives are about 15 min (Coburn and Mackie, 1999). In general, mRNAs with the shortest half‐lives are involved in the regulation of transcription and the cell cycle and mRNA processing. More concretely, mRNAs that correspond to proteins which are required for a limited time in cellular metabolic processes such as regulation of the cell cycle, post-translational modifications, cellular growth, oncogene regulation, cell differentiation or responses to external stimuli, often have short half-lives (Schoenberg and Maquat, 2012; Tani et al., 2012; Pérez-Ortín et al., 2013).

Moreover, the decay rates of specific mRNAs are affected by a wide variety of factors (Wang et al., 2002), including specific hormones (Ross, 1996), iron (Casey et al., 1988), viral infection (Feng et al., 2001), environmental changes (Schoenberg and Maquat, 2012), cell cycle progression (Morris et al., 1991), cell differentiation (Jäck and Wabl, 1988) and molecular interactions with AREs (AU-rich elements) (Shen and Malter, 2015). Notice that, although synthesis rates correlated well with mRNA levels, decay rates did not correlate with synthesis rates. This seems to indicate that mRNA destruction and synthesis are functionally independent during normal growth, and both processes contribute to setting cellular mRNA levels. (Miller et al., 2011; Schwanhäusser et al., 2011).

Ribosomal RNAs represent a large fraction of transcriptome mass. For instance, in exponentially proliferating yeast cells an average number of ∼800,000 rRNAs/cell has been quantified (Marguerat et al., 2012), forming around 187,000 (+-56,000) ribosomes depending on the specific cellular conditions (von der Haar, 2008). An average bacterium such as *E. coli* has approximately 55,000 ribosomes (Bakshi et al., 2012), whereas a human liver cell has 13 million on the rough endoplasmic reticulum alone (Weibel et al., 1969).

While the half-life of precursor rRNA is very short, no longer than a few minutes in human cells (Slomovic et al., 2006), the mature molecules are among the longest-lived observed RNAs, exhibiting half-lives of 3 days in human fibroblasts (Gillery et al., 1995), 3.8 days in H1299 cells (Yi et al., 1999) and about 7.5 days in cultured rat fibroblasts (Halle et al., 1997). Relatively short half-lives of mitochondrial RNAs, of about 2.5 to 3.5 h, have been observed in HeLa cells for the two main ribosomal RNA components (Gelfand and Attardi, 1981). In mutants of *E. coli*, short rRNAs’ half-lives have been determined to be of a few minutes (Schäferkordt and Wagner, 2001). However, in other wild type (non-mutant) prokaryotes, such as the archaeon *Natronococcus occultus,* the half-life of rRNAs were found to be longer, of 11.43, 14.85, 5.28 and 7.14 h during the initial, exponential, late exponential and stationary growth respectively (Nercessian and Conde, 2006).

Despite ribosomal RNAs are exhibiting long decay rates during exponential growth, under certain physiological conditions, such as changes in the nutrient environment, extensive and rapid degradation may occur (Deutscher, 2003). In fact, it has been known for many years that during starvation the cell rapidly adjusts the size of the translation machinery in response, and thus the rRNAs are extensively degraded (Deutscher, 2003), amounting to >95% degradation of ribosomes (Ramagopal, 1984). Mature rRNAs that are defective for translation are subjected to surveillance by a complex rRNA degradation pathway (LaRiviere et al., 2001; Cole et al., 2009; Fujii et al., 2009).

Transfer RNAs (tRNAs) are essential molecules for protein synthesis whose main role is to deliver amino acids as specified by the messenger RNA codons. On average, bacterium *E. coli* has approximately 375,000 tRNA molecules per cell (Mackie, 2012), whereas single *Saccharomyces cerevisiae* cell growing on glucose contains ∼3,300,000 molecules (Waldron and Lacroute, 1975).

tRNAs are synthetized by a DNA-dependent RNA polymerase as precursor molecules (pre-tRNA) that, after transcription, undergo a bewildering number of reactive post-transcriptional alterations, which in yeast include nucleotide removal, nucleotide addition and nucleoside modifications such as sugar methylations, aminoacylations, base deaminations, and base isomerizations, between them (Hopper, 2013). About 100 tRNA modifications have been described, many of which are highly conserved among different organisms (Chernyakov et al., 2008). In fact, in all organisms tRNAs are highly modified (El Yacoubi et al., 2012), and complex splicing transformations have been observed in Archaea, vertebrates (Popow et al., 2011, 2012) and plants (Gegenheimer et al., 1983; Englert and Beier, 2005). Similarly to other RNAs, different stress conditions can also result in changes in tRNA modification (Kamenski et al., 2007; Chan et al., 2012).

In general tRNAs show relatively long half-lives, estimated to be in the order of hours and even days (Anderson et al., 1998; Phizicky and Hopper, 2010; Gudipati et al., 2012). Very long decay rates have been observed in *Euglena gracilis*, of 44 h (Karnahl and Wasternack, 1992), in chicken muscle, of 50 h (Nwagwu and Nana, 1980), and in avian liver, of 72h (Kanerva and Maenpaa, 1981). During its lifetime each tRNA goes through the translation cycle ∼40 times per minute (Waldron and Lacroute, 1975).

Mature tRNA species lacking certain modifications are subject to rapid tRNA degradation (Alexandrov et al., 2006; Chernyakov et al., 2008; Kotelawala et al., 2008). In *Saccharomyces cerevisiae*, hypomodified tRNAs are also degraded via a rapid decay pathway, with a half-life of the order of minutes. As expected, the turnover of individual aminoacyl-tRNA families in *E. coli* cells was estimated to be very fast, in the range of 1.7 to 8.1 s^-1^ with a mean value of 3.7 s^-1^ (Jakubowski and Goldman, 1984).

Small RNAs, are emerging as an important class of regulatory transcripts implicated in a variety of biological functions often involved in regulating translation of target RNAs through RNA–RNA interactions (Atkinson et al., 2011; Tani et al., 2012). They are not translated into proteins and can be roughly classified into three main groups: microRNAs (miRNAs), small interfering RNAs (siRNAs) and Piwi-associated RNAs (piRNAs) (Grosshans and Filipowicz, 2008). Recent transcriptomic studies suggest the existence of thousands of small RNAs in the cell.

MicroRNAs (miRNAs) constitute a large class of regulatory non-coding RNAs that repress target messenger RNAs controlling both transcriptional and post-transcriptional levels (Chatterjee and Grosshans, 2009). miRNAs are typically transcribed by RNA polymerase II as primary transcripts that are subsequently matured in a multi-step biogenesis process to generate mature, functional form. A family of exoribonucleases are the main enzymes that catalyze the breakdown of phosphodiester bonds in miRNAs (Ramachandran et al., 2008). The majority of miRNAs seem to be stably expressed (Bail et al., 2010; Krol et al., 2010). However, decay rates measured for 1418 small RNAs in HeLa Tet-off (TO) showed heterogeneity in their lifespan, with an average half-life of ∼7.0 h, the most unstable exhibited relatively short half-lives with t1/2 < 4 h , whereas larger half-lives, exhibited typically by miRNAs with housekeeping functions, are about t1/2 ≥ 4 h (Tani, et al., 2012).

Other studies have shown that miRNAs can display relative fast decay dynamics in different types of cells and biological conditions (Zhang et al., 2012; Rüegger and Großhans, 2012; Krol et al., 2010). For instance, catabolism of microRNAs reveals a rapid turnover in neurons (Rajasethupathy et al., 2009; Sethi and Lukiw, 2009; Krol et al., 2010).

Small noncoding RNAs also function as regulators of gene expression during numerous physiological processes in prokaryotic cells (Gottesman, 2005; Gottesman and Storz, 2011; Richards and Vanderpool, 2011; Storz et al., 2011; Mank et al., 2012) and, as in eukaryotic cells small RNAs exhibit degradation processes (Andrade and Arraiano, 2008; Göpel et al., 2013). For instance, in *E. coli* it has been observed that polynucleotide phosphorylase (PNPase) is the major factor involved in the rapid degradation of small RNAs, especially those that are free of binding to chaperone Hfq (Andrade et al., 2012). In general, the turnover processes of small RNAs both in prokaryotes and eukaryotes are still not well understood.

**5. Molecular transformation of the cellular substructures**

All cells are complex metabolic reactors for molecular transformations, in permanent status of synthesis and destruction. The structures that shape the cells permanently suffer complex chemical transformations. Nothing is inert in unicellular organisms. No molecule and molecular structure escapes the change and transformation processes that take place within the basic units of life.

**5.1 Cytoplasmic membrane.**

The cytoplasmic membrane, the most thoroughly studied of all cell membranes, exhibits several mechanisms for recycling extensive portions of its molecular structure (Okuda and Eiraku, 2017). The most well understood is the endocytic process which is an energy-using mechanism conserved in all eukaryotic cells (Maxfield and McGraw, 2004). During endocytosis, the membrane internalization rate is very fast, with a half-time of only a few minutes (Hao and Maxfield, 2000). The direct return of some proteins and lipids to the cell surface has been reported to have a half-time of about 6 minutes (Sheff et al., 1999). With similar kinetic times of t1/2 about 10 minutes, transferrin receptors, C6-NBD-sphingomyelin and other constitutively recycled molecules return to the cytoplasmic membrane from endocytic recycling compartment (Mayor et al., 1993). Other experiments have shown that cytoplasmic membrane recycling from the pinosomal compartment occurs after an average residence time of about 3 min in macrophages and 4-6 min in fibroblasts (Thilom, 1985). This rapid turnover of the cytoplasmic membrane allowed to suggest that an area equivalent to the whole plasma membrane is approximately internalized once every 30 min in macrophages and amoebae (Thilom 1985). Similar results have been obtained in more recent studies with macrophages (Rastogi 2010) where in 30 minutes a macrophage endocytose an amount of plasma membrane equal to its complete plasma membrane.

**5.2 Cytoskeleton.**

Cytoskeleton is a molecular dynamic system which undergoes continuous dynamic reorganization along the cell cycle. Three types of cytoskeletal filaments are common to many eukaryotic cells: microfilaments, intermediate filaments and microtubules.

Microfilaments, the thinnest filaments of the cytoskeleton, are composed of linear polymers of actin subunits exhibiting fast turnover of molecular assembly and disassembly in the order of seconds (Theriot and Mitchison, 1991), individual filaments persisting for only a few minutes (Henty et al., 2011). Like actin, intermediate filaments (IF) are dynamic molecular structures, for example, IF vimentin in fibroblasts, a common structural support of many cells, forms networks that continuously change their organization with a half-time of 5–14 min (Vikstrom et al., 1992; Yoon et al., 1998). Mitotic microtubules can depolymerize and repolymerize rapidly showing a half-time of 10–20 s (Salmon et al., 1984) and in interphase microtubule recover much more slowly, about 200s (Saxton et al., 1984).

Bacteria also contain dynamic cytoskeletal structures, analogous to the eukaryotic cytoskeletal proteins (Graumann, 2009). FtsZ, a homolog of tubulin, is the major cytoskeletal component of the bacterial division machinery which forms a ring-shaped structure, called the Z ring that constricts as the bacterium divides. *E. coli* Z-ring is extremely dynamic, continually remodeling itself with a half-time of 30 s (Anderson et al., 2004).

Prokaryotic actin-like proteins, such as MreB, ParM, and MamK, are involved in the maintenance of cell shape, forming highly dynamic filamentous structures, i.e., MreB shows a high turnover remodeling its molecular organization on a time scale of tens of seconds (Reimold et al., 2013). Crescentin, an intermediate filament protein discovered in *Caulobacter crescentus*, organizes into extended structures that exhibit a half time of recovery about 26 min (Esue et al., 2010).

**5.3 Mitochondria.**

Mitochondria are dynamic organelles that are constantly undergoing fusion, fission and molecular destruction. Under cellular conditions, mitochondria are maintained via permanent cycles of degradation and biogenesis. Mitophagy is the selective degradation of mitochondria by autophagy which is not limited to the damaged mitochondria but also involves undamaged ones (Youle and Narendra, 2011). It has been estimated that liver mitochondria exhibit a half-life about 1.83 days (Miwa et al., 2008). Rat cardiomyocytes have roughly 1000 mitochondria per cell, suggesting that, under basal resting conditions, one mitochondrion per cell is replaced every 40 min (Stotland and Gottlieb, 2015). In the cultured mouse atrial cell line HL-1 subjected to nutrient deprivation, experimental observations have shown that the number of mitochondria can be reduced by 70% within 3.5 h (Carreira et al., 2010).

**5.4 Other cellular organelles.**

Organelles are specialized structures within the cell that carry out specific and essential functions for its functionality and survival. Cells modulate the number of organelles or degrade parts of them according to environmental conditions, systemic metabolic dynamics and energetic needs. The overall organelle composition and number are tightly regulated in the cell (Anding and Baehrecke, 2017).

The main molecular destruction system of cytoplasmic organelles is autophagy, which involves the sequestration of components of the cytoplasm into double-membrane vesicles called autophagosomes. These vesicles then fuse with lysosomes (vacuoles in yeast and plants) to degrade their contents (Mizushima et al., 2002; Anding and Baehrecke, 2017). In fact, autophagy represents the major intracellular pathway for the degradation and recycling of long-lived proteins and cytoplasmic organelles (Kelekar, 2005). Autophagy encompasses all processes delivering portions of cytosol and organelles to the lysosome for macromolecule turnover and recycling of building blocks, however, some organelles or parts of them can be removed by selective molecular degrading systems or otherwise unselective macroautophagy and microautophagy (Farré et al., 2009). About 30% of cytosolic proteins can be degraded by chaperone-mediated autophagy, and it plays an important part in the regulation of protein degradation under different physiological conditions (Guillaume et al., 2019). Peroxisomal turnover also takes place in eukaryotic cells by autophagy-related mechanism. Studies of peroxisome degradation in mammalian cells indicated that they have a half-life of approximately 2 days under basal metabolic conditions (Ezaki et al., 2011).

The endoplasmic reticulum (ER), the largest intracellular endomembrane system, also exhibit constant turnover by a process termed ER-phagy (Schuck et al., 2014; Khaminets et al., 2015). In experiments performed in rat hepatocytes five decades ago the total membrane proteins of ER exhibited half-lives of from 75 to 113 hours, while in the same studies, total membrane lipids have 10 to 30% shorter half-lives (Omura et al., 1967). As in the ER, the Golgi apparatus, flattened membrane cisternae that are usually organized into stacks, exhibits continuous turnover (Mogelsvang et al., 2003). Peroxisomal turnover also takes place in eukaryotic cells by autophagy-related mechanism. Studies of peroxisome degradation in mammalian cells indicated that they have a half-life of approximately 2 days under basal metabolic conditions (Ezaki et al., 2011).

In eukaryotic cells, the nucleus is bounded by a double-membrane which undergoes drastic reorganization, including its complete destruction, during major cellular events such as cell division and apoptosis. Whereas the outer membrane is an extension of the endoplasmic reticulum, the inner nuclear membrane contains many specific proteins that are involved in different nuclear processes during the interphase, e.g., it has been reported the presence of specific protein degradation in the inner phospholipid bilayer by the proteasome e.g., the protein Asi2 exhibits a half-life of about 45 min in yeast (Boban et al., 2014). Evidence in the yeast *Saccharomyces cerevisiae* has shown that nucleus derived material can be specifically degraded by a selective form of autophagic process termed nucleophagy (Mijaljica and Devenish, 2013).

**5.5 DNA.**

DNA is not an inert macromolecule, but rather a highly dynamic and reactive molecular structure able to interact in many ways with its physical-chemical environment, and as a consequence of these interactions, DNA is permanently changing its chemical composition.

In eukaryotic cells, the chief structural proteins that bind DNA are histones, the foundation blocks of chromatin, and distinct multiprotein complexes can chemically modify them, resulting that certain DNA sequences are exposed or occluded. There are two major classes of chromatin-modifying complexes, ATP-dependent remodeling complexes and histone-modifying complexes, which are coordinated to regulate some functions of the transcription machinery. ATP-dependent chromatin-remodeling complexes use ATP hydrolysis to increase the accessibility of nucleosomal DNA and regulate gene expression by either moving, ejecting or restructuring nucleosomes (Narlikar et al., 2002). The other class of complexes, which covalently modify nucleosomes, can add or remove many chemical moieties in the processes of acetylation, deacetylation, phosphorylation, methylation, ubiquitylation, sumoylation, deimination, ADP ribosylation and proline isomerization among others (Bannister and Kouzarides, 2011; Tan et al., 2011). These chemical modifications of the histones may result in altered gene expression but no changes in the DNA sequence are produced.

Prokaryotic cells, despite exhibiting a different organization of their DNA than that of the eukaryotes, also present a protein collectivity referred to as nucleoid-associated proteins, which contribute to the compaction and regulation of the genome (Rimsky and Travers, 2011; Takeda et al., 2011), involving a great variety of protein interactions (Dame, 2005).

Moreover, topological changes are constantly generated by DNA tracking processes which must constantly be removed by DNA topoisomerases (Champoux, 2001). These isomerase enzymes are ubiquitous proteins, found in all cell types, both prokaryotic and eukaryotic cells, which act regulating DNA supercoiling, catalyzing the winding and unwinding of DNA strands. They bind to either single-stranded or double-stranded nucleic acids and cut the phosphate backbone, which allow swiveling and relaxing/coiling the DNA before repair the cuts. Catalytic changes generating by topoisomerases are required for many processes involving DNA, such as DNA replication and transcription, and therefore play an important role during expression of genes (Pedersen et al., 2012).

One of the most intensively studied molecules that interact with particular DNA sequences are transcription factors. They are proteins that contain one or more DNA-binding domains which attach to specific sequences of DNA contributing to regulate the gene expression (Mitchell and Tjian, 1989). Transcription factors carry out their activity in different ways, e. g., they bind the RNA polymerase responsible for transcription, either directly or through other mediator proteins (Myers and Kornberg, 2000), and alternatively, they also can bind to enzymes that chemically modify the histones (Spiegelman and Heinrich, 2004), activating different catalytic activities such as histone acetyltransferase activity (Narlikar et al., 2002) and histone deacetylase activity (Xu et al., 1999).

Furthermore, DNA is a macromolecule constantly subjected to damage by a variety of factors which can alter its chemical composition. Base modifications are one the most common type of endogenous DNA damage, accounting for thousands of lesions per mammalian genome per day (De Bont and Larebeke, 2004). Besides, frequently, hydrolysis, oxidation, misincorporated nucleotides and nonenzymatic methylation of DNA also occur at significant rates in vivo (Lindahl, 1993; De Bont and van Larebeke, 2004). Eukaryotic and prokaryotic cells possess multiple mechanisms to repair DNA which is achieved by specific enzyme activity and different metabolic pathways. These include base excision repair (Kim and Wilson, 2012), nucleotide excision repair (Marteijn et al., 2014) and other DNA repair mechanisms, which are regulated by complex metabolic networks, extensive post-translational modifications, and multiple dynamic chromatin interactions (Marteijn et al., 2014).

In general, major processes of DNA metabolism such as repair, replication and recombination, require translocation of enzymes along the nucleic acid. Enzymes involved in these processes, particularly are polymerases and helicases which break hydrogen bonds between bases and unwind the DNA double helix into single strands. Nucleases, ligases, and other biomolecules such as mismatch repair proteins (Qiu et al., 2015), the Mre11/Rad50/Nbs1 complex (Yuan and Chen, 2010), the DNA-PKcs kinases and the DNA-PKcs/Ku70/Ku80 complex (Spagnolo et al., 2006) also play essential role in the repair of DNA double-strand breaks.

In addition to the large repertory of molecular interactions on genome, a wide variety of experimental approaches have provided evidence that the DNA of most species exhibits methylation-demethylation dynamics on specific nucleotides altering reversibly its physical structure. The most prominent of these covalent chemical modifications is the methylation of the carbon-5 of cytosine (5mC), the classical epigenetic mark 5-methylcytosine, however other DNA modifications such as 5-hydroxymethylcytosine (5hmC) and N6-methyladenine (6mA) have also been observed (Breiling and Lyko, 2015). Mammals show a dense methylation at CpG sites, >70% (Smith and Meissner, 2013) and the reversible dynamics of some of these covalent marks can occur in a matter of minutes (Barrès et al., 2012). Epigenetic methylation patterns have also been observed in bacteria (Casadesús and Low, 2013; Gonzalez et al., 2014).

All functions of the deoxyribonucleic acid depend on molecular interactions with DNA-associated metabolism (De la Fuente, 2015), and as a consequence of these reactive interactions, DNA is permanently changing its chemical composition.

Like all molecules that shape the cell, DNA undergoes dramatic molecular changes along the cell cycle, exhibiting incessant reactive dynamics; but the particularity of these molecular transformations is that they practically do not affect the information contained in the nucleotide sequences. There appears to be a functional invariance in metabolic processes that governs the molecular changes in the deoxyribonucleic acid, i.e., DNA undergoes incessant reactive transformations but retains the information contained in the nucleotide sequences. This invariance seems to be essential for the maintenance of *Cellular Metabolic Structure* (De la Fuente, 2015).

**References**

Alexandrov, A., Chernyakov, I., Gu, W., Hiley, S.L., Hughes, T.R., Grayhack, E.J., et al. (2006). Rapid tRNA decay can result from lack of nonessential modifications. *Mol. Cell* 21, 87–96. doi: 10.1016/j.molcel.2005.10.036

Alonso-Casajús, N., Dauvillée, D., Viale, A.M., Muñoz, F.J., Baroja-Fernández, E., Morán-Zorzano, M.T., et al. (2006). Glycogen phosphorylase, the product of the glgP gene, catalyzes glycogen breakdown by removing glucose units from the nonreducing ends in Escherichia coli. *J. Bacteriol.* 188, 5266–5272. doi: 10.1128/JB.01566-05

Anderson, D.E., Gueiros-Filho, F.J., and Erickson, H.P. (2004). Assembly dynamics of FtsZ rings in Bacillus subtilis and Escherichia coli and effects of FtsZ-regulating proteins. *J. Bacteriol.* 186, 5775-5781. doi: 10.1128/JB.186.17.5775-5781.2004

Anderson, J., Phan, L., Cuesta, R., Carlson, B.A., Pak, M., et al. (1998). The essential Gcd10p-Gcd14p nuclear complex is required for 1-methyladenosine modification and maturation of initiator methionyl-tRNA. *Genes Dev.* 12, 3650–3662. doi: 10.1101/gad.12.23.3650

Anding, A.L., and Baehrecke, E.H. 2017. Cleaning house: selective autophagy of organelles. *Dev. Cell.* 41, 10-22. doi: 10.1016/j.devcel.2017.02.016

Andrade, J.M., and Arraiano, C.M. (2008). PNPase is a key player in the regulation of small RNAs that control the expression of outer membrane proteins. *RNA*. 14, 543-551. doi: 10.1261/rna.683308

Andrade, J.M., Pobre, V., Matos, A.M., and Arraiano, C.M. (2012). The crucial role of PNPase in the degradation of small RNAs that are not associated with Hfq. *RNA* 18, 844-55. doi: 10.1261/rna.029413.111

Apweiler, R., Hermjakob, H., and Sharon, N. (1999). On the frequency of protein glycosylation, as deduced from analysis of the SWISS-PROT database. *Biochim. Biophys. Acta* 1473, 4–8. doi: 10.1016/s0304-4165(99)00165-8

Ardail, D., Popa, I., Bodennec, J., Louisot, P., Schmitt, D., and Portoukalian, J. (2003). The mitochondria-associated endoplasmic-reticulum subcompartment (MAM fraction) of rat liver contains highly active sphingolipid-specific glycosyltransferases. *Biochem. J.* 371(Pt 3):1013-1019. doi: 10.1042/BJ20021834

Atkinson, S.R., Marguerat, S., and Bähler J. (2011). Exploring long non-coding RNAs through sequencing. *Semin. Cell. Dev. Biol*. (2012). 23, 200-205. doi: 10.1016/j.semcdb.2011.12.003.

Aureli, M., Loberto, N., Chigorno, V., Prinetti, A., and Sonnino, S. (2011). Remodeling of sphingolipids by plasma membrane associated enzymes. *Neurochem. Res.* 36, 1636-1644. doi: 10.1007/s11064-010-0360-7

Bail, S., Swerdel, M., Liu, H., Jiao, X., Goff, L.A., Hart, R.P., et al. (2010). Differential regulation of microRNA stability. *RNA* 16, 1032-1039. doi: 10.1261/rna.1851510

Bakshi, S., Siryaporn, A., Goulian, M., and Weisshaar, J.C. (2012). Superresolution imaging of ribosomes and RNA polymerase in live Escherichia coli cells. *Mol. Microbiol.* 85, 21-38. doi: 10.1111/j.1365-2958.2012.08081.x

Banke, N.H., Wende, A.R., Leone, T.C., O'Donnell, J.M., Abel, E.D., Kelly, D.P., et al. (2010). Preferential oxidation of triacylglyceride-derived fatty acids in heart is augmented by the nuclear receptor PPARalpha. *Circ. Res.* 107, 233-241. doi: 10.1161/CIRCRESAHA.110.221713.

Bannister, A.J., and Kouzarides, T. (2011). Regulation of chromatin by histone modifications. *Cell Res.* 21, 381-395. doi: 10.1038/cr.2011.22.

Baron C. B., Pring M. and Coburn R. F. (1989). Inositol lipid turnover and compartmentation in canine trachealis smooth muscle. *Am. J. Physiol.* 256, C375-C383. doi: 10.1152/ajpcell.1989.256.2.C375

Barrès, R., Yan, J., Egan, B., Treebak, J.T., Rasmussen, M., Fritz, T., et al. (2012). Acute exercise remodels promoter methylation in human skeletal muscle. *Cell. Metab.* 15, 405-411. doi: 10.1016/j.cmet.2012.01.001

Battesti, A., and Gottesman, S. (2013). Roles of adaptor proteins in regulation of bacterial proteolysis. *Curr. Opin. Microbiol.* 16, 140-147. doi: 10.1016/j.mib.2013.01.002.

Batth, T. S., Francavilla, C., and Olsen, J.V. (2014). Off-line high-pH reversed-phase fractionation for in-depth phosphoproteomics. *J. Proteome Res.* 13, 6176–6186. doi: 10.1021/pr500893m

Belle, A., Tanay, A., Bitincka, L., Shamir, R., and O’Shea, E. K. (2006). Quantification of protein half-lives in the budding yeast proteome. *Proc. Natl. Acad. Sci. U.S.A.* 103, 13004–13009. doi: 10.1073/pnas.0605420103

Bergans, N., Stalmans, W., Goldmann, S., and Vanstapel, F. Molecular mode of inhibition of glycogenolysis in rat liver by the dihydropyridine derivative, BAY R3401: inhibition and inactivation of glycogen phosphorylase by an activated metabolite. *Diabetes*. (2000). 49, 1419-26. doi: 10.2337/diabetes.49.9.1419

Boban, M., Pantazopoulou, M., Schick, A., Ljungdahl P.O., and Foisner, R. (2014). A nuclear ubiquitin-proteasome pathway targets the inner nuclear membrane protein Asi2 for degradation. *J. Cell Sci.* 127(Pt 16):3603-3613. doi: 10.1242/jcs.153163

Boisvert, F.M. (2012). A quantitative spatial proteomics analysis of proteome turnover in human cells. *Mol. Cell. Proteom.* 11:M111.011429. doi: 10.1074/mcp.M111.011429.

Boubakri, H., Seghezzi, N., Duchateau, M., Gominet, M., Kofroňová, O., Benada, O., et al. (2015). Absence of pupylation (prokaryotic ubiquitin-like protein modification) affects morphological and physiological differentiation in Streptomyces coelicolor. *J Bacteriol.* 197, 3388-3399. doi: 10.1128/JB.00591-15

Brandão, A.D., Del Bem, L.E., Vincentz, M., and Buckeridge, M.S. (2009). Expression pattern of four storage xyloglucan mobilization-related genes during seedling development of the rain forest tree Hymenaea courbaril L. *J. Exp. Bot.* 60, 1191-1206. doi: 10.1093/jxb/erp014

Breiling, A., and Lyko, F. (2015). Epigenetic regulatory functions of DNA modifications: 5-methylcytosine and beyond. *Epigenetics Chromatin.* 8:24. doi: 10.1186/s13072-015-0016-6.

Bretscher, M.S., and Munro, S. (1993). Cholesterol and the Golgi apparatus. *Science* 261, 1280-1281. doi: 10.1126/science.8362242

Buckeridge, M.S., Tiné, M.A.S., and Dos Santos H.P. (2000). Mobilisation of storage cell wall polysaccharides in seeds. *Plant Physiology and Biochemistry*. 38, 141–156. doi: 10.1016/S0981-9428(00)00162-5

Cacan, R, and Verbert, A. (1997). Free oligomannosides produced during the N-glycosylation process: origin, intracellular trafficking and putative roles. *TIGG* 9, 365–377. doi: 10.4052/tigg.9.365

Cain, J.A., Solis, N., and Cordwell, S.J. (2014). Beyond gene expression: the impact of protein post-translational modifications in bacteria. *J. Proteomics* 97, 265-86. doi: 10.1016/j.jprot.2013.08.012.

Campbell, J.W., Morgan-Kiss, R.M., and Cronan, J.E. Jr. (2003). A new Escherichia coli metabolic competency: growth on fatty acids by a novel anaerobic-oxidation pathway. *Mol. Microbiol*. 47, 793–805. doi: 10.1046/j.1365-2958.2003.03341.x

Cargile, B.J., Bundy, J.L., Grunden, A.M., and Stephenson J.L. Jr. (2004). Synthesis/degradation ratio mass spectrometry for measuring relative dynamic protein turnover. *Anal. Chem*. 76, 86-97. doi: 10.1021/ac034841a

Carreira R.S., Lee, Y., Ghochani, M., Gustafsson, A.B., Gottlieb R.A. (2010). Cyclophilin D is required for mitochondrial removal by autophagy in cardiac cells. *Autophagy* 6, 462–472. doi: 10.4161/auto.6.4.11553

Casadesús, J., and Low, D.A. (2013). Programmed heterogeneity: epigenetic mechanisms in bacteria. *J. Biol. Chem.* 288, 13929-13935. doi: 10.1074/jbc.R113.472274

Casey, J.L., Hentze, M.W., Koeller, D.M., Caughman, S.W., Rouault, T.A., Klausner, R.D., et al. (1988). Iron-responsive elements: regulatory RNA sequences that control mRNA levels and translation. *Science*. 240, 924-928. doi: 10.1126/science.2452485

Champoux, J.J. (2001). DNA topoisomerases: structure, function, and mechanism. *Annu. Rev. Biochem*. 70, 369–413. doi:10.1146/annurev.biochem.70.1.369

Chan, C.T., Pang, Y.L., Deng, W., Babu, I.R., Dyavaiah, M., Begley, T.J., et al., (2012).  Reprogramming of tRNA modifications controls the oxidative stress response by codon-biased translation of proteins. *Nat. Commun*. 3:937. doi: 10.1038/ncomms1938

Chatterjee, S., and Grosshans, H. (2009). Active turnover modulates mature microRNA activity in Caenorhabditis elegans. *Nature* 461, 546-549. doi: 10.1038/nature08349

Chernyakov, I., Whipple, J.M., Kotelawala, L., Grayhack, E.J., and Phizicky, E.M. (2008). Degradation of several hypomodified mature tRNA species in Saccharomyces cerevisiae is mediated by Met22 and the 5'-3' exonucleases Rat1 and Xrn1. *Genes Dev.* 22, 1369-1380. doi: 10.1101/gad.1654308

Christiano, R., Henning, A., Kabatnik, S., Mejhert, N., Weng Lai, Z., Farese, R.V. Jr., et al. (2020). A Systematic Protein Turnover Map for Decoding Protein Degradation. *Cell Rep.* 33, 6. doi: 10.1016/j.celrep.2020.108378.

Christiano, R., Nagaraj, N., Fröhlich, F., and Walther, T.C. (2014). Global proteome turnover analyses of the Yeasts S. cerevisiae and S. pombe. *Cell Rep*. 9, 1959-65. doi: 10.1016/j.celrep.2014.10.065.

Coburn, G.A., and Mackie, G.A. (1999). Degradation of mRNA in Escherichia coli: an old problem with some new twists. *Prog. Nucleic Acids Res. Mol. Biol.* 62, 55–108. doi: 10.1016/s0079-6603(08)60505-x

Cole, S.E., LaRiviere, F.J., Merrikh, C.N., and Moore, M.J. (2009). A convergence of rRNA and mRNA quality control pathways revealed by mechanistic analysis of nonfunctional rRNA decay. *Mol. Cell.* 34, 440–450. doi: 10.1016/j.molcel.2009.04.017

Cooper, G.M. (2000). *The Cell: A Molecular Approach*. (4nd edition). Sunderland (MA): Sinauer Associates. Boston University Press, 463-469.

Dahms, A.S. (1974). 3-Deoxy-D-pentulosonic acid aldolase and its role in a new pathway of D-xylose degradation. *Biochem. Biophys. Res. Commun.* 60, 1433–1439. doi:10.1016/0006-291X(74)90358-1

Dame, R.T. (2005). The role of nucleoid-associated proteins in the organization and compaction of bacterial chromatin. *Mol. Microbiol.* 56, 858–870. doi:10.1111/j.1365-2958.2005.04598.x

Dawidowicz, E.A. (1987). Dynamics of membrane lipid metabolism and turnover. *Annu Rev Biochem.* 56, 43-61. doi: 10.1146/annurev.bi.56.070187.000355.

De Bont, R., and van Larebeke, N. (2004) Endogenous DNA damage in humans: a review of quantitative data. Mutagenesis 19, 169-185. doi: 10.1093/mutage/geh025

de Koning, W., and van Dam, K. (1992). A method for the determination of changes of glycolytic metabolites in yeast on a subsecond time scale using extraction at neutral pH. Anal. Biochem. 204, 118-123. doi: 10.1016/0003-2697(92)90149-2

De la Fuente, I.M. (2015). Elements of the cellular metabolic structure. *Front. Mol. Biosci.* 2:16. doi: 10.3389/fmolb.2015.00016

de Virgilio, M., Weninger, H., and Ivessa, N.E. (1998). Ubiquitination is required for the retro-translocation of a short-lived luminal endoplasmic reticulum glycoprotein to the cytosol for degradation by the proteasome. *J. Biol. Chem*. 273, 9734–9743. doi: 10.1074/jbc.273.16.9734

Delgado, F., F., Cermak, N., Hecht, V.C., Son, S., Li, Y., Knudsen, S.M., et al. (2013). Intracellular water exchange for measuring the dry mass, water mass and changes in chemical composition of living cells. *PLoS ONE* 8:e67590. doi: 10.1371/journal.pone.0067590

Deutscher, M.P. (2003). Degradation of stable RNA in bacteria. *J. Biol. Chem*. 278, 45041-45044. doi: 10.1074/jbc.R300031200

Doherty, M. K., and Beynon, R. J. (2006). Protein turnover on the scale of the proteome *Expert Rev. Proteomics* 3:1, 97-110. doi: 10.1586/14789450.3.1.97

Doherty, M. K., Hammond, D. E., Clague, M. J., Gaskell, S. J., and Beynon, R. J. (2009). Turnover of the human proteome: determination of protein intracellular stability by dynamic SILAC. *J. Proteome Res*. 8, 104–112. doi: 10.1021/pr800641v

Eagle, H., Piez, K. A., Fleischman, R., and Oyama, V. I. (1959). Protein turnover in mammalian cell cultures. *J. Biol. Chem*. 234, 592–597.

Eden, E., Geva-Zatorsky, N., Issaeva, I., Cohen, A., Dekel, E., Danon, T., et al. (2011) Proteome half-life dynamics in living human cells. Science 331, 764–768. doi: 10.1126/science.1199784

El Yacoubi, B., Bailly, M., and de Crecy-Lagard, V., (2012). Biosynthesis and function of posttranscriptional modifications of transfer RNAs. *Annu. Rev. Genet*. 46: 69–95. doi: 10.1146/annurev-genet-110711-155641

ENCODE Project Consortium; Birney, E., Stamatoyannopoulos, J.A., Dutta, A., Guigó, R., Gingeras, T.R., Margulies, E.H., et al. (2007). Identification and analysis of functional elements in 1% of the human genome by the ENCODE pilot project. *Nature* 447, 799–816. doi: 10.1038/nature05874

Englert M., and Beier H. (2005). Plant tRNA ligases are multifunctional enzymes that have diverged in sequence and substrate specificity from RNA ligases of other phylogenetic origins. *Nucleic Acids Res.* 33, 388–399. doi: 10.1093/nar/gki174

Esue, O., Rupprecht, L., Sun, S.X., and Wirtz, D. (2010). Dynamics of the bacterial intermediate filament crescentin in vitro and in vivo. *PLoS One*. 5:e8855. doi: 10.1371/journal.pone.0008855

Ezaki, J., Kominami, E., and Ueno, T. (2011). Peroxisome degradation in mammals. *IUBMB Life*. Nov;63(11):1001-8. doi: 10.1002/iub.537

Farber, S.A., Slack, B.E., and Blusztajn, J.K. (2000). Acceleration of phosphatidylcholine synthesis and breakdown by inhibitors of mitochondrial function in neuronal cells: a model of the membrane defect of Alzheimer's disease. *FASEB J*. 14, 2198-2206. doi: 10.1096/fj.99-0853

Farré, J.-C., Krick, R., Subramani, S., Thumm, M. (2009). Turnover of organelles by autophagy in yeast. *Curr. Opin. Cell Biol.* 21, 522-530. doi: 10.1016/j.ceb.2009.04.015.

Feng, P., Everly, D.N. Jr., Read, G.S. (2001). mRNA decay during herpesvirus infections: interaction between a putative viral nuclease and a cellular translation factor. *J. Virol* 75, 10272-10280. doi: 10.1128/JVI.75.21.10272-10280.2001

Freeze, H.H. (2006). Genetic defects in the human glycome. *Nat. Rev. Genet.* 7, 537–551. doi:10.1038/nrg1894.

Fujii, K., Kitabatake, M., Sakata, T., Miyata, A., and Ohno, M. (2009). A role for ubiquitin in the clearance of nonfunctional rRNAs. *Genes Dev.* 23, 963–974. doi: 10.1101/gad.1775609

Furst, W., and Sandhoff, K. (1992). Activator proteins and topology of lysosomal sphingolipid catabolism. *Biochim. Biophys. Acta* 1126, 1–16. doi: 10.1016/0005-2760(92)90210-m

Gaigg, B., Neergaard, T.B., Schneiter, R., Hansen, J.K., Faergeman, N.J., Jensen, N.A., et al. (2001). Depletion of acyl-coenzyme A-binding protein affects sphingolipid synthesis and causes vesicle accumulation and membrane defects in Saccharomyces cerevisiae. *Mol. Biol. Cell.* 12, 1147-60. doi: 10.1091/mbc.12.4.1147

García-Alai, M.M., Gallo, M., Salame, M., Wetzler, D.E., McBride, A.A., Paci, M., et al. Molecular basis for phosphorylation-dependent, PEST-mediated protein turnover. *Structure* 14, 309-319. doi: 10.1016/j.str.2005.11.012

Gegenheimer, P., Gabius, H. J., Peebles, C. L., and Abelson, J. (1983). An RNA ligase from wheat germ which participates in transfer RNA splicing in vitro. *J. Biol. Chem.* 258, 8365–8373.

Gelfand, R., and Attardi, G. (1981). Synthesis and turnover of mitochondrial ribonucleic acid in HeLa cells: the mature ribosomal and messenger ribonucleic acid species are metabolically unstable. *Mol. Cell. Biol*. 1, 497-511. doi: 10.1128/mcb.1.6.497

Geva-Zatorsky, N., Issaeva, I., Mayo, A., Cohen, A., Dekel, E., Danon, T., et al. (2012). Using bleach-chase to measure protein half-lives in living cells. *Nat. Protoc.* 7, 801-811. doi: 10.1038/nprot.2012.028

Gillard, B.K., Thurmon, L.T., and Marcus, D.M. (1993). Variable subcellular localization of glycosphingolipids. *Glycobiology* 3, 57-67. doi: 10.1093/glycob/3.1.57

Gillery, P., Georges, N., Wegrowski, J., Randoux, A., and Borel, J.P. (1995). Protein synthesis in collagen-lattice cultured fibroblasts is controlled at the ribosomal level. *FEBS Lett.* 357, 287–289. doi: 10.1016/0014-5793(94)01375-B

Glickman, M.H., and Ciechanover, A. (2002). The ubiquitin-proteasome proteolytic pathway: destruction for the sake of construction. *Physiol. Rev.* 82, 373-428. doi: 10.1152/physrev.00027.2001

Gnad, F., Ren, S., Cox, J., Olsen, J.V., Macek, B., Oroshi, M., et al. (2007). PHOSIDA (phosphorylation site database): management, structural and evolutionary investigation, and prediction of phosphosites. *Genome Biol*. 8:R250. doi: 10.1186/gb-2007-8-11-r250.

Gogol, E.B., Rhodius, V.A., Papenfort, K., Vogel, J., and Gross, C.A. (2011). Small RNAs endow a transcriptional activator with essential repressor functions for single-tier control of a global stress regulon. *Proc. Natl. Acad. Sci. U.S.A.* 108, 12875–12880. doi: 10.1073/pnas.1109379108

Gonzalez, D., Kozdon, J.B., McAdams, H.H., Shapiro, L., and Collier, J. (2014). The functions of DNA methylation by CcrM in Caulobacter crescentus: a global approach. *Nucleic Acids Res.* 42, 3720-3735. doi: 10.1093/nar/gkt1352

Göpel, Y., and Görke, B. (2012). Rewiring two-component signal transduction with small RNAs. *Curr. Opin. Microbiol.* 15, 132–139. doi: 10.1016/j.mib.2011.12.001

Göpel, Y., Papenfort, K., Reichenbach, B., Vogel, J., and Görke, B. (2013). Targeted decay of a regulatory small RNA by an adaptor protein for RNase E and counteraction by an anti-adaptor RNA. *Genes Dev.* 27, 552-564. doi: 10.1101/gad.210112.112

Gottesman, S. (2005). Micros for microbes: non-coding regulatory RNAs in bacteria. *Trends Genet*. 21, 399–404. doi: 10.1016/j.tig.2005.05.008

Gottesman, S., and Storz, G. (2011). Bacterial small RNA regulators: Versatile roles and rapidly evolving variations. *Cold Spring Harb. Perspect. Biol.* 3:a003798. doi: 10.1101/cshperspect.a003798

Gottesman, S., Maurizi, M.R., and Wickner, S. (1997). Regulatory subunits of energy-dependent proteases. *Cell* 91, 435–438. doi: 10.1016/s0092-8674(00)80428-6.

Graumann, P.L. (2009). Dynamics of bacterial cytoskeletal elements. *Cell Motil. Cytoskeleton.* 66, 909-914. doi: 10.1002/cm.20381.

Grosshans, H., and Filipowicz, W. (2008). Molecular biology: the expanding world of small RNAs. *Nature* 451, 414-416. doi: 10.1038/451414a

Gudipati, R.K., Xu, Z., Lebreton, A., Seraphin, B., Steinmetz, L.M., Jacquier, A., et al. (2012).  Extensive degradation of RNA precursors by the exosome in wild-type cells. *Mol. Cell.* 48, 409–421. doi: 10.1016/j.molcel.2012.08.018

Guillaume, R., Jacquel, A., and Auberger, P. (2019) Chaperone-Mediated Autophagy and Its Emerging Role in Hematological Malignancies. *Cells*. 8(10): 1260. doi: 10.3390/cells8101260

Gur, E., Biran, D., and Ron, E.Z. (2011). Regulated proteolysis in Gram-negative bacteria--how and when? *Nat. Rev. Microbiol.* 9, 839-848. doi: 10.1038/nrmicro2669.

Halle, J.P., Muller, S., Simm, A., and Adam, G. (1997). Copy number, epigenetic state and expression of the rRNA genes in young and senescent rat embryo fibroblasts. *Eur. J. Cell. Biol.* 74, 281– 288.

Han, X., and Gross R.W. (2005). Shotgun lipidomics: Electrospray ionization mass spectrometric analysis and quantitation of cellular lipidomes directly from crude extracts of biological samples. *Mass Spectrom. Rev.* 24, 367–412. doi: 10.1002/mas.20023

Hao, M., and Maxfield, F. R. (2000). Characterization of rapid membrane internalization and recycling. *J. Biol. Chem.* 275, 15279–15286. doi: 10.1074/jbc.275.20.15279

Harris, P.V., Welner, D., McFarland, K.C., Re, E., Poulsen, J.-C. N., et al. (2010). Stimulation of lignocellulosic biomass hydrolysis by proteins of glycoside hydrolase family 61: structure and function of a large, enigmatic family. *Biochemistry* 49, 3305–3316. doi: 10.1021/bi100009p

Hart, G.W., and Akimoto, Y. (2009) “The O-GlcNAc modification,” in: *Essentials of Glycobiology*, eds Varki, A., Cummings, RD., Esko, JD., Freeze, HH., Stanley, P., Bertozzi, C.R., Hart, G.W., and Etzler, M.E. Cold Spring Harbor (NY): Cold Spring Harbor Laboratory Press.

Henty, J.L., Bledsoe, S.W., Khurana, P., Meagher, R.B., Day, B., Blanchoin, L., et al. (2011). Arabidopsis actin depolymerizing factor4 modulates the stochastic dynamic behavior of actin filaments in the cortical array of epidermal cells. *Plant Cell*. 23, 3711-3726. doi: 10.1105/tpc.111.090670

Herrick, D., Parker, R., and Jacobson, A. (1990). Identification and comparison of stable and unstable mRNAs in Saccharomyces cerevisiae. *Mol. Cell. Biol.* 10, 2269–2284. doi: 10.1128/mcb.10.5.2269.

Hershko, A., and Ciechanover, A. (1998) The ubiquitin system. *Annu. Rev. Biochem.* 67, 425 – 479. doi: 10.1146/annurev.biochem.67.1.425

Hirsch, C., Blom, D. and Ploegh, H.L. (2003). A role for N-glycanase in the cytosolic turnover of glycoproteins. *EMBO J.,* 22, 1036–1046. doi: 10.1093/emboj/cdg107

Hopper, A.K. (2013). Transfer RNA post-transcriptional processing, turnover, and subcellular dynamics in the yeast Saccharomyces cerevisiae. *Genetics*. 194, 43-67. doi: 10.1534/genetics.112.147470

Humbard, M.A., and Maupin-Furlow, J.A. (2013). Prokaryotic proteasomes: nanocompartments of degradation. *J. Mol. Microbiol. Biotechnol.* 23, 321-34. doi: 10.1159/000351348.

Ishizuka, A., Hashimto, Y., Naka, R., Kinoshita, M., Kakehi, K., Seino, J., et al. (2008) Accumulation of free complex-type N-glycans in MKN7 and MKN45 stomach cancer cells. *Biochem. J.* 413, 227-237. doi: 10.1042/BJ20071562

Jäck, H.M., and Wabl, M. (1988). Immunoglobulin mRNA stability varies during B lymphocyte differentiation. *EMBO J.* 7, 1041-1046. doi: 10.1002/j.1460-2075.1988.tb02911.x

Jakubowski, H., and Goldman, E. (1984). Quantities of individual aminoacyl-tRNA families and their turnover in Escherichia coli. *J. Bacteriol.* 158, 769-776. doi: 10.1128/JB.158.3.769-776.1984

Kalderon, B., Mayorek, N., Berry, E., Zevit, N., and Bar-Tana, J. (2000). Fatty acid cycling in the fasting rat. *Am. J. Physiol*. *Endocrinol. Metab.* 279, E221–227. doi: 10.1152/ajpendo.2000.279.1.E221

Kamenski, P., Kolesnikova, O., Jubenot, V., Entelis, N., Krasheninnikov I. A., Martin, R.P., et al. (2007). Evidence for an adaptation mechanism of mitochondrial translation via tRNA import from the cytosol. *Mol. Cell* 26, 625–637. doi: 10.1016/j.molcel.2007.04.019

Kanerva, P.A., and Maenpaa, P.H. (1981). Codon-specific serine transfer ribonucleic acid degradation in avian liver during vitellogenin induction. *Acta. Chem. Scand.* B 35, 379–385. doi: 10.3891/acta.chem.scand.35b-0379

Karhumaa, K., Sanchez, R.G., Hahn-Hägerdal, B., and Gorwa-Grauslund, M.-F. (2007). Comparison of the xylose reductase-xylitol dehydrogenase and the xylose isomerase pathways for xylose fermentation by recombinant Saccharomyces cerevisiae. *Microb. Cell Fact.* 6:5. doi:10.1186/1475-2859-6-5

Karnahl, U., and Wasternack, C. (1992). Half-life of cytoplasmic rRNA and tRNA, of plastid rRNA and of uridine nucleotides in heterotrophically and photoorganotrophically grown cells of *Euglena gracilis* and its apoplastic mutant W3BUL. *Int. J. Biochem*. 24, 493–497. doi: 10.1016/0020-711X(92)90044-2

Kelekar, A. (2005). Autophagy. *Ann. N.Y. Acad. Sci*. 1066, 259-271. doi: 10.1196/annals.1363.015

Khaminets, A., Heinrich, T., Mari, M., Grumati, P., Huebner, A.K., Akutsu, M., et al. (2015). Regulation of endoplasmic reticulum turnover by selective autophagy. *Nature* 522, 354-358. doi: 10.1038/nature14498.

Khoury, G.A., Baliban, R.C., and Floudas, C.A. (2011). Proteome-wide post-translational modification statistics: frequency analysis and curation of the swiss-prot database. *Sci. Rep.* 1(90). doi:10.1038/srep00090.

Kim, S., Ståhlberg, J., Sandgren, M., Paton, R.S., and Beckham, G.T. (2014). Quantum mechanical calculations suggest that lytic polysaccharide monooxygenases use a copper-oxyl, oxygen-rebound mechanism. *Proc. Natl. Acad. Sci. U.S.A*. 111, 149-154. doi: 10.1073/pnas.1316609111.

Kim, W., Bennet, E.J., Huttlin, E.L., Guo, A., Li, J., Possemato, A., et al. (2011). Systematic and quantitative assessment of the ubiquitin-modified proteome. *Mol. Cell*. 44, 325–340. doi: 10.1016/j.molcel.2011.08.025

Kim, Y.J., and Wilson, D.M. (2012). Overview of base excision repair biochemistry. *Curr. Mol. Pharmacol.* 5, 3-13. doi: 10.2174/1874467211205010003

Kolter, T. (2011). A view on sphingolipids and disease. *Chem. Phys. Lipids.* 164, 590-606. doi: 10.1016/j.chemphyslip.2011.04.013.

Kolter, T., and Sandhoff, K. (2005). Principles of lysosomal membrane digestion: stimulation of sphingolipid degradation by sphingolipid activator proteins and anionic lysosomal lipids. *Annu. Rev. Cell Dev. Biol.* 21, 81-103. doi: 10.1146/annurev.cellbio.21.122303.120013.

Kotelawala, L., Grayhack, E.J., and Phizicky, E.M. (2008). Identification of yeast tRNA Um44 2′-O-methyltransferase (Trm44) and demonstration of a Trm44 role in sustaining levels of specific tRNASer species. *RNA* 14, 158–169. doi: 10.1261/rna.811008

Kraft, C., Deplazes, A., Sohrmann, M., and Peter, M. (2008). Mature ribosomes are selectively degraded upon starvation by an autophagy pathway requiring the Ubp3p/Bre5p ubiquitin protease. *Nat. Cell Biol.* 10, 602-610. doi: 10.1038/ncb1723.

Kreisel, W., Volk, B.A., Büchsel, R., Reutter, W. (1980). Different half-lives of the carbohydrate and protein moieties of a 110,000-dalton glycoprotein isolated from plasma membranes of rat liver*. Proc. Natl. Acad. Sci. U.S.A.* 77, 1828-1831. doi: 10.1073/pnas.77.4.1828

Krol, J., Busskamp, V., Markiewicz, I., Stadler, M.B., Ribi, S., Richter, J., et al. (2010) Characterizing light-regulated retinal microRNAs reveals rapid turnover as a common property of neuronal microRNAs. *Cell* 141, 618–631. doi: 10.1016/j.cell.2010.03.039

Krol, J., Loedige, I., and Filipowicz, W. (2010). The widespread regulation of microRNA biogenesis, function and decay. *Nat. Rev. Genet*. 11, 597-610. doi: 10.1038/nrg2843

Kruger, N.J., and von Schaewen, A. (2003). The oxidative pentose phosphate pathway: structure and organisation. *Curr. Opin. Plant Biol.* 6, 236–246. doi:10.1016/S1369-5266(03)00039-6.

Kung, L.A., Tao, S.C., Qian, J., Smith, M.G., Snyder, M., and Zhu, H. (2009). Global analysis of the glycoproteome in Saccharomyces cerevisiae reveals new roles for protein glycosylation in eukaryotes. *Mol. Syst. Biol.* 5:308. doi: 10.1038/msb.2009.64

Lecker, H.S., and Mitch E.W. (2011). Proteolysis by the ubiquitin-proteasome system and kidney disease. *J. Am. Soc. Nephrol.* 22(5):821-4. doi: 10.1681/ASN.2010090958

Lahtvee, P.J., Seiman, A., Arike, L., Adamberg, K., and Vilu, R. (2014). Protein turnover forms one of the highest maintenance costs in Lactococcus lactis. *Microbiology* 160(Pt 7),1501-1512. doi: 10.1099/mic.0.078089-0.

Larance, M., Ahmad, Y., Kirkwood, K. J., Ly, T., and Lamond, A. I. (2013). Global subcellular characterization of protein degradation using quantitative proteomics. Mol. *Cell. Proteom*. 12, 638–650. doi: 10.1074/mcp.M112.024547

LaRiviere, F.J., Wolfson, A.D., and Uhlenbeck, O.C. (2001). Uniform binding of aminoacyl-tRNAs to elongation factor Tu by thermodynamic compensation. *Science* 294, 165–168. doi: 10.1126/science.1064242

Lecker, S.H., Goldberg, A.L., and Mitch, W.E. (2006). Protein degradation by the ubiquitin-proteasome pathway in normal and disease states. *J. Am. Soc. Nephrol.* 17, 1807-1819. doi: 10.1681/ASN.2006010083

Lee, S.M., Jellison, T., and Alper, H.S. (2012). Directed evolution of xylose isomerase for improved xylose catabolism and fermentation in the yeast Saccharomyces cerevisiae. *Appl. Environ. Microbiol.* 78, 5708-5716. doi: 10.1128/AEM.01419-12.

Levasseur, A., Drula, E., Lombard, V., Coutinho, P.M., and Henrissat, B. (2013) Expansion of the enzymatic repertoire of the CAZy database to integrate auxiliary redox enzymes*. Biotechnol. Biofuels* 6:41. doi: 10.1186/1754-6834-6-41

Li, X., Benning, C., and Kuo, M.H. (2012). Rapid triacylglycerol turnover in Chlamydomonas reinhardtii requires a lipase with broad substrate specificity. *Eukaryot. Cell* 11, 1451-1462. doi: 10.1128/EC.00268-12

Li, Z., Wang, Y., Yao, Q., Justice, N.B., Ahn, T.H., Xu, D., et al. (2014). Diverse and divergent protein post-translational modifications in two growth stages of a natural microbial community. *Nat. Commun.* 5, 4405. doi: 10.1038/ncomms5405.

Lindahl, T. (1993). Instability and decay of the primary structure of DNA. *Nature,* 362, 709-715. doi: 10.1038/362709a0

Liu, K., and Czaja, M.J. (2013). Regulation of lipid stores and metabolism by lipophagy. *Cell Death Differ.* 20, 3-11. doi: 10.1038/cdd.2012.63

Lonhienne, T.G., Sagulenko, E., Webb, R.I., Lee, K.C., Franke, J., Devos, D.P., et al. (2010). Endocytosis-like protein uptake in the bacterium Gemmata obscuriglobus. *Proc. Natl. Acad. Sci. U.S.A*. Jul 20;107(29):12883-12888. doi: 10.1073/pnas.1001085107

Loriaux, P.M., and Hoffmann, A. (2013). A Protein Turnover Signaling Motif Controls the Stimulus-Sensitivity of Stress Response Pathways. *PLoS Comput. Biol.* 9:e1002932. doi: 10.1371/journal.pcbi.1002932

Lupas, A., Flanagan, J.M., Tamura, T., and Baumeister, W. (1997) Self-compartmentalizing proteases. *Trends Biochem. Sci*. 22, 399–404. doi: 10.1016/s0968-0004(97)01117-1

Mackie, G.A. (2012). RNase E: at the interface of bacterial RNA processing and decay. Nat Rev Microbiol. 11, 45-57. doi: 10.1038/nrmicro2930

Malhotra, R. (2012). Membrane glycolipids: functional heterogeneity: a review. *Biochem. Anal. Biochem.* 1:108. doi:10.4172/2161-1009.1000108

Mank, N.N., Berghoff, B.A., Hermanns, Y.N., and Klug, G. (2012). Regulation of bacterial photosynthesis genes by the small noncoding RNA PcrZ. *Proc. Natl. Acad. Sci.* *U.S.A.* 109, 16306-16311. doi: 10.1073/pnas.1207067109

Marguerat, S., Schmidt, A., Codlin, S., Chen, W., Aebersold, R., and Bähler, J. (2012). Quantitative analysis of fission yeast transcriptomes and proteomes in proliferating and quiescent cells. *Cell* 151, 671-683. doi: 10.1016/j.cell.2012.09.019

Marteijn, J.A., Lans, H., Vermeulen, W., and Hoeijmakers, J.H. (2014). Understanding nucleotide excision repair and its roles in cancer and ageing. *Nat. Rev. Mol. Cell Biol.* 15, 465-481. doi: 10.1038/nrm3822

Mathieson, T., Franken, H., Kosinski, J., Kurzawa, N., Zinn, N., Sweetman, G., et al. Systematic analysis of protein turnover in primary cells. *Nat. Commun*. 9, 689. doi: 10.1038/s41467-018-03106-1.

Maupin-Furlow, J. (2011). Proteasomes and protein conjugation across domains of life. *Nat. Rev. Microbiol.* 19;10, 100-111. doi: 10.1038/nrmicro2696.

Maxfield, F.R., and McGraw, T.E. (2004). Endocytic recycling. *Nat. Rev. Mol. Cell Biol.* 5, 121-132. doi: 10.1038/nrm1315

Mayor, S., Presley, J.F. and Maxfield, F.R. (1993). Sorting of membrane components from endosomes and subsequent recycling to the cell surface occurs by a bulk flow process. J. Cell Biol. 121, 1257–1269. doi: 10.1083/jcb.121.6.1257

Mayor, S., Sabharanjak, S. and Maxfield, F.R. (1998). Cholesterol-dependent retention of GPI-anchored proteins in endosomes. *EMBO J.* 17, 4626–4638. doi: 10.1093/emboj/17.16.4626

Meyer, S., Temme, C., and Wahle, E. (2004). Messenger RNA turnover in eukaryotes: pathways and enzymes. *Crit. Rev. Biochem. Mol. Biol*. 39, 197-216.

Michalik, S., Liebeke, M., Zühlke, D., Lalk, M., Bernhardt, J., Gerth, U., et al. (2009) Proteolysis during long-term glucose starvation in Staphylococcus aureus COL. *Proteomics*. 9, 4468-4477. doi: 10.1002/pmic.200900168.

Mijaljica, D., and Devenish, R.J. (2013). Nucleophagy at a glance. *J. Cell. Sci.* 126(Pt 19), 4325-4330. doi: 10.1242/jcs.133090

Mijaljica, D., Prescott, M., and Devenish, R.J. (2010). The intricacy of nuclear membrane dynamics during nucleophagy. *Nucleus*. 1, 213-223. doi: 10.4161/nucl.1.3.11738

Miller, C., Schwalb, B., Maier, K., Schulz, D., Dümcke, S., Zacher, B., et al. (2011). Dynamic transcriptome analysis measures rates of mRNA synthesis and decay in yeast. *Mol. Syst. Biol.* 7:458. doi: 10.1038/msb.2010.112

Mitchell, P.J., and Tjian, R. (1989). Transcriptional regulation in mammalian cells by sequence-specific DNA binding proteins. *Science* 245:371–378. doi:10.1126/science.2667136

Miwa, S., Lawless, C., and von Zglinicki, T. (2008). Mitochondrial turnover in liver is fast in vivo and is accelerated by dietary restriction: application of a simple dynamic model. *Aging Cell.* 7(6):920-3. doi: 10.1111/j.1474-9726.2008.00426.x

Mizushima, N., Ohsumi, Y., and Yoshimori, T. (2002). Autophagosome formation in mammalian cells. *Cell Struct. Funct*. 27, 421–429. doi: 10.1247/csf.27.421

Mogelsvang, S., Gomez-Ospina, N., Soderholm, J., Glick, B.S., and Staehelin, L.A. (2003). Tomographic evidence for continuous turnover of Golgi cisternae in Pichia pastoris. *Mol. Biol. Cell.* 14, 2277-2291. doi: 10.1091/mbc.e02-10-0697

Morell, P., and Ousley, A.H. (1994). Metabolic turnover of myelin glycerophospholipids. *Neurochem. Res.* 19, 967-974. doi: 10.1007/BF00968706

Morris, T.D., Weber, L.A., Hickey, E., Stein, G.S., and Stein, J.L. (1991). Changes in the stability of a human H3 histone mRNA during the HeLa cell cycle. *Mol. Cell Biol.* 11, 544-553. doi: 10.1128/mcb.11.1.544

Mudd, J. B. (1980). “Phospholipid biosynthesis,” in *Biochemistry of Plants: A Comprehensive Treatise*, ed. Stumpf, P. K. (New York: Academic Press), Vol. 4, 250-280.

Myers, L.C., and Kornberg, R.D. (2000). Mediator of transcriptional regulation. *Annu. Rev. Biochem.* 69, 729–749. doi:10.1146/annurev.biochem.69.1.729

Narlikar, G.J., Fan, H.Y., and Kingston, R.E. (2002). Cooperation between complexes that regulate chromatin structure and transcription. *Cell* 108, 475–487. doi:10.1016/S0092-8674(02)00654-2

Nelson, D.L., and Cox, M.M. (2008). *Lehninger Principles of Biochemistry*. New York: W.H. Freeman.

Nercessian, D., and Conde, R.D. (2006). Control of ribosome turnover during growth of the haloalkaliphilic archaeon *Natronococcus occultus*. *Res Microbiol.* 157, 625-628. doi: 10.1016/j.resmic.2006.01.001

Nwagwu, M., and Nana, M. (1980). Ribonucleic acid synthesis in embryonic chick muscle, rates of synthesis and half-lives of transfer and ribosomal RNA species. *J. Embryol. Exp. Morphol*. 56, 253–267.

O'Dea, E.L., Barken, D., Peralta, R.Q., Tran, K.T., Werner, S.L., Keans, J.D., et al. (2007). A homeostatic model of IkB metabolism to control constitutive NF-kB activity. *Mol. Syst. Biol.* 3:111. doi: 10.1038/msb4100148

Ohashi, S., Iwai, K., Mega, T., and Hase, S. (1999) Quantitation and isomeric structure analysis of free oligosaccharides present in the cytosol fraction of mouse liver: detection of a free disialobiantennary oligosaccharide and glucosylated oligomannosides. *J. Biochem.* 126, 852-858. doi: 10.1093/oxfordjournals.jbchem.a022526

Okuda, S., and Eiraku M. (2017). Role of molecular turnover in dynamic deformation of a three-dimensional cellular membrane. *Biomech. Model. Mechanobiol*. 16, 1805–1818. doi: 10.1007/s10237-017-0920-8

Omura, T., Siekevitz, P., and Palade, G.E. (1967). Turnover of constituents of the endoplasmic reticulum membranes of rat hepatocytes. *J. Biol. Chem*. 242, 2389-2396.

Park, J. T. (2001). Identification of a dedicated recycling pathway for anhydro-N-acetylmuramic acid and N-acetylglucosamine derived from Escherichia coli cell wall murein. *J. Bacteriol.* 183, 3842–3847. doi: 10.1128/JB.183.13.3842-3847.2001

Park, J.T., and Uehara, T. (2008). How bacteria consume their own exoskeletons (turnover and recycling of cell wall peptidoglycan). *Microbiol. Mol. Biol. Rev*. 72, 211-227. doi: 10.1128/MMBR.00027-07

Parsons, J.B., and Rock, C.O. (2013). Bacterial lipids: metabolism and membrane homeostasis. *Prog. Lipid Res.* 52, 249-76. doi: 10.1016/j.plipres.2013.02.002

Pedersen, J.M., Fredsoe, J., Roedgaard, M., Andreasen, L., Mundbjerg, K., Kruhøffer, M., et al. (2012) DNA Topoisomerases Maintain Promoters in a State Competent for Transcriptional Activation in Saccharomyces cerevisiae. *PLoS Genet.* 8:e1003128. doi:10.1371/journal.pgen.1003128

Pérez-Ortín, J.E., Alepuz, P., Chávez, S., and Choder, M. (2013). Eukaryotic mRNA decay: Methodologies, pathways, and links to other stages of gene expression. *J. Mol. Biol.*, 425, 3750–3775. doi: 10.1016/j.jmb.2013.02.029

Phillips, R., Ursell, T., Wiggins, P., and Sens, P. (2009). Emerging roles for lipids in shaping membrane-protein function. *Nature* 459, 379-385. DOI: 10.1038/nature08147

Phizicky, E. M., and Hopper, A. K. (2010). tRNA biology charges to the front. *Genes Dev.* 24, 1832–1860. doi: 10.1101/gad.1956510

Popow, J., Englert, M., Weitzer, S., Schleiffer, A., Mierzwa, B., Mechtler, K., et al. (2011). HSPC117 is the essential subunit of a human tRNA splicing ligase complex. *Science* 331, 760–764. doi: 10.1126/science.1197847

Popow, J., Schleiffer, A., and Martinez J. (2012). Diversity and roles of (t)RNA ligases. *Cell. Mol. Life Sci.* 69, 2657–2670. doi: 10.1007/s00018-012-0944-2

Princiotta, M.F., Finzi, D., Qian, S.-B., Gibbs, J., Schuchmann, S., Buttgereit, F., et al. (2003) Quantitating protein synthesis, degradation, and endogenous antigen processing. *Immunity* 18, 343–354. doi: 10.1016/s1074-7613(03)00051-7

Prinetti, A., Loberto, N., Chigorno, V., and Sonnino, S. (2009) Glycosphingolipid behaviour in complex membranes. *Biochim. Biophys. Acta* 1788, 184–193. 10.1016/j.bbamem.2008.09.001

Qingbo, Li (2010). Advances in protein turnover analysis at the global level and biological insights. *Mass Spectrom. Rev.* 29, 717–736. doi:10.1002/mas.20261

Qiu, R., Sakato, M., Sacho, E.J., Wilkins, H., Zhang, X., Modrich, P., et al. (2015). MutL traps MutS at a DNA mismatch. *Proc. Natl. Acad. Sci.* *U.S.A.* 112, 10914-10919. doi: 10.1073/pnas.1505655112

Rajasethupathy, P., Fiumara, F., Sheridan, R., Betel, D., Puthanveettil, S.V., Russo, J.J., et al. (2009). Characterization of small RNAs in aplysia reveals a role for miR-124 in constraining synaptic plasticity through CREB. Neuron, 63, 803–817. doi: 10.1016/j.neuron.2009.05.029

Ramachandran, V., Chen, X. (2008). Degradation of microRNAs by a family of exoribonucleases in Arabidopsis. *Science*. 321, 1490–1492. DOI: 10.1126/science.1163728

Ramagopal, S. (1984). Metabolic changes in ribosomes of *Escherichia coli* during prolonged culture in different media. *Eur. J. Biochem*. 140, 353-361. doi: 10.1111/j.1432-1033.1984.tb08108.x

Rastogi, S.C. (2010). *Biochemistry*, 3rd edn. New Delhi: Tata McGraw-Hill Education.

Reed, S. (2003) Ratchets and clocks: the cell cycle, ubiquitylation and protein turnover. *Nat Rev Mol Cell Biol* 4, 855–864. doi.org/10.1038/nrm1246

Reihsaus, E., Kohler, M., Kraiss, S., Oren, M., and Montenarh, M. (1990). Regulation of the level of the oncoprotein p53 in non-transformed and transformed cells. *Oncogene* 5, 137–145.

Reimold, C., Defeu Soufo, H.J., Dempwolff, F., and Graumann, P.L. (2013). Motion of variable-length MreB filaments at the bacterial cell membrane influences cell morphology. *Mol. Biol. Cell.* 24, 2340-2349. doi: 10.1091/mbc.E12-10-0728

Reith, J., and Mayer, C. (2011). Peptidoglycan turnover and recycling in Gram-positive bacteria. *Appl. Microbiol. Biotechnol*. 92, 1-11. doi: 10.1007/s00253-011-3486-x

Richards, G.R., and Vanderpool, C.K. (2011). Molecular call and response: The physiology of bacterial small RNAs. *Biochim. Biophys. Acta* 1809, 525–531. doi: 10.1016/j.bbagrm.2011.07.013

Rimsky, S., and Travers, A. (2011). Pervasive regulation of nucleoid structure and function by nucleoid-associated proteins. *Curr. Opin. Microbiol*. 14, 136-141. doi: 10.1016/j.mib.2011.01.003

Robert, G., Jacquel, A., and Auberger, P. (2019). Chaperone-mediated autophagy and its emerging role in hematological malignancies. *Cells* 8:1260. doi: 10.3390/cells8101260

Rock, CO. (1984). Turnover of fatty acids in the 1-position of phosphatidylethanolamine in *Escherichia coli*. *J. Biol. Chem.* 259, 6188-6194.

Romano, A.H., and Conway, T. (1996). Evolution of carbohydrate metabolic pathways. *Res. Microbiol.* 147, 448–455. doi: 10.1016/0923-2508(96)83998-2

Rosenberger, T.A., Oki, J., Purdon, A.D., Rapoport, S.I., Murphy, E.J. (2002). Rapid synthesis and turnover of brain microsomal ether phospholipids in the adult rat. *J. Lipid Res.* 43, 59-68.

Rosenblatt, J., Agnew, B.J., Abe, H., Bamburg, J.R., and Mitchison, T. J. (1997). Xenopus actin depolymerizing factor/cofilin (XAC) is responsible for the turnover of actin filaments in Listeria monocytogenes tails*. J. Cell* Biol. 136, 1323–1332. 10.1083/jcb.136.6.1323

Ross, J. (1996). Control of messenger RNA stability in higher eukaryotes. *Trends Genet.* 12, 171-175. doi: 10.1016/0168-9525(96)10016-0

Rüegger S, and Großhans H. (2012). MicroRNA turnover: when, how, and why. *Trends Biochem. Sci.* 37, 436-446. doi: 10.1016/j.tibs.2012.07.002

Russell, D.H., and Snyder, S.H. (1969). Amine synthesis in regenerating rat liver: extremely rapid turnover of ornithine decarboxylase. *Mol. Pharmacol*. 5, 253–262.

Salmon, E.D., Leslie, R.J., Saxton, W.M., Karow, M.L., and McIntosh, J.R. (1984). Spindle microtubule dynamics in sea urchin embryos: analysis using a fluorescein-labeled tubulin and measurements of fluorescence redistribution after laser photobleaching. *J. Cell Biol.* 99, 2165-2174. doi: 10.1083/jcb.99.6.2165

Sanders, R.J., Ofman, R., Dacremont, G., Wanders, R.J., and Kemp, S. (2008). Characterization of the human omega-oxidation pathway for omega-hydroxy-very-long-chain fatty acids. *FASEB J*. 22, 2064-2071. doi: 10.1096/fj.07-099150

Sandhoff, K., and Kolter, T. (1996). Topology of glycosphingolipid degradation. *Trends Cell Biol*. 6, 98–103. doi: 10.1016/0962-8924(96)80999-8

Sandhoff, K., and Kolter, T. (2003). Biosynthesis and degradation of mammalian glycosphingolipids. *Philos. Trans. R. Soc. Lond. B Biol. Sci.* 358, 847-861. doi: 10.1098/rstb.2003.1265

Saxton, W.M., Stemple, D.L., Leslie, R.J., Salmon, E.D., Zavortink, M., and McIntosh, J.R. (1984). Tubulin dynamics in cultured mammalian cells. *J. Cell Biol.* 99, 2175-2186. doi: 10.1083/jcb.99.6.2175

Schäferkordt, J., and Wagner, R. (2001). Effects of base change mutations within an Escherichia coli ribosomal RNA leader region on rRNA maturation and ribosome formation. *Nucleic Acids Res*. 29, 3394-3403. doi: 10.1093/nar/29.16.3394

Schoenberg, D.R., and Maquat, L.E. (2012). Regulation of cytoplasmic mRNA decay. *Nat. Rev. Genet.* 13, 246–259. doi: 10.1038/nrg3160

Schubert, U., Anton, L.C., Gibbs, J., Norbury, C.C., Yewdell, J.W., and Bennink, J.R. (2000). Rapid degradation of a large fraction of newly synthesized proteins by proteasomes. *Nature* 404, 770–774. doi: 10.1038/35008096

Schuck, S., Gallagher, C.M., and Walter, P. (2014). ER-phagy mediates selective degradation of endoplasmic reticulum independently of the core autophagy machinery. *J. Cell Sci.* 127(Pt 18), 4078-4088. doi: 10.1242/jcs.154716

Schwanhäusser, B., Busse, D., Li, N., Dittmar, G., Schuchhardt, J., Wolf, J., et al. (2011). Global quantification of mammalian gene expression control. *Nature*. 473, 337-342. doi: 10.1038/nature10098

Schwudke, D., Schuhmann, K., Herzog, R., Bornstein, S.R., and Shevchenko, A. (2011) Shotgun lipidomics on high resolution mass spectrometers. *Cold. Spring. Harb. Perspect. Biol.* 3:a004614. doi: 10.1101/cshperspect.a004614

Seibold, G., Dempf, S., Schreiner, J., and Eikmanns, B.J. (2007). Glycogen formation in Corynebacterium glutamicum and role of ADP-glucose pyrophosphorylase. *Microbiology*. 153(Pt 4), 1275-1285. doi: 10.1099/mic.0.2006/003368-0

Seino, J., Wang, L., Harada, Y., Huang, C., Ishii, K., Mizushima, N., et al. (2013). Basal autophagy is required for the efficient catabolism of sialyloligosaccharides. *J. Biol. Chem.* 288, 26898-26907. doi: 10.1074/jbc.M113.464503

Sethi, P., and Lukiw, W.J. (2009). Micro-RNA abundance and stability in human brain: specific alterations in Alzheimer’s disease temporal lobe neocortex. *Neurosci. Lett*. 459, 100–104. doi: 10.1016/j.neulet.2009.04.052

Sharova, L.V., Sharov, A.A., Nedorezov, T., Piao, Y., Shaik, N., and Ko, M.S. (2009). Database for mRNA half-life of 19977 genes obtained by DNA microarray analysis of pluripotent and differentiating mouse embryonic stem cells. *DNA Res.* 16, 45-58. doi: 10.1093/dnares/dsn030.

Sheff, D.R., Daro, E.A., Hull, M., and Mellman, I. (1999). The receptor recycling pathway contains two distinct populations of early endosomes with different sorting functions. *J. Cell Biol.* 145, 123–139. doi: 10.1083/jcb.145.1.123

Shen, Z.J., and Malter, J.S. (2015). Regulation of AU-rich element RNA binding proteins by phosphorylation and the prolyl isomerase pin1. *Biomolecules* 5, 412-434. doi: 10.3390/biom5020412

Slomovic, S., Laufer, D., Geiger, D., and Schuster, G. (2006). Polyadenylation of ribosomal RNA in human cells. *Nucleic Acids Res.* 34, 2966-2975. doi: 10.1093/nar/gkl357

Smith, Z.D., and Meissner, A. (2013). DNA methylation: roles in mammalian development. *Nat. Rev. Genet*. 14, 204-220. doi: 10.1038/nrg3354

Sommer, T., and Wolf, D.H. (1997). Endoplasmic reticulum degradation: reverse protein flow of no return*. FASEB J*. 11, 1227-1233. doi: 10.1096/fasebj.11.14.9409541

Sonnino, S., Prinetti, A., Nakayama, H., Yangida, M., Ogawa, H., and Iwabuchi, K. (2009). Role of very long fatty acid-containing glycosphingolipids in membrane organization and cell signaling: the model of lactosylceramide in neutrophils. *Glycoconj. J.* 26, 615–621. doi: 10.1007/s10719-008-9215-8

Spagnolo, L., Rivera-Calzada, A., Pearl, L.H., and Llorca, O. (2006). Three-dimensional structure of the human DNA-PKcs/Ku70/Ku80 complex assembled on DNA and its implications for DNA DSB repair. *Mol. Cell.* 22, 511-519. doi: 10.1016/j.molcel.2006.04.013

Spiegelman, B.M., and Heinrich, R. (2004). Biological control through regulated transcriptional coactivators. *Cell* 119, 157–167. doi:10.1016/j.cell.2004.09.037

Spiro, R.G. (2002). Protein glycosylation: nature, distribution, enzymatic formation, and disease implications of glycopeptide bonds. *Glycobiology* 12, 43R-56R.

Storz, G., Altuvia, S., and Wassarman, K.M. (2005). An abundance of RNA regulators. *Annu. Rev. Biochem*. 74, 199–217. doi: 10.1146/annurev.biochem.74.082803.133136

Storz, G., Vogel, J., and Wassarman, K.M. (2011). Regulation by small RNAs in bacteria: Expanding frontiers. *Mol. Cell* 43, 880–891. doi: 10.1016/j.molcel.2011.08.022

Stotland A., and Gottlieb, R.A. (2015). Mitochondrial quality control: Easy come, easy go *Biochim. Biophys. Acta*.1853 (10 Pt B), 2802-2811. doi: 10.1016/j.bbamcr.2014.12.041

Suvorov, M., Lee, M., Hesek, D., Boggess, B., and Shahriar Mobashery (2008). Lytic Transglycosylase MltB of Escherichia coli and Its Role in Recycling of Peptidoglycan Strands of Bacterial Cell Wall. *J. Am. Chem. Soc.* 130, 11878–11879. doi: 10.1021/ja805482b

Suzuki, T. (2007) Cytoplasmic peptide: N-glycanase and catabolic pathway for free Nglycans in the cytosol. *Semin. Cell. Dev. Biol.* 18, 762-769. doi: 10.1016/j.semcdb.2007.09.010

Suzuki, T. (2009) Introduction to "Glycometabolome". *TIGG* 21, 219-227. doi: 10.4052/tigg.21.219

Suzuki, T., and Lennarz, W.J. (2003). Hypothesis: a glycoprotein-degradation complex formed by protein–protein interaction involves cytoplasmic peptide: N-glycanase. *Biochem. Biophys. Res. Commun*. 302, 1–5. doi: 10.1016/s0006-291x(03)00052-4

Suzuki, T., Kitajima, K., Inoue, S., and Inoue, Y. (1994) Occurrence and biological roles of “proximal glycanases” in animal cells. *Glycobiology* 4, 777–789. doi: 10.1093/glycob/4.6.777

Takeda, T., Yun, C-S., Shintani, M., Yamane, H., and Nojiri, H. (2011). Distribution of genes encoding nucleoid-associated protein homologs in plasmids. *Int. J. Evol. Biol.* 2011:685015. doi: 10.4061/2011/685015.

Tan, M., Luo, H., Lee, S., Jin, F., Yang, J.S., Montellier, E. et al. (2011). Identification of 67 histone marks and histone lysine crotonylation as a new type of histone modification. *Cell* 146, 1016-1028. doi:10.1016/j.cell.2011.08.00

Tani, H., Mizutani, R., Salam, K.A., Tano, K., Ijiri, K., Wakamatsu, A., et al. (2012). Genome-wide determination of RNA stability reveals hundreds of short-lived noncoding transcripts in mammals. *Genome Res.* 22, 947-956. doi: 10.1101/gr.130559.111

Tauber, R., Park, C.S., and Reutter, W. (1983). Intramolecular heterogeneity of degradation in plasma membrane glycoproteins: evidence for a general characteristic. *Proc. Natl. Acad. Sci. U.S.A.* 80, 4026-4029. doi: 10.1073/pnas.80.13.4026

Tettamanti, G. (2004). Ganglioside/glycosphingolipid turnover: New concepts. *Glycoconj. J.* 20, 301-317. doi: 10.1023/B:GLYC.0000033627.02765.cc

Theriot, J. A., and Mitchison, T. J. (1991). Actin microfilament dynamics in locomoting cells. *Nature* 352, 126–131. doi: 10.1038/352126a0

Thilom L. (1985). Quantification of endocytosis-derived membrane traffic. *Biochim Biophys. Acta.* 822, 243–266.doi: 10.1016/0304-4157(85)90010-3

Thomson, M., and Gunawardena, J. (2009). Unlimited multistability in multisite phosphorylation systems. *Nature*. 460, 274-277. doi: 10.1038/nature08102.

Thore, S., Wuttke, A., and Tengholm, A. (2007). Rapid turnover of phosphatidylinositol-4,5-bisphosphate in insulin-secreting cells mediated by Ca2+ and the ATP-to-ADP ratio. *Diabetes* 56, 818-26. doi: 10.2337/db06-0843

Trötschel, C., Albaum, S.P., and Poetsch, A. (2013). Proteome turnover in bacteria: current status for *Corynebacterium glutamicum* and related bacteria. *Microb. Biotechnol*. 6, 708-719. doi: 10.1111/1751-7915.12035

Trötschel, C., Albaum, S.P., Wolff, D., Schröder, S., Goesmann, A., Nattkemper, T.W., et al. (2012). Protein turnover quantification in a multilabeling approach: from data calculation to evaluation. *Mol. Cell Proteomics* 11, 512-526. doi: 10.1074/mcp.M111.014134

Utsugi, A., Kanda, A., and Hara, S. (2009). Lipase specificity in the transacylation of triacylglycerin. *J. Oleo Sci.* 58, 123–132. doi: 10.5650/jos.58.123

Vaaje-Kolstad, G., Westereng, B., Horn, S.J., Liu, Z., Zhai, H., Sørlie, M. et al. (2010). An oxidative enzyme boosting the enzymatic conversion of recalcitrant polysaccharides. *Science* 330, 219–222. doi: 10.1126/science.1192231

Van der Goot, F. G., and Gruenberg, J. (2002). Oiling the wheels of the endocytic pathway. *Trends Cell Biol.* 12, 296–299. doi: 10.1016/S0962-8924(02)02307-3

van Echten-Deckert, G., Klein, A., Linke, T., Heinemann, T., Weisgerber, J., and Sandhoff, K. (1997). Turnover of endogenous ceramide in cultured normal and Farber fibroblasts. *J. Lipid Res.* 38, 2569-2579.

van Meer, G., Voelker, D.R., and Feigenson, G.W. (2008). Membrane lipids: where they are and how they behave. *Nat. Rev. Mol. Cell. Biol.* 9, 112-124. doi: 10.1038/nrm2330.

Vikstrom, K.L., Lim, S.S., Goldman, R.D., and Borisy, G.G. (1992). Steady state dynamics of intermediate filament networks. *J. Cell Biol.* 118, 121-129. doi: 10.1083/jcb.118.1.121

Vollmer, W., Joris, B., Charlier, P., and Foster, S. (2008). Bacterial peptidoglycan (murein) hydrolases. *FEMS Microbiol. Rev*. 32, 259-286. doi: 10.1111/j.1574-6976.2007.00099.x

von der Haar, T.A. (2008) quantitative estimation of the global translational activity in logarithmically growing yeast cells. *BMC Syst. Biol.* 2:87. doi: 10.1186/1752-0509-2-87

Waldron, C., and Lacroute, F. (1975). Effect of growth rate on the amounts of ribosomal and transfer ribonucleic acids in yeast. J. Bacteriol. 122, 855–865. doi: 10.1128/JB.122.3.855-865.1975

Wang, Y., Liu, C.L., Storey, J.D., Tibshirani, R.J., Herschlag, D., and Brown, P.O. (2002). Precision and functional specificity in mRNA decay. *Proc. Natl. Acad. Sci. U.S.A*. 99, 5860-5865. doi: 10.1073/pnas.092538799

Wang, Z.X., Deng, R.P., Jiang, H.W., Guo, S.J., Le, H.Y., Zhao, X.D., et al. Global identification of prokaryotic glycoproteins based on an Escherichia coli proteome microarray. *PLoS One* 7:e49080. doi: 10.1371/journal.pone.0049080

Weibel, E.R., Stäubli, W., Gnägi, H.R., and Hess, F.A. (1969). Correlated morphometric and biochemical studies of the liver cell. *J. Cell Biol.* 42, 68–91. doi: 10.1083/jcb.42.1.68

Weimberg, R. (1961). Pentose oxidation by Pseudomonas fragi. *J. Biol. Chem*. 236, 629–636.

Wenk, M.R. (2010). Lipidomics: new tools and applications. *Cell* 10;143, 888-895. doi: 10.1016/j.cell.2010.11.033

Whipple, J.M., Lane, E.A., Chernyakov, I., D'Silva, S., and Phizicky, E.M. (2011). The yeast rapid tRNA decay pathway primarily monitors the structural integrity of the acceptor and T-stems of mature tRNA. *Genes Dev*. 25:1173-1184. doi: 10.1101/gad.2050711

White, U., and Ravussin, E. (2019). Dynamics of adipose tissue turnover in human metabolic health and disease. *Diabetologia* 62, 17-23 doi: 10.1007/s00125-018-4732-x

Wilbrink, M.H., Petrusma, M., Dijkhuizen, L., and van der Geize, R. (2011). FadD19 of Rhodococcus rhodochrous DSM43269, a steroid-coenzyme A ligase essential for degradation of C-24 branched sterol side chains. *Appl. Environ. Microbiol*. 77, 4455-4464. doi: 10.1128/AEM.00380-11

Winchester, B. (2005). Lysosomal metabolism of glycoproteins. *Glycobiology* 15, 1R-15R. doi: 10.1093/glycob/cwi041

Wolfenden, R., and Snider, M.J. (2001). The depth of chemical time and the power of enzymes as catalysts. *Acc. Chem. Res.* 34, 938-945. doi: 10.1021/ar000058i

Xie, Y., Li, J., Kang, R., and Tang, D. (2020). Interplay Between Lipid Metabolism and Autophagy. *Front. Cell. Dev. Biol*. 8, 431. doi: 10.3389/fcell.2020.00431.

Xu, L., Glass, C.K., and Rosenfeld, M.G. (1999). Coactivator and corepressor complexes in nuclear receptor function. *Curr. Opin. Genet. Dev.* 9, 140-147. doi:10.1016/S0959-437X(99)80021-5

Yang, E., van Nimwegen, E., Zavolan, M., Rajewsky, N., Schroeder, M., Magnasco, M., et al. (2003). Decay rates of human mRNAs: correlation with functional characteristics and sequence attributes. *Genome Res.* 13, 1863-1872. doi: 10.1101/gr.1272403

Yen, H. C., Xu, Q., Chou, D. M., Zhao, Z., and Elledge, S. J. (2008) Global protein stability profiling in mammalian cells. *Science* 322, 918–923. doi:10.1126/science.1160489

Yeo, U.-D., Han, J.-Y., Choi, Y.-E., Soh, W.-Y., Nakagawa, N., and Sakurai, N. (1999). Turnover of cell-wau polysaccharides during somatic embryogenesis and development of celery (*Apium graveolens* L.). *J. Plant Biol.*, 42 (I), 8-I5

Yetukuri, L., Ekroos, K., Vidal-Puig, A., and Oresic, M. (2008). Informatics and computational strategies for the study of lipids. *Mol. Biosyst.* 4, 121–127. doi: 10.1039/b715468b

Yi, X., Tesmer, V.M., Savre-Train, I., Shay, J.W., and Wright, W.E. (1999). Both transcriptional and posttranscriptional mechanisms regulate human telomerase template RNA levels. *Mol. Cell. Biol.* 19, 3989 –3997. doi: 10.1128/mcb.19.6.3989

Yokota, K., and Kito, M. (1982). Transfer of the phosphatidyl moiety of phosphatidylglycerol to phosphatidylethanolamine in Escherichia coli. *J. Bacteriol.* 151, 952-961.

Yoon, M., Moir, R.D., Prahlad, V., and Goldman, R.D. (1998). Motile properties of vimentin intermediate filament networks in living cells. *J. Cell Biol.* 143, 147–157. doi: 10.1083/jcb.143.1.147

Youle, R., and Narendra, D. (2011). Mechanisms of mitophagy. *Nat. Rev. Mol. Cell Biol.* 12, 9-14. doi:10.1038/nrm3028

Yuan, J., and Chen, J. (2010). MRE11-RAD50-NBS1 complex dictates DNA repair independent of H2AX. *J. Biol. Chem.* 285, 1097-1104. doi: 10.1074/jbc.M109.078436

Zhang, Z., Qin, Y.W., Brewer, G., and Jing, Q. (2012). MicroRNA degradation and turnover: regulating the regulators. *Wiley Interdiscip. Rev. RNA*. 3, 593-600. doi: 10.1002/wrna.1114

Zhou, M., and Wu, H. (2009). Glycosylation and biogenesis of a family of serine-rich bacterial adhesins. *Microbiology* 155(Pt 2):317-327. doi: 10.1099/mic.0.025221-0

Zhou, W., Jeyaraman, K., Yusoff, P., and Shenolikar, S. (2013). Phosphorylation at tyrosine 262 promotes GADD34 protein turnover. *J. Biol. Chem.* 288, 33146-33155. doi: 10.1074/jbc.M113.504407.

Zhou, Y., and Gottesman, S. (1998). Regulation of proteolysis of the stationary-phase sigma factor RpoS. *J. Bacteriol.* 180, 1154-1158. doi: 10.1128/JB.180.5.1154-1158.1998
