# Supplementary material for: Self-Organization and Information Processing: From Basic Enzymatic Activities to Complex Adaptive Cellular Behavior
Source: Front Genet. 2021 May 21;12:644615. doi: 10.3389/fgene.2021.644615 (PMC8176287; doi:10.3389/fgene.2021.644615)
Supplement: Supplementary file 2 [file Data_Sheet_2.docx]

**Supplementary Material 02**

**2.1. Transfer Entropy**

According to the Information Theory, metabolic patterns might have information which can be captured by Transfer Entropy, allowing the quantification of biomolecular information flows in bits (Schreiber, 2000). In fact, Transfer Entropy (TE) can quantify the reduction in uncertainty that one variable has on its own future when adding another, leading to a calculation of the functional influence between two variables in terms of effective connectivity.

The roots of TE are based on the Shannon Entropy which measures the average information needed to determine a random variable $X$ (Cover and Thomas, 1991). Shannon Entropy can be defined as:

$H\left( X \right)\equiv\sum_{x} -p\left( x \right)\log p(x)$ , Eq. (1)

where $x$ is one of the possible states which characterizes the dynamics of variable $X$. For instance, when tossing a coin, the two only possible states are head or tail. $p(x)$ defines the probability (or normalized occurrence) of measuring the variable $X$ in the state $x$.

The amount of information shared between two variables can be quantified by Mutual Information:

$I\left( X,Y \right)\equiv H\left( X \right)-H(X|Y)$ , Eq. (2)

which measures how much the uncertainty on variable $X$ is reduced by conditioning it to another variable $Y$. Observe that because $H\left( X | Y \right)=H\left( X,Y \right)-H(Y)$, the Mutual Information is a symmetrical measure between $X$ and $Y$, i.e., $I\left( X,Y \right)=I\left( Y,X \right).$

In contrast with Mutual Information, TE is not a symmetrical measure because the TE from $X$ to $Y$ is different from the one from $Y$ to $X$. This asymmetry originates from the fact that to compute the TE one has to define the past and the future on one of the time series, and only the past of the other variable. TE can be defined as:

$TE \left( Y\to X \right)\equiv H\left( X^{future} | X^{past} \right)-H\left( X^{future} | X^{past},Y^{past} \right)$ , Eq. (3)

which allows an easy interpretation of TE. The TE from $Y$ to $X$ measures the amount of uncertainty of the $X^{future}$ that is reduced by knowing the $Y^{past}$ compared to the situation in which only the $X^{past}$ is reducing the uncertainty of $X^{future}$. If by adding $Y^{past}$, the uncertainty of $X^{future}$is reduced more than adding only $X^{past}$, there is a non-zero TE from $Y$ to $X$. Another interpretation of the TE comes after observing Eqs. (2) and (3); the TE is the Mutual Information between $X^{future}$ and $Y^{past}$ conditioning on $X^{past}$, i.e., $TE \left( Y\to X \right)=I\left( X^{future},Y^{past} \right|X^{past})$ .

Therefore, TE enables the quantification of how much the temporal evolution of the activity of one enzyme improves the future prediction of another, and the oscillatory patterns of the biochemical metabolites in the cell may have information which can be read-out by the TE.

**2.2. Yeast glycolysis**

The molecular informative properties of yeast glycolysis, a single dissipative self-organized multienzymatic set, were studied in 2012 *in silico* using Transfer Entropy (De la Fuente and Cortes, 2012). Yeast glycolysis is one of the prototypical biochemical oscillators, and also the most studied metabolic pathway. In fact, it was the first metabolic system in which spontaneous oscillations were observed (Duysens and Amesz, 1957, Chance et al., 1964), and these analyses led to different quantitative models of this pathway based on enzyme kinetics (Goldbeter and Lefeber, 1972; Goldbeter and Lefeber, 1973; De la Fuente et al., 1995; De la Fuente et al., 1996).

As in other dissipative structures, yeast glycolysis dynamics find their roots in non-linear regulatory processes, e.g., allosteric regulation, stoichiometric autocatalysis, product activation (Cortassa et al., 1991, Goldbeter, 2002, Goldbeter, 2007), and other non-linear sources (Cortassa and Aon, 1994, Olsen et al., 2009). It has been shown that in yeast glycolysis a primarily instability-generating mechanism is given by the regulation of phosphofructokinase, in particular, the positive feed-back exerted by its reaction products, ADP and fructose-1,6-bisphosphate (Boiteux et al., 1975, Goldbeter and Lefeber, 1972, Goldbeter, 2002). In yeast extracts, protons and travelling dissipative waves of NADH can also be observed associated with glycolysis (Mair et al., 2001). All this evidence indicates that yeast glycolysis constitutes a good example of a dissipatively structured enzymatic complex that can display spatial and temporal dynamic behavior.

**2.3. Information processing in *Escherichia coli* chemotaxis.**

The *Escherichia coli* chemotaxis network represents one of the best studied examples of a biochemical dynamic system with the capacity to store functional information using molecular information processing linked to post-translational modification dynamics (Greenfield et al., 2009; Li and Stock 2009; Tu et al., 2008; Shimizu et al., 2010; Stock and Zhang, 2013).

Bacterial cell swimmers integrate environmental information accurately in order to make proper decisions for their own survival, in such a way that they can move towards favorable sites and away from unfavorable environments by changing their swimming patterns. As a result of the chemotaxis system, the signal transduction process translates information into appropriate motor responses and the bacterial cells traverse gradients of chemical attractants, displaying an efficient directional sensing and movement by performing temporal comparisons of ligand concentrations. Thus, the cell detects extracellular chemical gradients and senses very small fractional changes, even at nanomolar concentrations (Sourjik and Berg, 2002; Sourjik and Berg, 2004).

Since the pioneering studies by Julius Adler in the 1960’s (Adler, 1966; Adler and Dahl, 1967; Adler and Templeton, 1967), the biochemical mechanisms that underlie sensory-motor regulation in *E. coli* have been extensively investigated and their principal characteristics have been detailed (Baker et al., 2006; Hazelbauer and Falke, 2008). Membrane receptors responsible for signal transduction assemble into large clusters of interacting proteins, shaping a complex modular network (Greenfield et al., 2009; Hamadeh et al., 2011; Shimizu et al., 2010) with one main feedback loop (Emonet and Cluzel, 2008; Wadhams and Armitage, 2004). The network consists of several thousand alpha-helical transmembrane protein fibers that interact with one another forming a cortical structure below the cytoplasmic membrane. The fiber ends that pass through the cytoplasmic membrane interact with a complex layer of sensory receptor domains.

*E. coli* sensory–motor functionality is regulated by two reversible protein modification processes: phosphorylation and carboxyl methylation. The phosphorylation processes provide a direct connection between sensory receptor complexes and motor responses. The transmembrane protein fibers shape a four-helix bundle with at least eight potentially anionic glutamate side chains that can be either exposed as a negative charge or neutralized by methylation. Each fiber can potentially be in any one of 2∧8 different states of modification, and the increments or decrements in attractant concentration produce behavioral responses that lead to changes in methylation dynamics (Stock and Zhang, 2013).

The dynamic changes in chemotaxis network functionality provoke specific molecular information processing and, as a consequence, structural molecular modifications in the form of methylation. They are carried out in such a way that the cell can record the recent chemical past by using this reversible methylation process in determined glutamic acid residues (Li and Stock, 2009). The dynamic methylation-demethylation patterns linked to information processing serve as a structural and functional memory, allowing the detection of attractant gradients by comparing current concentrations to those encountered in the past. The carboxyl methylation mechanism stores information concerning environmental conditions that the bacterium has experienced, and these dynamic patterns of glutamyl modifications act as a structural dynamic memory which allows cells to respond efficiently to continual changes in attractant concentrations (Greenfield et al., 2009; Li and Stock, 2009; Stock and Zhang, 2013).

The *E. coli* chemotaxis network exhibits a rich variety of behavior as well as dynamic properties such as robustness (Kollmann et al., 2005; Alon et al., 1999; von Dassow et al., 2000), signal amplification (Tu, 2013), molecular information processing (Shimizu et al., 2010), fast response, and ultra-sensitive adaptation (Bray et al., 1998; Kollmann et al., 2005; Wadhams and Armitage, 2004; Sourjik, 2004). The essential components of the *E. coli* chemotaxis system are highly conserved among all motile prokaryotes (Wuichet et al., 2007). However, many species have chemotaxis networks that are much more complex than that of *E. coli* (Hamadeh et al., 2011).

**2.3. Information processing in other biochemical processes**

On the other hand, it has been observed information processing at a molecular level in other numerous processes (Ausländer et al., 2012; Daniel et al., 2013) such as the reversible phosphorylation of proteins (Thomson and Gunawardena, 2009); microtubule dynamics (Faber et al., 2006); enzymatic processes (Baron et al., 2006; Katz and Privman, 2010); redox regulation (Dwivedi and Kemp, 2012); transcription (Mooney et al., 1998); genetic regulatory networks (Qi et al., 2013); input signal transduction (Roper, 2007); biochemical networks (Bowsher, 2011); NF-kappaB dynamics (Tay et al., 2010); intracellular signaling reactions (Purvis and Lahav, 2013; Kamimura and Kobayashi, 2012); metabolic switches (Ramakrishnan and Bhalla, 2008); the chemotaxis pathway (Shimizu et al., 2010); network motifs (Alon, 2007), and other cellular processes (Ben-Jacob, 2009).

**References:**

Adler, J. (1966). Chemotaxis in bacteria. *Science*. 153, 708-716. doi: 10.1126/science.153.3737.708

Adler, J., and Dahl, M.M. (1967). A method for measuring the motility of bacteria and for comparing random and non-random motility. *J. Gen. Microbiol*. 46, 161-173. doi: 10.1099/00221287-46-2-161

Adler, J., and Templeton, B. (1967). The effect of environmental conditions on the motility of *Escherichia coli*. *J. Gen. Microbiol*. 46, 175-184. doi: 10.1099/00221287-46-2-175

Alon, U., Surette, M.G., Barkai, N. and Leibler, S. (1999). Robustness in bacterial chemotaxis. *Nature*. 397, 168-117. doi:10.1038/16483

Alon, U. (2007). Network motifs: theory and experimental approaches. *Nat. Rev. Genet.* 8(6):450-461. doi: 10.1038/nrg2102.

Ausländer, S., Ausländer, D., Müller, M., Wieland, M., and Fussenegger, M. (2012). Programmable single-cell mammalian biocomputers. *Nature* 487, 123-127. doi: 10.1038/nature11149

Baker, M.D., Wolanin, P.M., and Stock, J.B. (2006). Signal transduction in bacterial chemotaxis. *Bioessays*. 28, 9-22. doi: 10.1002/bies.20343

Baron, R., Lioubashevski, O., Katz, E., Niazov, T., and Willner, I. (2006). Logic gates and elementary computing by enzymes. *J. Phys. Chem. A*. 110, 8548-8553. doi: 10.1021/jp0568327

Ben-Jacob, E. (2009). Learning from bacteria about natural information processing. *Ann. N.Y. Acad. Sci*. 1178, 78-90. doi: 10.1111/j.1749-6632.2009.05022.x

Boiteux, A., Goldbeter, A., and Hess, B. (1975). Control of oscillating glycolysis of yeast by stochastic, periodic, and steady source of substrate: a model and experimental study. *Proc. Natl. Acad. Sci. U.S.A*. 72, 3829-3833. doi: 10.1073/pnas.72.10.3829

Bowsher, C.G. (2011). Information processing by biochemical networks: a dynamic approach. *J. R. Soc. Interface*. 8, 186-200. doi: 10.1098/rsif.2010.0287

Bray, D., Levin, M.D., and Morton-Firth, C.J. (1998). Receptor clustering as a cellular mechanism to control sensitivity. *Nature*. 393, 85-88. doi: 10.1038/30018

Chance, B., Hess, B., and Betz, A. (1964). DPNH oscillations in a cell-free extract of S. carlsbergensis. *Biochem. Biophys. Res. Commun*. 16, 182-187. doi: 10.1016/0006-291x(64)90358-4

Cortassa, S., Aon, M.A., and Westerhoff, H.V. (1991). Linear non equilibrium thermodynamics describes the dynamics of an autocatalytic system. *Biophys. J.* 60, 794-803. doi: 10.1016/S0006-3495(91)82114-2

Cortassa, S., and Aon, M.A. (1994). Spatio-temporal regulation of glycolysis and oxidative phosphorylation in vivo in tumor and yeast cells. *Cell Biol. Int.* 18, 687-713. doi: 10.1006/cbir.1994.1099

Cover, T.M., Thomas, J.A. (1991). Elements of Information Theory. John Wiley & Sons, Inc., Hoboken, New Jersey (USA).

Daniel, R., Rubens, J.R., Sarpeshkar, R., and Lu, T.K. (2013). Synthetic analog computation in living cells. *Nature* 497, 619-623. doi: 10.1038/nature12148

De la Fuente, I.M., Martínez, L., and Veguillas, J. (1995). Dynamic Behavior in Glycolytic Oscillations with Phase Shifts. *Biosystems* 35:1-13

De la Fuente, I.M., Martínez, L., Veguillas, J., and Aguirregabiria, J.M. (1996). Quasiperiodicity Route to Chaos in a Biochemical System. *Biophys. J.* 71, 2375-2379. doi: 10.1016/S0006-3495(96)79431-6

De la Fuente, I.M., and Cortés, J.M. (2012). Quantitative analysis of the effective functional structure in yeast glycolysis. *PLoS One* 7:e30162. doi: 10.1371/journal.pone.0030162

Duysens, L., and Amesz, J. (1957). Fluorescence espectrophotometry of reduced phosphopyridine nucleotide in intact cells in the near-ultraviolet and visible region. *Biochem. Biophys. Acta* 24, 19-26. doi: 10.1016/0006-3002(57)90141-5

Dwivedi, G., and Kemp, M.L. (2012). Systemic redox regulation of cellular information processing. *Antioxidant. Redox Signal*. 16:374. doi: 10.1089/ars.2011.403

Emonet, T., and Cluzel, P. (2008). Relationship between cellular response and behavioral variability in bacterial chemotaxis. *Proc. Natl. Acad. Sci. U.S.A*. 105:3304-3309. doi: 10.1073/pnas.0705463105

Faber, J., Portugal, R., and Rosa, L.P. (2006). Information processing in brain microtubules. *Biosystems* 83, 1-9. doi: 10.1016/j.biosystems.2005.06.011

Goldbeter, A., and Lefeber, R. (1972). Disipative estructures for an allosteric model. *Biophys. J*. 12, 1302-1315. doi: 10.1016/S0006-3495(72)86164-2

Goldbeter, A., and Lefeber, R. (1973) Patterns of spatiotemporal organization in an allosteric enzyme model. *Proc. Natl. Acad. Sci. U.S.A.* 70, 3255-3259. doi: 10.1073/pnas.70.11.3255

Goldbeter, A. (2002). Computational approaches to cellular rhythms. *Nature* 420, 238-245. doi: 10.1038/nature01259

Goldbeter, A. (2007). Biological rhythms as temporal dissipative structures. *Adv. Chem. Phys*. 135, 253-295.

Greenfield, D., McEvoy, A.L., Shroff, H., Crooks, G.E., Wingreen, N.S., Betzig E., et al. (2009). Self-organization of the *Escherichia coli* chemotaxis network imaged with super-resolution light microscopy. *PLoS Biol*. 7:e1000137. doi:10.1371/journal.pbio.1000137

Hamadeh, A., Roberts, M.A.J, August, E., McSharry, P.E., Maini, P.K., Armitage, J.P., et al. (2011). Feedback control architecture and the bacterial chemotaxis network. *PLoS Comput. Biol*. 7:e1001130. doi:10.1371/journal.pcbi.1001130

Hazelbauer, G.L., Falke, J.J., and Parkinson, J.S. (2008). Bacterial chemoreceptors: high-performance signaling in networked arrays. *Trends Biochem. Sci*. 33, 9-19. doi: 10.1016/j.tibs.2007.09.014

Kamimura, A., and Kobayashi, T.J. (2012). Information processing and integration with intracellular dynamics near critical point. *Front. Physiol*. 3:203. doi: 10.3389/fphys.2012.00203

Katz, E., and Privman, V. (2010). Enzyme-based logic systems for information processing. *Chem. Soc. Rev*. 39, 1835-1857. doi: 10.1039/b806038j

Kollmann, M., Løvdok, L., Bartholomé, K., Timmer J. and Sourjik V. (2005). Design principles of a bacterial signalling network. *Nature* 438, 504-507. doi:10.1038/nature04228

Li, Z., and Stock, J.B. (2009). Protein carboxyl methylation and the biochemistry of memory. *Biol. Chem*. 390,1087-1096. doi: 10.1515/BC.2009.133.

Mair, T., Warnke, Ch., and Muller, S.C. (2001). Spatio-temporal dynamics in glycolysis. *Faraday Discuss.* 120, 249-259. doi: 10.1039/b104106c

Mooney, R.A., Artsimovitch, I., and Landick, R. (1998). Information processing by RNA polymerase: recognition of regulatory signals during RNA chain elongation. *J. Bacteriol*. 180, 3265-3275. doi**:** 10.1128/JB.180.13.3265-3275.1998

Olsen, L.F., Andersen, A., Lunding, A., Brasen, J., and Poulsen, A. (2009). Regulation of Glycolytic Oscillations by Mitochondrial and Plasma Membrane H^+^-ATPases. *Biophys. J.* 96:38503861. doi: 10.1016/j.bpj.2009.02.026

Purvis, J.E., and Lahav, G. (2013). Encoding and decoding cellular information through signaling dynamics. *Cell* 152, 945-956. doi: 10.1016/j.cell.2013.02.005

Qi, H., Blanchard, A., and Lu, T. (2013). Engineered genetic information processing circuits. *Wiley Interdiscip. Rev. Syst. Biol. Med*. 5, 273-287. doi: 10.1002/wsbm.1216

Ramakrishnan, N., and Bhalla, U.S. (2008). Memory Switches in Chemical Reaction Space. *PLoS Comput. Biol*. 4:e1000122. doi:10.1371/journal.pcbi.1000122

Roper, S.D. (2007). Signal transduction and information processing in mammalian taste buds. *Pflügers Arch*. 454, 759-776. doi: 10.1007/s00424-007-0247-x

Schreiber, T. (2000). Measuring information transfer. *Phys. Rev. Lett*. 85, 461-464. doi: 10.1103/PhysRevLett.85.461

Shimizu, T.S., Tu, Y., and Berg, H.C. (2010). A modular gradient-sensing network for chemotaxis in *Escherichia coli* revealed by responses to time-varying stimuli. *Mol. Syst. Biol*. 6:382. doi:10.1038/msb.2010.37

Sourjik, V., and Berg, H. (2002). Receptor sensitivity in bacterial chemotaxis. *Proc.* *Natl. Acad. Sci. U.S.A*. 99, 123-127. doi: 10.1073/pnas.011589998

[Sourjik](https://pubmed.ncbi.nlm.nih.gov/?term=Sourjik+V&cauthor_id=15042093), V., and [Berg](https://pubmed.ncbi.nlm.nih.gov/?term=Berg+HC&cauthor_id=15042093), H.C. (2004). Functional interactions between receptors in bacterial chemotaxis. *Nature* 428, 437-441.  doi: 10.1038/nature02406

Stock, J.B., and Zhang, S. (2013). The biochemistry of memory. *Curr. Biol*. 23, R741-745. doi: 10.1016/j.cub.2013.08.011

Tay, S., Hughey, J.J., Lee, T.K., Lipniacki, T., Quake, S.R., and Covert, M.W. (2010). Single-cell NF-kappaB dynamics reveal digital activation and analogue information processing. *Nature* 466, 267-271. doi: 10.1038/nature09145

Thomson, M., and Gunawardena, J. (2009). Unlimited multistability in multisite phosphorylation systems. *Nature* 460, 274-277. doi: 10.1038/nature08102

Tu, Y. (2013). Quantitative modeling of bacterial chemotaxis: signal amplification and accurate adaptation. *Annu Rev Biophys.* 42, 337-59. doi: 10.1146/annurev-biophys-083012-130358

Tu, Y., Shimizu, T.S., and Berg, H.C. (2008). Modeling the chemotactic response of *Escherichia coli* to time-varying stimuli. *Proc. Natl. Acad. Sci. U.S.A.* 105, 14855-14860. doi: 10.1073/pnas.0807569105

von Dassow, G., Meir, E., Munro, E.M., and Ordell, G.M. (2000). The segment polarity network is a robust developmental module. *Nature* 406, 188-191. doi: 10.1038/35018085

Wadhams, G.H., and Armitage, J.P. (2004). Making sense of it all: bacterial chemotaxis. *Nat. Rev. Mol. Cell. Biol*. 5, 1024-1037. doi: 10.1038/nrm1524

Wuichet, K., Alexander, R.P., and Zhulin, I.B. (2007). Comparative genomic and protein sequence analyses of a complex system controlling bacterial chemotaxis. Methods Enzymol. 422, 1-31. doi:10.1016/S0076-6879(06)22001-9
